# SPE-55 and VHA-18 are essential for FB-MO complex assembly and transport during spermatogenesis in *C. elegans*

**DOI:** 10.1038/s41467-026-75577-6

**Published:** 2026-09-07

**Authors:** Xiaojing Ma, Xinyan Chen, Lianwan Chen, Peng Wang, Xinyue Lu, Xiaolong Zhang, Ying Chen, Ning Wang, Qiushi Wang, Zheng Cao, Yong Liu, Yanmei Zhao, Long Miao

**Affiliations:** 1 MOE Key Laboratory of Cell Proliferation and Regulation Biology, College of Life Sciences, Beijing Normal University, Beijing, China; 2 Key Laboratory of RNA Biology, CAS Center for Excellence in Biomacromolecules, Institute of Biophysics, Chinese Academy of Sciences, Beijing, China; 3 University of Chinese Academy of Sciences, Beijing, China; 4 Anhui Province Key Laboratory of Pollution Damage and Biological Control for Huaihe River Basin, School of Biology and Food Engineering, Fuyang Normal University, Fuyang, China

**Keywords:** Organelles, Developmental biology, Germline development

## Abstract

The spatiotemporal control of asymmetric positioning of cellular cargoes, such as macromolecules and organelles, during spermatogenesis is essential for producing functional sperm in most animal species. However, the regulatory mechanisms that regulate cargo sorting and transporting remain largely unknown. Here, we demonstrate that the casein-like kinase SPE-55 localized on the surface of a membranous organelle (MO) is required for the SPE-18 localization/puncta formation and facilitates the assembly of major sperm protein (MSP) condensate, named the fibrous body (FB), in spermatocytes during spermatogenesis in *Caenorhabditis elegans*. Furthermore, we discover that the H subunit of v-ATPase, which is localized on MOs, played a crucial role in maintaining MO morphology and function, promoting FB–MO connections, and thus ensuring efficient MSP sorting during spermatogenesis. Therefore, the interplay between the membraneless condensate FBs mediated by SPE-55 and the membrane-bound MOs regulated by VHA-18 provides a coordinated mechanism for cargo sorting and directional transport during spermatogenesis in *C. elegans*.

## Abstract (web summary)

This study shows that SPE-55 and VHA-18 coordinate fibrous body–membranous organelle assembly during *C. elegans* spermatogenesis, providing a mechanism for how sperm cargoes are sorted and directionally transported.

## Introduction

Infertility has gradually become a notable issue worldwide. It affects 12–15% of reproductive-aged couples worldwide, and male-related aspects contribute to nearly half of all infertility cases^1,2^. Therefore, it is critical to understand infertility caused by sperm-related factors. However, studies involving human sperm are challenging because of several factors, including the complexity of human-specific biological processes. Numerous model organisms have been employed in the study of male fertility and spermatogenesis. It is easy to perform genetic and cell biology studies in *Caenorhabditis elegans*, which makes it a particularly valuable model in which to investigate sperm function^3,4^. Unlike the spermatozoa in most animals, which typically have whip-like flagella composed of microtubules, the amoeboid spermatozoon of *C. elegans* employs a single pseudopod filled with a gel-like cytoskeletal meshwork composed of the major sperm protein (MSP) to facilitate its motility and migration. MSP is a 14 kDa basic protein that is exclusively expressed in nematode sperm and constitutes approximately 15–20% of the total protein, 40% of the soluble protein in the sperm cell, and roughly 80% of the pseudopod total protein content, thus highlighting its predominant role in its structure and function^5–8^.

Male infertility is largely attributed to abnormal spermatogenesis and/or sperm activation^3,9–11^. Spermatogenesis is a complex process that involves spermatogonia mitotic proliferation, spermatocyte meiotic division, and differentiation of haploid spermatids into mature spermatozoa^9^. During the meiosis II sorting stage of spermatogenesis in *C. elegans*, the developing spermatid containing DNA, some mitochondria, and membranous organelles (MOs) is budded away from the residual body (RB), where unnecessary components, such as the endoplasmic reticulum, ribosomes, tubulin, and actin, are disposed^12^. The MOs, which originate from the Golgi complexes in the spermatocyte^13,14^, are initially observed during the pachytene stage of meiosis I. Subsequently, the MSPs are polymerized into filaments, which are hexagonally packed and assembled on the cytosolic face of the MO, forming the MO-associated fibrous body (FB) complex, named the FB–MO complex. The sequestrated MSPs remain in these FBs via the post-meiotic partitioning process, during which the FB–MO complexes partition vectorially to individual spermatids. The FB–MOs assist in transporting the MSPs and other components into spermatids^13,14^. Once the spermatids bud off the RB, the membrane surrounding the FBs retracts, and the FBs are then disassembled into the cytoplasm. During spermatid activation, the MOs localized underneath the plasma membrane (PM) fuse with the PM, releasing their contents, and the cell polarizes to extend a single pseudopod powered by the cycled assembly/disassembly of the MSP cytoskeleton^6^. Therefore, the assembly of MSPs into membraneless condensate FBs associated with MOs in spermatocytes helps sort MSP into the developing haploid spermatids during budding, thereby guaranteeing that MSPs are transported into spermatids instead of the RB.

However, little is known about the composition of FBs, such as accessory proteins that facilitate MSP assembly and MSP-mediated motility. Furthermore, the underlying mechanisms for selective cargo sorting and transport during spermatogenesis remain largely unknown. In this study, we identified a sperm-specific and casein-like kinase gene *spe-55*, and found that it was necessary for FB assembly during spermatogenesis in *C. elegans*. The null allele or kinase-dead *spe-55* sperm were defective in activation and motility acquisition, thus hermaphrodites and *spe-55* mutant males were sterile. We demonstrated that SPE-55, which was localized to the MOs’ surface in the early stage of spermatogenesis, was essential for SPE-18 (an intrinsically disordered region (IDR)-containing protein necessary for MSP filament bundling) localization/puncta formation and initiating FB assembly. In addition, we discovered that VHA-18, an H subunit of v-ATPase, was localized on MOs and played a crucial role in maintaining MOs’ morphology and function, promoted FB–MO connections, and thus ensured the correct MSP sorting during spermatogenesis. Therefore, this interaction between membraneless condensate FBs, facilitated by SPE-55, and membrane-bound organelles MOs, regulated by VHA-18, reveals a previously unexplored mechanism for cargo sorting and directional transport during spermatogenesis in *C. elegans*.

## Results

### SPE-55 is required for fertility in both hermaphrodites and males

**Fig. 1:**
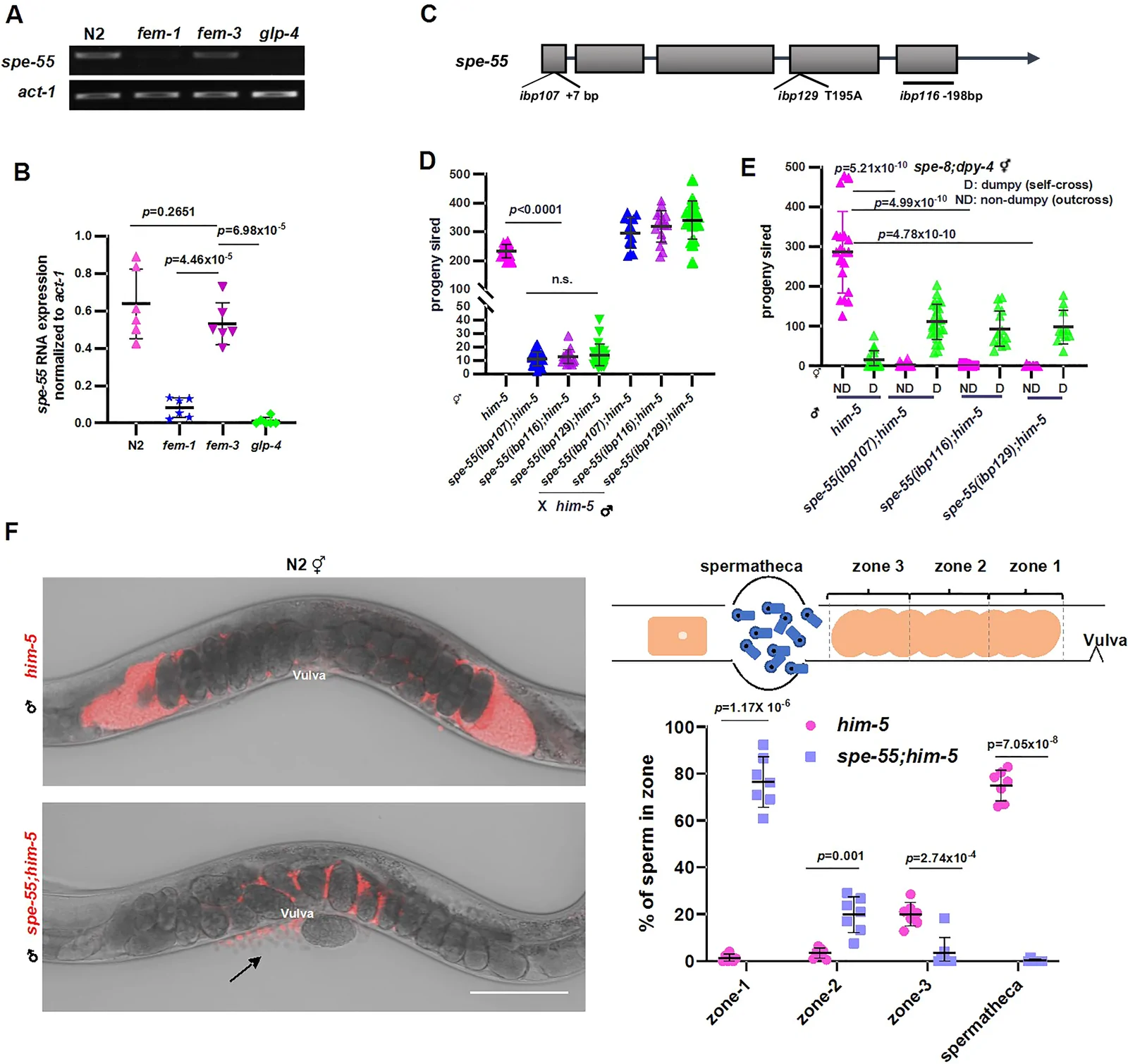
Casein-like kinase SPE-55 is required for *Caenorhabditis elegans* fertility. **A** RT-PCR showed the relative expression of *spe-55* in N2 and *fem-1(hc17), fem-3(q96 gf)*, and *glp-4(bn2)* mutants at day 1 of the adult stage, with *act-1* serving as the control. Uncropped blots was shown in Source Data. **B** Quantification of the relative *spe-55* expression shown in (**A**). Mean ± SD. Two-sided unpaired *t*-test without multiple comparison adjustment. *n* = 3 biological replicates. **C** Genomic structure of the *spe-55* gene. The boxes represent exons, and the solid lines represent introns. The mutations are labeled at their corresponding positions. **D** The *spe-55* mutant hermaphrodites sired fewer progeny compared with the control group, and produced normal numbers of progeny when mated with *him-5* males. n.s. not significant. *n* > 14 worms were counted. Mean ± SD. Two-sided unpaired *t*-test without multiple comparison adjustment. 3 biological replicates. **E** *spe-55* mutant males had impaired fertility. The number of cross progeny (ND, non-Dumpy) and self-progeny (D, Dumpy) produced by *spe-8;dpy-4* hermaphrodites was counted after mating with control *him-5* or *spe-55;him-5* males. *n* > 10 worms were counted. Mean ± SD. Two-sided unpaired *t*-test without multiple comparison adjustment. 3 biological replicates. **F** Sperm ejaculated from *spe-55(ibp107)* males failed to migrate toward and accumulate in the spermatheca. The arrow shows the MitoTracker Red-labeled male sperm from *spe-55;him-5* males located outside of the hermaphrodite uterus. The zones extending from the vulva to the spermatheca were divided into three equal segments (zones 1, 2, and 3 and the spermatheca) in the right image. Quantification is shown in the right. 6 worms were measured in this assay. Scale = 50 μm. Mean ± SD. Two-sided unpaired *t*-test; no multiple comparison adjustment. Source data of (**B**, **D**–**F**) are provided as a Source Data file.

To identify genes that are crucial for spermatogenesis, we screened the gene expression profiles with a specific focus on spermatogenesis in *C. elegans*^15–17^. We identified C09B9.4 and named it *spe-55* (spermatogenetic MSP nucleation kinase-1) based on its function. Because its mutant has a spermatogenesis-defective phenotype (see below). The RT-PCR results revealed that *spe-55* was highly expressed in sperm-producing *fem-3(q96 gf)* worms when compared with those of the mRNA expression level of the oocyte-producing *fem-1(hc17 lf)* and germline-deficient *glp-4(bn2)* worms (Fig. 1A, B). Therefore, the protein encoded by *spe-55* may be involved in spermatogenesis.

SPE-55, like SPE-6, is a member of the greatly expanded *C. elegans* casein kinase 1 superfamily (CK1), with 78 genes in *C. elegans* vs. 12 in humans. Many of these genes are expressed in a spermatogenesis-enriched manner and might be functionally redundant^18,19^. Sequence analysis demonstrated that SPE-55 lacks a transmembrane domain and signal peptide, indicating that SPE-55 is a cytoplasmic protein. BLASTP analysis identified two highly conserved SPE-55 homologs in other *Caenorhabditis* species, *C. brenneri* and *C. remanei* (Supplementary Fig. 1A). The SPE-55 protein contains 359 amino acids, with a conserved Ser/Thr kinase catalytic domain and a flexible C-terminal tail. In most kinases, the activation loop begins with the DFG residues in the catalytic domain and then undergoes a conformational change upon the phosphorylation of Ser, Thr, or Tyr residues within the loop, which makes the ATP and substrates accessible for the active site. Sequence alignment of *spe-55* with *ck1* in other species identified a conserved Thr195 in the activation loop of SPE-55 (Supplementary Fig. 1A).

We generated a frameshift mutation allele *ibp107*, which has a 7-bp insertion in the first exon of the *spe-55* gene, using the CRISPR-Cas9 genome editing system (Fig. 1C and Supplementary Fig. 1B). The *spe-55* allele used throughout this study, including in all unlabeled panels, is *ibp107*, a frameshift mutation. We then assessed whether the mutation affected the worms’ fertility. Each control hermaphrodite could produce brood sizes of more than 200 (mean ± SD is 231.90 ± 22.91), while each *spe-55(ibp107)* hermaphrodite only produced brood sizes of about 11 (mean ± SD is 11.12 ± 4.94) because of defective sperm or oocytes, thus making them nearly sterile (Fig. 1D).

However, *spe-55(ibp107)* hermaphrodites exhibited no significant difference compared with the control *him-5* hermaphrodites when mated with *him-5* males, which indicates that the *spe-55* infertility defect is due to defective sperm and not defective oocytes. In addition, unfertilized oocytes were observed on the plates of *spe-55(ibp107);him-5* hermaphrodites (Supplementary Fig. 1C). We also generated two other alleles, *ibp129*, in which the conserved T195 residue in the kinase activation loop is replaced with A, and *ibp116*, in which the C-terminal tail was deleted (Fig. 1C and Supplementary Fig. 1B). The fertility of hermaphrodites with these two alleles exhibited no significant difference when compared with the *ibp107* allele (Fig. 1D). The average number and SD (presented as mean ± SD) produced by *spe-55(ibp107);him-5,spe-55(ibp116);him-5* and *spe-55(ibp129);him-5* hermaphrodite’s groups were 11.12 ± 4.94, 12.64 ± 5.13, 13.92 ± 7.98, respectively; These findings imply that SPE-55 may have a sperm-specific function in hermaphrodites. Furthermore, *spe-55(ibp107)* is not a temperature-sensitive strain because the fertility of *spe-55* hermaphrodites showed no difference at 16, 20, or 25 °C (Supplementary Fig. 1D).

To evaluate male fertility, the males were mated with the self-sterile *spe-8;dpy-4* hermaphrodites. The *spe-8* hermaphrodites are infertile because of the abnormality in their sperm activation^20^. However, their self-sterility can be restored to wild-type levels by mating them with males because the *spe-8* hermaphrodite sperm can undergo trans-activation by protease TRY-5 in the seminal fluid from males^10^. Moreover*, dpy-4* is a recessive mutant that is used to distinguish between cross- and self-progeny^21^. Male sperm have a competitive advantage over hermaphrodite sperm^22–24^. Most progeny would be cross progeny and exhibit a non-dumpy phenotype when wild-type (WT) male sperm are used after mating. Trans-activated hermaphrodite sperm would only be used to produce self-progeny and thus exhibit a dumpy phenotype when the sperm from males are depleted or defective. When control *him-5* males were used to mate with *spe-8;dpy-4* hermaphrodites, the majority of progeny were cross progeny (non-dumpy). However, when *spe-55(ibp107);him-5* males or two other mutants, such as *ibp129;him-5* or *ibp116;him-5*, were mated with *spe-8;dpy-4* hermaphrodites, almost all progeny were self-progeny (dumpy) (Fig. 1E). These results indicate that, although the seminal fluid contains a functional sperm activator, sperm from *spe-55* males exhibit a severe functional defect and fail to outcompete hermaphrodite-derived sperm. Therefore, the loss or inactivation of SPE-55 significantly reduces the number of offspring due to sperm defects.

Next, we analyzed the motility of sperm ejaculated by *spe-55* males into the hermaphrodite reproductive tract. Males that were stained with MitoTracker Red (a dye that labels mitochondria in live cells) were mated with non-labeled N2 hermaphrodites. After mating, the MitoTracker Red-stained sperm from males were transferred to the hermaphrodites and became activated. The sperm from the control males (*him-5*) had the ability to crawl toward and accumulate in the spermatheca or adjacent zones to prepare for fertilization. In contrast, sperm from the *spe-55;him-5* males remained dispersed near the vulva (Fig. 1F), thus indicating that sperm from *spe-55;him-5* males are defective in motility. Together, these results demonstrate that SPE-55 is required for fertility in both hermaphrodites and males and that the loss of SPE-55 results in sperm-specific defects, such as sperm activation and/or migration after ejaculation.

### SPE-55 is required for sperm activation during spermiogenesis

**Fig. 2:**
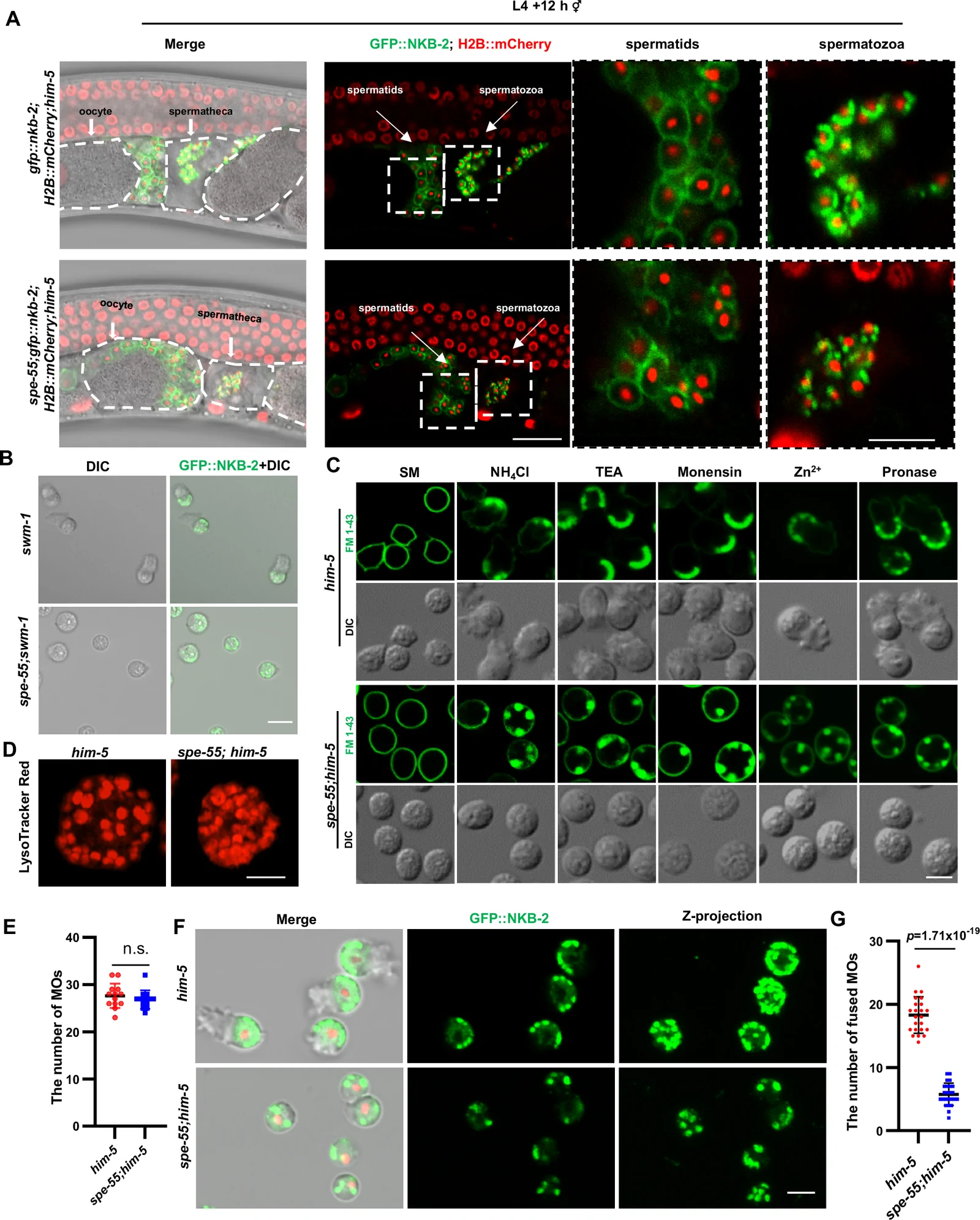
*spe-55* affects sperm activation and motility. **A** Loss of *spe-55* may affect hermaphrodite sperm activation, which was demonstrated by the GFP puncta fluorescence on spermatozoa located in the spermatheca. Scale bars in the left and right panels represent 20 and 10 μm, respectively. 3 biological replicates with similar results. **B** The dissected sperm from male gonads of *spe-55;swm-1;gfp::nkb-2;him-5* mutants and the control group. In vivo activated *spe-55* sperm exhibited almost no pseudopods, although GFP::NKB-2 displayed a polarized distribution pattern. Scale = 5 μm. 3 biological replicates with similar results. **C** *spe-55* affected sperm activation in vitro with extracellular sperm activators. Sperm medium (SM) served as the inactivated control. The membranous organelle (MO) fusions were indicated by the fluorescent puncta of membrane-impermeable probe FM 1–43. Scale = 5 μm. 3 biological replicates with similar results. **D** *spe-55* spermatids contained normal MOs as seen in the control. The acidic MOs were labeled with LysoTracker Red. Scale = 5 μm. **E** Quantification of the number of MOs indicated by LysoTracker Red shown in (**D**). n.s. not significant. 13 sperm were measured. Mean ± SD. Two-sided unpaired *t*-test without multiple comparison adjustment. 3 biological replicates. **F** *spe-55* affected MO fusion and pseudopod extension when sperm were activated by pronase. The fluorescent puncta of GFP::NKB-2 were used to indicate the fused MOs. Scale = 5 μm. 3 biological replicates with similar results. **G** Quantification of the number of fused MOs indicated by GFP::NKB-2 puncta fluorescence in (**F**). 23 sperm were measured. Data are shown as means ± SD. Two-sided unpaired *t*-test; no multiple comparison adjustment. 3 biological replicates. Source data above are provided as a Source Data file.

To determine how the *spe-55* mutation affects sperm function, we crossed *spe-55*(*ibp107*);*him-5* worms with worms that express GFP::NKB-2 and H2B::mCherry to detect spermatogenesis and sperm activation in vivo. NKB-2 is the β subunit of Na^+^/K^+^ ATPase that is uniformly localized on the PM of spermatocytes and spermatids^25^. During sperm activation, Na^+^/K^+^ ATPase is gradually translocated to and enters the fused MOs^25^. In addition, the Histone H2B (H2B) was tagged with mCherry, and its detection indicated that Histone H2B was prevalent in the nucleus. In hermaphrodites, no obvious GFP::NKB-2;H2B::mCherry fluorescence difference was observed before the first ovulation (Supplementary Fig. 2A). After ovulation, hermaphrodite sperm were pushed into and activated in the spermatheca. In the enlarged spermatozoa region in Fig. 2A, however, the GFP::NKB-2 signal in the *spe-55* mutant appeared some different, though may be not significant, relative to the control. Besides, *spe-55* mutant male spermatozoa have severe motility defects which may be caused by sperm activation. All these provided a cue for us to test the impact of the *spe-55* mutation on spermiogenesis.

Next, we evaluated spermatogenesis by observing the distribution of GFP::NKB-2 in males. There was no obvious difference in the number of spermatids produced between the *spe-55* mutant and WT animals (Supplementary Fig. 2B). This indicates that the *spe-55* mutant does not cause any immediately apparent defect in male germline development. Subsequently, we investigated spermiogenesis in vivo using *swm-1* mutant males, which would undergo precocious activation due to the loss of an activation inhibitory signal^21^. We performed a time-lapse analysis and observed motile spermatozoa that were ectopically activated with punctate GFP::NKB-2 fluorescence in the seminal vesicle of control *swm-1;him-5* males (Supplementary Video 1). However, in *spe-55;swm-1;him-5* males, the incompletely activated sperm with polarized GFP::NKB-2 puncta remained immotile in the seminal vesicle (Supplementary Video 2). To characterize this result in an in situ or an orthotopically activating environment, we imaged GFP::NKB-2–labeled sperm from WT and *spe-55* mutant males within the uterus of feminized *fog-2* worms. Sperm from the *spe-55* mutant males rarely accumulated in the spermatheca and instead remained dispersed throughout the uterus, whereas the control sperm were predominantly accumulated within the spermatheca (Supplementary Videos 3 and 4). This observation was consistent with the results presented in Fig. 1F, which indicated that sperm from *spe-55;him-5* mutants exhibited defective motility. After dissecting the sperm from the aforementioned males, we observed pseudopod formation and fully polarized GFP::NKB-2 puncta in sperm from the control *swm-1;him-5* group but not from the *spe-55;swm-1;him-5* group (Fig. 2B). This indicates that the *spe-55* mutation affects sperm activation and motility in vivo.

Furthermore, we isolated spermatids from the male seminal vesicle and treated them with artificial sperm activators, such as the weak base NH_4_Cl, TEA, the proton carrier monensin, the microelement Zn^2+^, or pronase (Fig. 2C). FM1-43, a non-permeable membrane dye, was used to indicate the PM or the fused MOs because the fusion pores allow the dye to enter into the permanent invaginations and stain the body of the MOs. The sperm from the *him-5* males were responsive to these extracellular activators, displaying a pseudopod and several fused MOs on the cell body side. However, *spe-55;him-5* sperm failed to fully activate, exhibiting less fused MOs and almost no pseudopod formation (Fig. 2C). Moreover, sperm from *spe-55;him-5* mutant males that were treated with the same in vitro activators resulted in the similar phenotype (Fig. 2C). Taken together, these results suggest that *spe-55* mutant sperm are defective in the fusion of MO with PM and pseudopod extension during sperm activation.

To further identify whether SPE-55 affects the quantity of MOs in spermatogenesis or MO fusion during sperm activation, the acidic MOs were labeled with LysoTracker Red^26^. We found that the number of MOs was normal in *spe-55;him-5* sperm when compared with that of the *him-5* control (Fig. 2D, E). Upon activation with pronase, all sperm exhibited a response; however, those of the *spe-55;him-5* mutant displayed a significant reduction in the number of fused MOs and remained round. This contrasted sharply with the *him-5* spermatozoa, which developed an elongated pseudopod and exhibited a substantially greater number of fused MOs within the cell body (Fig. 2F, G). These observations suggested that sperm activation is weak and defective in the *spe-55* mutant background. Taken together, these results indicate that SPE-55 is required for sperm activation in vivo and in vitro and that the loss of SPE-55 affects MO fusion and polarity establishment to form a single pseudopod during spermiogenesis.

### *spe-55* mutants produce spermatocytes without FB formation

**Fig. 3:**
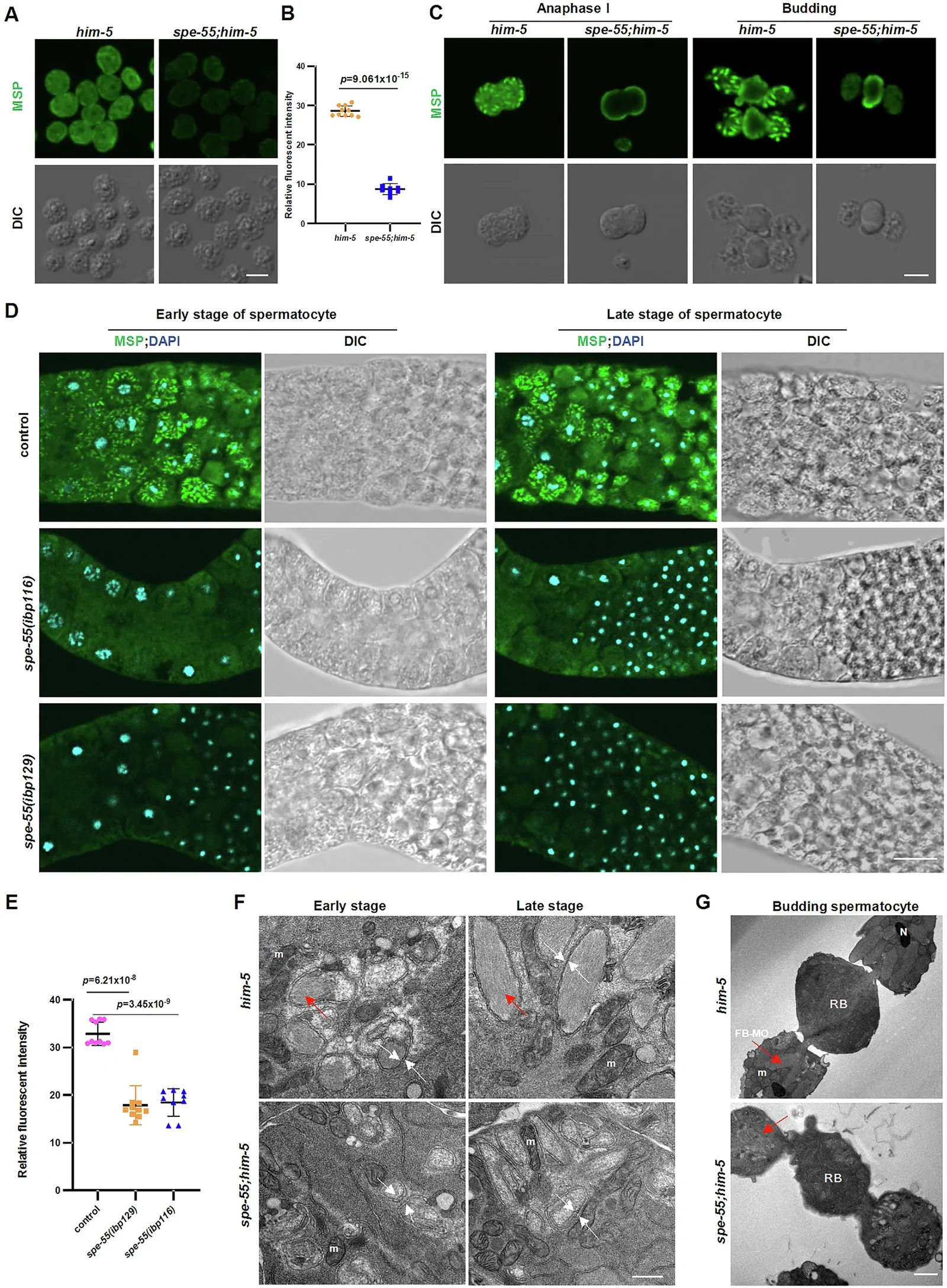
*spe-55* affects FB assembly in spermatocytes. **A** The anti-major sperm protein (MSP) immunofluorescence microscopy assay of spermatids from *spe-55(ibp107)* and control males. Scale = 5 μm. 3 biological replicates with similar results. **B** Quantification of MSP fluorescence intensity in sperm from (**A**). *n* = 10 sperm were measured. Data are shown as means ± SD. Two-sided unpaired *t*-test; no multiple comparison adjustment. **C** The anti-MSP immunofluorescence assay of spermatocytes from *spe-55(ibp107)* and control males at different stages. Scale = 5 μm. 3 biological replicates with similar results. **D** The anti-MSP immunofluorescence microscopy assay of male gonads of *spe-55(ibp116), spe-55(ibp129)*, and control worms. Scale = 20 μm, 3 biological replicates with similar results. **E** Quantification of MSP fluorescence from worm gonads shown in (**D**). *n* = 6 spermatocytes exhibiting prominent FB–membranous organelle (MO) structures in the corresponding wild-type region of the *spe-55;him-5* mutant. Data are shown as means ± SD. Two-sided unpaired *t*-test; no multiple comparison adjustment. **F** Electron microscopy (EM) images of spermatocytes from *spe-55(ibp107)* and control males. White arrows: tubule-like MOs; red arrows in the top panels: FBs semi-encased by MOs; m mitochondria. Scale = 500 nm. 3 biological replicates with similar results. **G** EM images of sperm and RB in the budding stage from *spe-55(ibp107)* and control males. Red arrows: FB–MO complex in control spermatids and the corresponding section in *spe-55;him-5;* m mitochondrion, N nucleus, RB residual body, Scale = 1 μm. 3 biological replicates with similar results. Source data of (**B**) and (**E**) are provided as a Source Data file.

The failure of pseudopod formation in *spe-55* sperm during activation could be due to defects in MSP formation, MSP cytoskeleton assembly, or the sorting of FBs into spermatids during spermatogenesis. To test this hypothesis, we performed an immunofluorescence microscopy (IFM) assay with an anti-MSP monoclonal antibody. We found the MSP level in *spe-55(ibp107)* spermatids was significantly lower than that in the control group (Fig. 3A, B). Then, we evaluated the MSP distribution in spermatocytes and found that there was no MSP assembly in the FB of *spe-55(ibp107)* spermatocytes. Instead, the MSP in *spe-55(ibp107)* spermatocytes was diffusely distributed in the cortical area. (Fig. 3C). In addition, the *spe-55(ibp129) or spe-55(ibp116)* alleles also affected the FB formation in spermatocytes (Fig. 3D, E), indicating that the kinase activity of SPE-55 is required for FB formation in spermatocytes.

In addition, we analyzed the ultrastructure of spermatocytes using transmission electron microscopy (TEM). We selected two representative images of the early and late stages of spermatocytes, according to the FB–MO morphology. The MSP filaments were neatly arranged as FBs and semi-surrounded by the tubule-like MO in WT spermatocytes. However, we observed a marked reduction or complete absence of inclusions of the tubule-like MOs in *spe-55* mutant spermatocytes, even during the early stages of spermatocyte development (Fig. 3F). In the budding stage, obvious FB–MO structures were observed in the dividing spermatids in the WT group but not in the *spe-55;him-5* mutants (Fig. 3G). These TEM results may imply that SPE-55 loss may affect MSP formation or MSP assembly into FBs. We then compared the mRNA level of every MSP gene detected in *spe-55;him-5* with those in the *him-5* group, and a significance criterion of fold change ≥2 or ≤0.5 was applied. No significant differences were observed between these groups (Supplementary Fig. 3A). Considering that small reductions in the expression of individual genes could collectively result in an overall decrease in protein levels, the current results cannot exclude the possibility that SPE-55 loss may affect the total content of MSP (Supplementary Fig. 3A). Therefore, we conclude that *spe-55* mutants produce spermatocytes without FB formation. However, much less MSP could exist in the spermatids, which is consistent with the above phenotype that sperm failed to extend a pseudopod during sperm activation.

### SPE-55 is recruited to the MO surface to initiate FB assembly

**Fig. 4:**
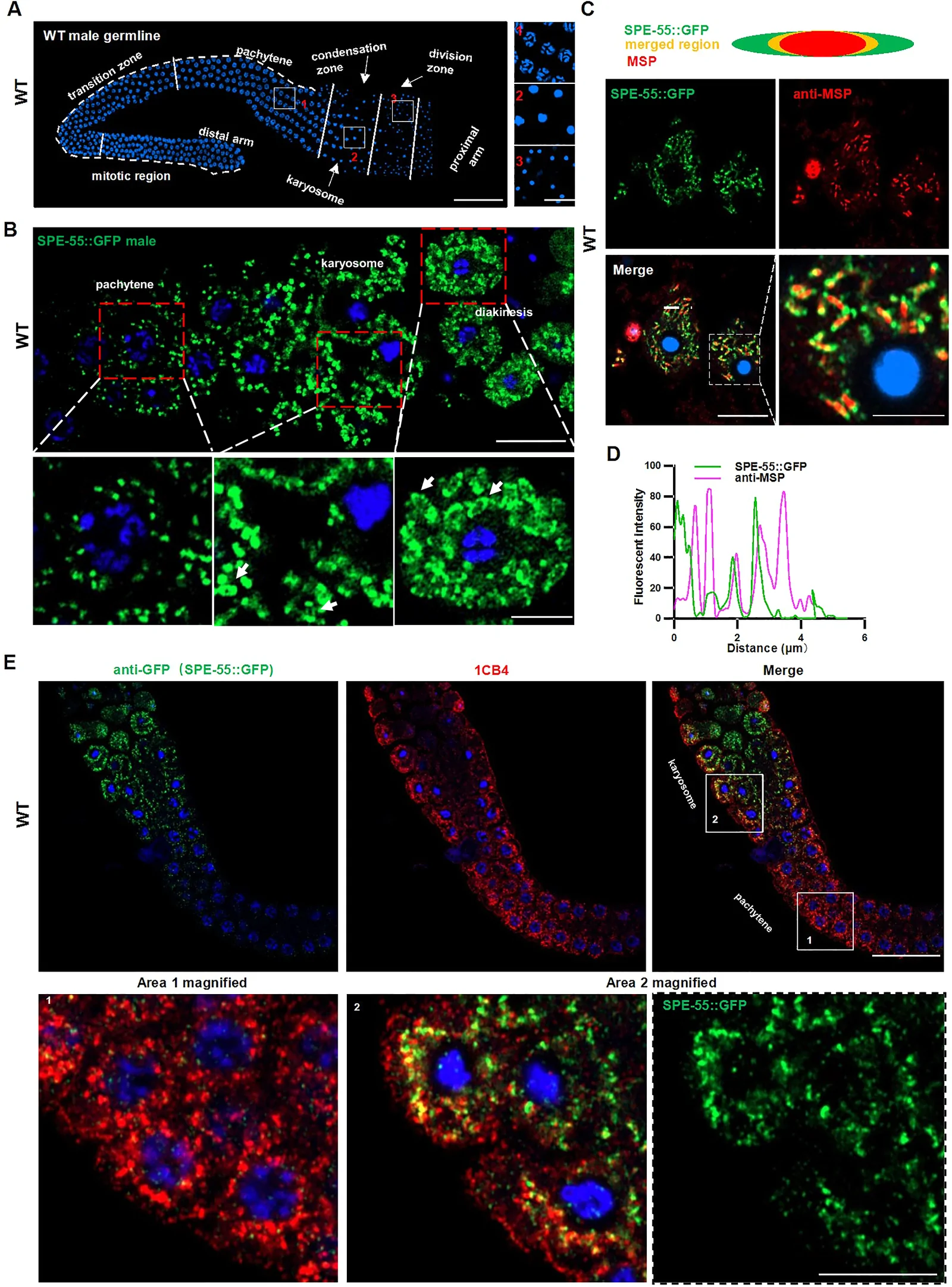
Punctate SPE-55 is associated with FBs in spermatocytes. **A** The shape and size of the nuclei labeled by Hoechst illustrated the various developmental stages in the wild-type male gonad. Scale bars in the left and right panels represent 50 and 20 μm, respectively. 3 biological replicates with similar results. **B** The expression and localization pattern of SPE-55::GFP in the male gonad. The arrow shows the boat-shaped structure of the GFP signal. The nuclei were labeled with Hoechst. Scale bars in the upper and lower panels represent 10 μm. 3 biological replicates with similar results. **C** Immunofluorescence assay shows that SPE-55(green) was localized to the periphery of fibrous bodies (FBs), which were immuno-stained by anti-major sperm protein (MSP) (red), in meiotically dividing spermatocytes. The nuclei were labeled with DAPI. Scale bars in the left and right panels represent 20 and 10 μm, respectively. 3 biological replicates with similar results. **D** Quantification of MSP and SPE-55::GFP fluorescence in the dashed line shown in (**C**). Source data are provided as a Source Data file. **E** The immunofluorescence analysis showed the localization of 1CB4 (red, MO marker) and SPE-55::GFP puncta in the male gonad. Box 1 (pachytene stage) indicates the area where 1CB4 labeling was visible, but SPE-55::GFP fluorescence was absent. Box 2 (karyosome stage) indicates the area where SPE-55::GFP puncta (green) were partially colocalized with the membranous organelle (MO) (red). Scale bars in the left and right images represent 20 and 10 μm, respectively. 3 biological replicates with similar results.

The male gonad undergoes a developmental process from the distal to the proximal arm. Moreover, mitosis occurs in the distal region, followed by entry into meiosis after passing through the transition zone. During meiosis, a prolonged pachytene occurs, which is characterized by chromosomal recombination and extensive spermatogenesis-related gene expression. Subsequently, the nucleus condenses into a distinct entity characterized by substantially inhibited transcription. This is followed by a brief and rapid meiotic division stage, resulting in the formation of spermatids^27^. The various stages could be distinguished by nuclear morphology revealed with Hoechst staining (Fig. 4A). We generated a GFP-tagged *spe-55* variant using CRISPR-Cas9-based genome editing. The sperm-specific distribution pattern of SPE-55::GFP fluorescence was observed at the proximal terminal of the germline in males and at the spermatheca, where the sperm is stored in hermaphrodites (Supplementary Fig. 4A). In the pachytene stage, SPE-55::GFP fluorescence exhibited a punctate pattern that gradually expanded. In the karyosome stage of prophase I, when the FBs begin to assemble, the SPE-55::GFP fluorescence formed a boat-shaped pattern, which then enlarged in size during meiotic divisions (Fig. 4B). During the budding stage after anaphase II, most of the SPE-55::GFP fluorescent signals were partitioned into spermatids and then diffused into the cytosol when budding finished (Supplementary Fig. 4B).

Given that FBs are usually associated with MOs^25,26,28–31^, and that SPE-55 loss affects FB formation in spermatocytes, we hypothesized that the boat-shaped SPE-55::GFP fluorescence signal in spermatocytes is related to FB–MOs in the developing spermatocytes. To test this hypothesis, we performed an IFM assay. The FBs reacted with the MSP antibodies and were associated with the GFP-labeled SPE-55, where they displayed a partial colocalization pattern, especially at the FB ends (Fig. 4C, D). In spermatocytes, FBs are semi-enclosed by the membranes of Golgi-derived MOs^5,30,32^; therefore, we checked the localization of SPE-55::GFP relative to the MOs, which could be recognized by its fiducial marker 1CB4^25,33–35^. We found that the presence of 1CB4-labeled MOs occurred substantially earlier than that of SPE-55::GFP. Furthermore, SPE-55::GFP exhibited partial colocalization with MOs (Fig. 4E).

### SPE-55 functions upstream of SPE-18

**Fig. 5:**
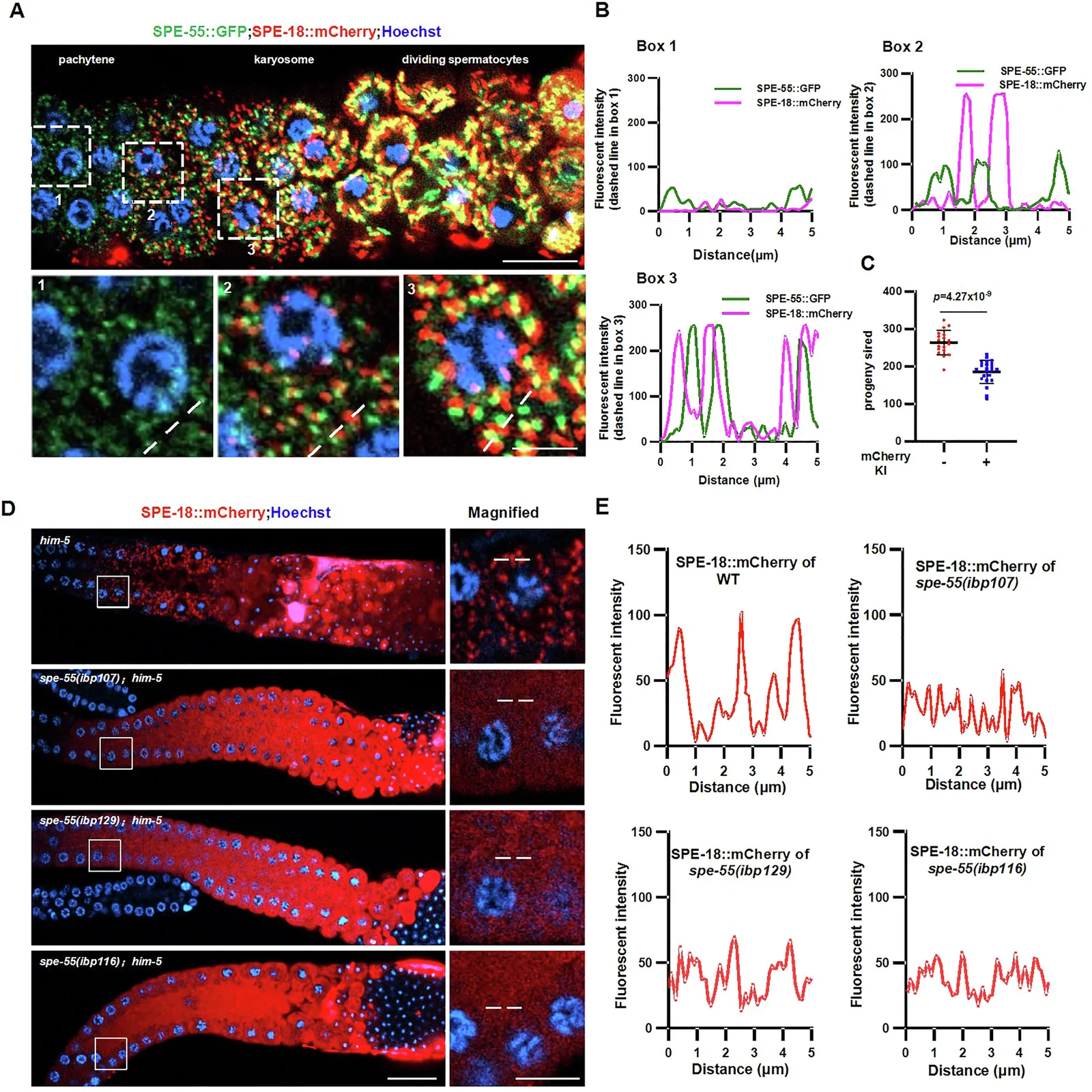
SPE-55 functions upstream of SPE-18 and is required for recruiting SPE-18 to membranous organelles (MOs). **A** Live-cell imaging of SPE-55::GFP and SPE-18::mCherry in different stages of spermatogenesis, indicated by the nuclear size and structure. The boxed areas in the upper panel were magnified in the lower panels. Scale bars in the upper and lower panels represent 10 and 5 μm, respectively. 3 biological replicates with similar results. **B** Quantification of SPE-18::mCherry and SPE-55::GFP fluorescence intensity along the dashed line shown in (**A**). The images correspond to the dashed lines in magnified areas 1, 2, and 3, respectively. **C** Inserting mCherry into the C-terminal of SPE-18 affected the hermaphrodites’ fertility. *n* > 18 worms were counted. Data are shown as means ± SD. Two-sided unpaired *t*-test; no multiple comparison adjustment. 3 biological replicates. **D** SPE-55 loss affected the localization of SPE-18::mCherry during spermatogenesis. The boxed areas on the left are magnified on the right. Scale bars in the left and right panels represent 20 and 10 μm, respectively. 3 biological replicates with similar results. **E** Quantification of SPE-18::mCherry fluorescence intensity in the dashed lines shown in (**D**). Source data above are provided as a Source Data file.

The expression pattern of SPE-55 is similar to that of the SPE-18 protein, which, together with SPE-6, participates in MSP polymerization and FB formation^18,28,29^. SPE-18 localizes to developing spermatocytes to facilitate MSP nucleation and assembly. We aimed to determine if there is a relationship between the expression pattern of SPE-18 and SPE-55. We generated SPE-18::mCherry, and *spe-18* frameshift mutation strains using CRISPR-Cas9-based genome editing technology. We observed that the SPE-55::GFP signal appeared earlier than the SPE-18::mCherry signal during the pachytene stage (Fig. 5A, B). Once SPE-18::mCherry became detectable, some SPE-55::GFP signals were initially adjacent to the SPE-18::mCherry puncta. As development progressed to the karyosome stage, SPE-55::GFP and SPE-18::mCherry puncta were partially colocalized. (Fig. 5A, B and Supplementary Fig. 5A). Moreover, the SPE-18::mCherry hermaphrodites showed a slight decrease in fertility (Fig. 5C).

We speculated that SPE-55::GFP might function upstream of SPE-18::mCherry during FB assembly, given their spatially similar but temporally different localization patterns during meiosis. SPE-55 loss or inactivation completely disrupted the SPE-18::mCherry puncta in spermatogenesis (Fig. 5D, E). Conversely, the *spe-18* mutation did not affect the punctate structure of SPE-55::GFP in prophase I of meiosis. (Supplementary Fig. 5B). Combined with the study that SPE-18 is recruited to MOs^29^, we conclude that MO-localized SPE-55 functions upstream of SPE-18 and hypothesize that SPE-55 could directly or indirectly recruit SPE-18 to the surface of MOs to collaboratively initiate the packing of MSP filaments into FBs.

### MO acidification mediated by VHA-18 is associated with FB–MO morphogenesis

**Fig. 6:**
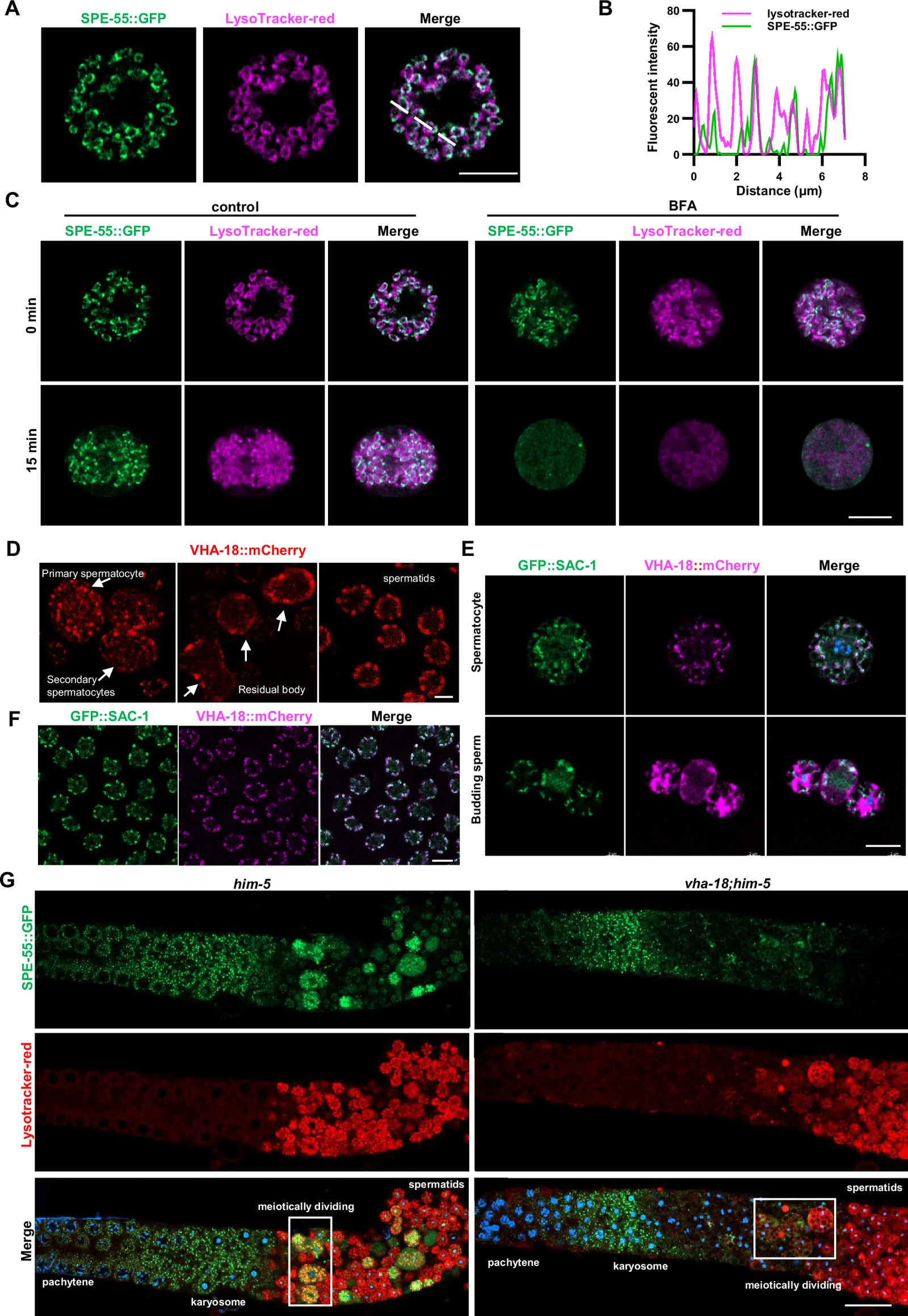
Acidification of the MO lumen mediated by v-ATPase is required for FB–MO morphogenesis. **A** SPE-55::GFP puncta were localized on the surface of acidic MOs probed with the LysoTracker Red in the primary spermatocyte dissected from males germline. Scale = 5 μm. 3 biological replicates with similar results. **B** Quantification of the SPE-55::GFP and LysoTracker Red fluorescence intensity in the dashed line shown in (**A**). Source data is provided as a Source Data file. **C** BFA treatment neutralized the lumen of MOs and caused the puncta of SPE-55::GFP to diffuse in spermatocytes. Scale bar 5 μm. *n* = 3 biological replicates. **D** The expression pattern of VHA-18::mCherry in the primary and secondary spermatocytes, residual body, and spermatids. Scale bar 5 μm. *n* = 3 biological replicates. **E** VHA-18::mCherry was expressed and formed puncta associated with MOs (marked by GFP::SAC-1) in spermatocytes and budding sperm. Scale bar 5 μm. *n* = 3 biological replicates. **F** VHA-18::mCherry puncta partially colocalized to MOs as indicated by GFP::SAC-1 in the spermatids. 15 males were dissected to obtain sperm, three biological replicates. Scale bar 5 μm. **G** VHA-18 loss resulted in swollen MOs dispersed throughout the cytoplasm and impaired condensation of SPE-55::GFP in spermatocytes. Many big, round, and scattered swollen MOs were not stained by LysoTracker Red in *vha-18* spermatocytes. Meanwhile, the cytoplasmic space between the swollen MOs was stained heavily by LysoTracker Red, instead of the MO lumen observed in the control. The fluorescence of SPE-55::GFP appeared as puncta at the distal end but decreased dramatically and failed to colocalize with LysoTracker Red in the area of spermatocytes in the proximal germline of *vha-18* male. The white boxes indicate the spermatocytes undergoing meiotic divisions. Scale bar 5 μm. *n* = 3 biological replicates.

Membraneless condensate FBs and membrane-bound MOs are closely interrelated during spermatogenesis. Each acidic MO (stained with LysoTracker Red)^26^ was associated with the SPE-55::GFP puncta in spermatocytes (Fig. 6A, B). The highly acidic pH of MOs’ lumen implies that an appropriate pH microenvironment might be essential for the morphogenesis of FB–MO. Vacuolar H^+^ ATPases (v-ATPases) are ATP-dependent proton pumps that transport H^+^ across membranes into the vesicular lumens, using energy from ATP hydrolysis^36,37^. The v-ATPase complex consists of the soluble V1 domain, which hydrolyzes ATP, and the membrane-embedded V0 domain, which is responsible for proton transport. The v-ATPase activity supports intracellular substance transport and homeostasis. Previous studies have revealed that SPE-5, which is a B subunit of v-ATPase, is specifically expressed in spermatogenesis in *C. elegans* and that the loss of SPE-5 affects MO morphology and acidification in spermatocytes and spermatids^12,26^.

**Fig. 7:**
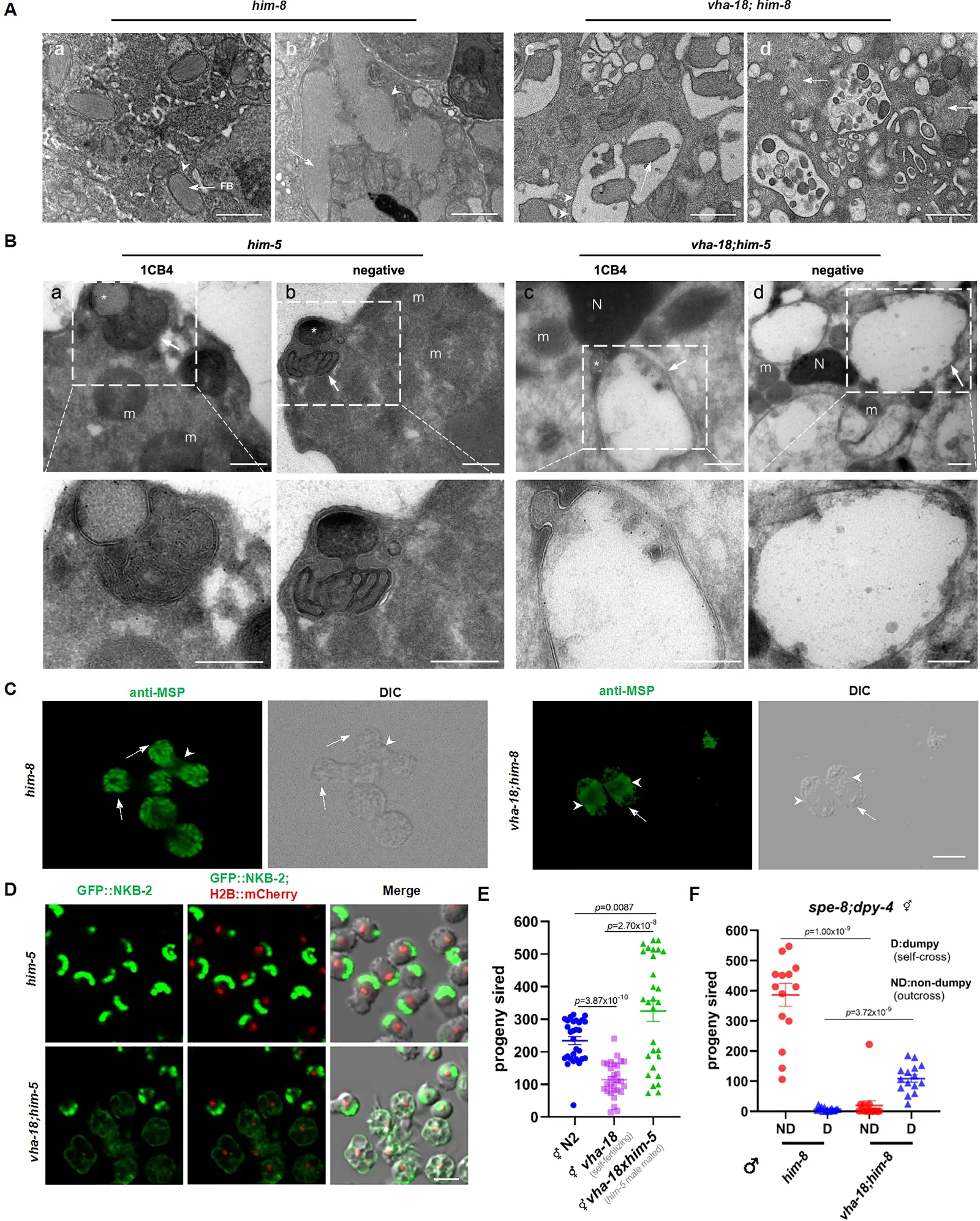
VHA-18 is required for FB–MO complex morphogenesis and male fertility. **A** TEM analysis of spermatocytes from *him-8* control and *vha-18;him-8* male gonads. **a**, **c** and **b**, **d** represent the early and late stage of spermatogenesis, respectively. White arrows: FB; arrowheads: MO. Scale bar 800 nm. Shown are representative images of three independent experiments. **B** Cryo-immuno EM analysis of spermatids. The smaller MO head (**c**) and swollen MO body (**c**, **d**) are observed in *vha-18;him-5*, and the normally sized MO head and body are observed in *him-5* control (**a**, **b**). The boxed areas in the upper panel were magnified in the lower panels. Gold particles were present in **a** and **c** but absent in **b** and **d**. White arrow: MO body; asterisk: MO head; m mitochondria, N nucleus. Scale bar 400 nm. Shown are representative images of three independent experiments. **C** IFM analysis of sperm. MSP signal is visible in the RB and budding spermatids in *vha-18;him-8*, and visible in the budding spermatids but barely in RB in *him-8* control. White arrow: budding spermatids, arrowhead: RB. Scale bar 5 μm. Shown are representative images of three independent experiments. **D** The isolated sperm were activated with TEA in vitro, and spermatozoa from *vha-18;him-5* mutant displayed less GFP::NKB-2 puncta and shorter pseudopods or spiky projections than those from control *him-5*. Scale bar 5 μm. Shown are representative images of three independent experiments. **E** *vha-18* mutant hermaphrodites sired fewer progenies than control N2, and their fertilities were restored by mating with *him-5* males. **F** The number of outcross- or self-progeny produced by *spe-8;dpy-4* hermaphrodites was counted after mating with *him-8* or *vha-18;him-8* males. 30 (**E**) and 15 (**F**) worms were measured. **E**, **F** data are mean ± SEM. Two-sided unpaired *t*-test; no multiple comparison adjustment applied. 3 biological replicates. Source data of (**E**) and (**F**) are provided as a Source Data file.

To investigate the role of MO acidification in the MSP condensate assembly, we treated the spermatocytes with Bafilomycin A1 (BFA), which specifically binds to the C subunit of v-ATPase and inhibits its activity^38,39^. BFA treatment disrupted MO acidification in meiosis II of a single dissected spermatocyte and affected the punctate structure of SPE-55::GFP (Fig. 6C). Our genetic screening identified a highly conserved H subunit of v-ATPase, VHA-18 (Supplementary Fig. 6A), that is required for spermatogenesis^40^. Furthermore, we generated an mCherry knock-in allele at the *vha-18* locus using CRISPR-Cas9-based genome editing. In the knock-in worms, VHA-18::mCherry was specifically expressed in the spermatocytes and spermatids of male and hermaphrodite germlines (Fig. 6D and Supplementary Fig. 6B, C). Further observation found that VHA-18 was localized to MOs that were probed with the MO marker SAC-1 fused with GFP in spermatocytes^25^ (Fig. 6E) and spermatids (Fig. 6F). In addition, we found that VHA-18 deletion affected GFP::NKB-2 localization at the PM, which was expressed during spermatogenesis^25^, thus suggesting that VHA-18 has an important function in the development of spermatocytes to spermatids (Supplementary Fig. 6D). It was next observed that VHA-18 loss resulted in some large and swollen MOs scattered in spermatids, unlike that in the control, where MOs were docked underneath the PM via the membrane contact site formed between the head of MO and PM, as revealed by LysoTracker Red fluorescence (Supplementary Fig. 6E, F) and TEM (Supplementary Fig. 7A). Meanwhile, vesicular morphology and acidification both appeared defective in *vha-18* spermatids, as indicated by the diffuse LysoTracker Red signal in the cytoplasmic space among the swollen MOs, which has been reported in other spe-related mutants^12,26,30,31,41^ (Supplementary Figs 6F, G). Although LysoTracker staining revealed abnormal acidification patterns, this assay cannot distinguish whether the defect results from reduced acidification or the redistribution of acidic content. Consistent with these observations, electron microscopy (EM) analyses clearly show MO swelling and structural disruption in *vha-18* spermatocytes and spermatids (Fig. 7A, B), indicating a primary defect in MO morphology. Conversely, SPE-55 loss, which affected FB formation but without influencing MOs’ structure and functions, did not alter MO acidification (Fig. 2D). Next, we wanted to determine whether the *vha-18* mutation had an effect on SPE-55::GFP. We noted a loss or reduction of SPE-55::GFP fluorescence in *vha-18* spermatocytes (Fig. 6G). Meanwhile, we also observed variation in the SPE-55::GFP signal within the germline of *vha-18;him-5* mutants, with some individuals displaying a modest reduction, whereas others revealed a significant decrease compared to the control group. We hypothesized that this discrepancy may arise from differential effects of the *vha-18* mutation across individuals. These results suggest that the MO-localized v-ATPase complex regulates MO morphology and intracellular proton homeostasis. Moreover, SPE-55::GFP localizes adjaced to the MO, and therefore, its localization or function might be affected by VHA-18 loss.

### VHA-18 is essential for spermatogenesis and fertility in *C. elegans*

We further investigated the influence of VHA-18 on spermatogenesis and first compared the ultrastructure of spermatocytes using TEM. In the control group, the FBs were semi-enclosed by the tubule-like MOs. In contrast, spermatocytes in *vha-18* mutants contained some FBs-associated vacuoles (Fig. 7A). Cryo-immune EM analysis with 1CB4^29–31,33,34,42^ revealed that these vacuoles were the swollen MOs (Fig. 7B). Moreover, during the late stage of spermatocytes in *vha-18* mutants, some FBs became naked and were no longer associated with vesicles, while other FBs remained associated with the swollen MOs but were not semi-enclosed by the tubule-like MOs as seen in the control (Fig. 7A).

Disassociation of FBs from the MOs in *spe-4, spe-5*, or *spe-10* spermatocytes affected MSP partitioning to the spermatids during the budding division^26,30,31^. Building upon these findings, we hypothesized that the *vha-18* mutant with disassociated FBs and MOs might also affect MSP partitioning after anaphase II. Indeed, an increased amount of MSP was detected in the RB of *vha-18* mutants using MSP antibody-based IFM (Fig. 7C). We also observed other defects in spermatids of the *vha-18* mutant via TEM, including the presence of intact FBs, swollen MOs, and a smaller head of MOs (Supplementary Fig. 7A), incorrect GFP::NKB-2 localization, and an abnormal chromatin mass (Supplementary Fig. 7B).

Next, we evaluated the impact of *vha-18* on sperm activation. Most of the *vha-18* mutant spermatids containing several swollen MOs failed to activate, and only a few partially activated sperm (indicated by the GFP puncta) with short pseudopods or only spiky projections in the same field could be observed (Fig. 7D). Subsequently, we assessed the fertility of the *vha-18* mutant. The *vha-18* hermaphrodite produced less than half the progeny produced by the WT (Fig. 7E). However, the reduced hermaphrodite fertility could be complemented by mating with *him-5* males (Fig. 7E), thus indicating a sperm defect in the *vha-18* hermaphrodite. To test the male fertility, control (*him-8*) or *vha-18;him-8* males were mated with *spe-8;dpy-4* hermaphrodites. Most progeny sired by the *him-8* males appeared non-dumpy (cross progeny), while most of the progeny sired by the *vha-18;him-8* males were dumpy (hermaphrodite self-progeny) (Fig. 7F), indicating that the *vha-18* male sperm were defective. Therefore, it can be concluded that VHA-18 is required for fertility in hermaphrodites and males.

## Discussion

Infertility has become a serious social problem, affecting one in six families^43^, with male factors contributing to approximately half of these cases. Casein kinase has previously been reported to play a vital role in spermatogenesis. Casein kinase 1α (CK1α) regulates spermatogenesis and male fertility via *p53-Sox3* signaling, and the conditional disruption of CK1α in spermatogonia impairs spermatogenesis and results in male infertility in mice^44^. CK2 is a cyclic-AMP and calcium-independent serine-threonine kinase that is composed of two catalytic subunits (CK2α and CK2α‘) and two regulatory β-subunits. In mice, immunoblotting studies have shown that the subunits of CK2 are primarily expressed in the testis, especially at the acrosome and mid-piece of sperm. Moreover, ablation of the CK2α’ coding gene leads to germ cell differentiation arrest and male infertility^45^.

**Fig. 8:**
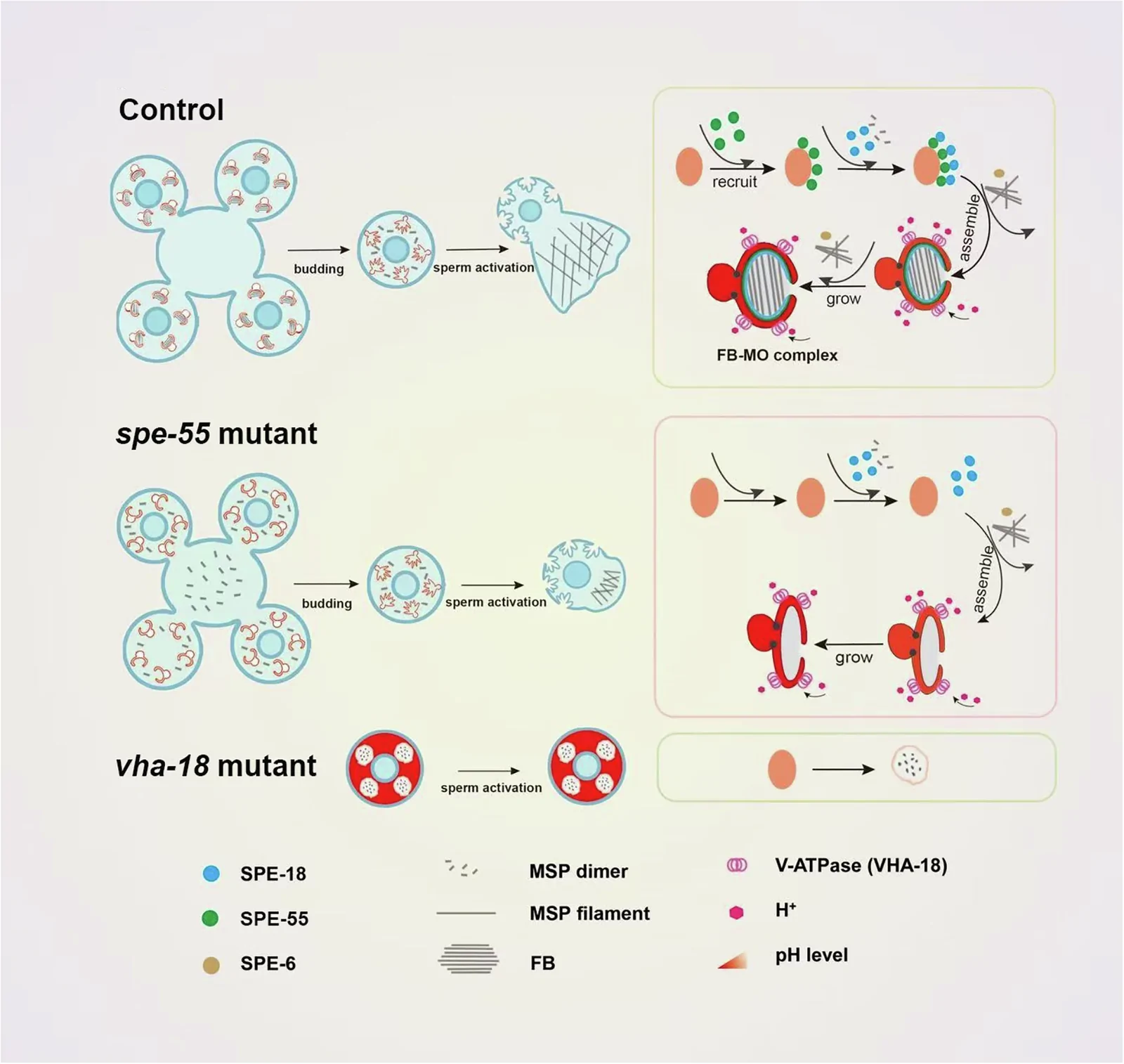
Graphical illustration of spermatogenesis in *Caenorhabditis elegans* under control, *spe-55* mutant, and *vha-18* mutant conditions. This shows the processes of budding, sperm activation, and molecular interactions, including recruitment, growth, and assembly, related to components such as SPE-18, SPE-55, SPE-6, major sperm protein (MSP) (dimer/filament), fibrous body (FB), V-ATPase (VHA-18), H⁺, and pH level changes. Under the control condition, coordinated recruitment of SPE-18, SPE-55, and SPE-6 promotes MSP dimer formation, filament elongation, and FB growth, thereby enabling proper FB–membranous organelle (MO) complex assembly and normal sperm activation. In the *spe-55* mutant, disrupted SPE-55–dependent recruitment leads to inefficient MSP polymerization and defective FB maturation despite the presence of budding and activation. In contrast, the *vha-18* mutant fails to assemble FB–MO complexes due to impaired V-ATPase activity and altered intracellular pH, which block MSP filament formation and prevent sperm activation.

Cells must precisely regulate the timing and location of cytoskeletal polymerization to ensure proper function. In nematode spermatocytes, localized MSP polymerization is believed to be crucial for preparing MSP for post-meiotic division and preventing disruption of the meiotic divisions. In this study, we identified a sperm-specific casein-like kinase, SPE-55, which is required for spermatogenesis in *C. elegans* (Fig. 1A, B). The loss of the kinase activity-dead mutation of SPE-55 results in FB formation failure in spermatocytes. The resultant spermatids contain less MSP (Fig. 3). During sperm activation, *spe-55* mutant sperm fail to fully activate and exhibit limited MO fusions and short pseudopodia, thereby causing sterility in hermaphrodites and males (Fig. 1D, E). SPE-55 is primarily expressed in the proximal arm of the male germline and exhibits a stage-specific pattern during spermatogenesis (Fig. 4B). In addition, SPE-55 localizes to the MO surface, displaying a boat-like pattern that reflects the MO’s topography during MSP filament sorting and FB expansion (Fig. 5A). SPE-55 predominantly partitions into spermatids, appearing as faint dots within the sperm cytoplasm (Supplementary Fig. 4B). Eventually, the MO head forms stable membrane contact sites with the PM, which ensures that the MOs are sorted and partitioned into developing spermatids rather than the RB during budding (Fig. 8). Therefore, we propose that the interaction between the membraneless condensate FBs and membrane-bound MOs is mutually beneficial for both structures.

In the nematode *C. elegans*, amoeboid sperm use the dynamics of the cytoskeleton composed of MSP, in place of conventional actin, to power cell motility and migration. Similar to that in mammals, each diploid spermatocyte in *C. elegans* gives rise to four haploid spermatids. Each spermatid contains minimal organelles, such as a central nucleus surrounded by some mitochondria and MOs, while cellular constituents that are unnecessary for sperm maturation and sperm–oocyte recognition are disposed of into an anucleate RB. In developing spermatocytes, several sperm-specific gene products are required to form the transient but relatively solid FBs, which are composed of bundled MSP filaments associated with the MOs. Once spermatids bud off from the RB, the FBs begin to disassemble into soluble MSP dimers. MSP constitutes 10–20% of the total protein and 40% of the soluble cellular protein in sperm. Notably, MSPs make up approximately 80% of the pseudopods’ total protein^5–8^. Thus, the interaction between membraneless condensate FBs and membrane-bound MOs in spermatocytes might facilitate the correct sorting and transport of essential constituents during spermatogenesis.

SPE-18, as an IDR-containing protein with several predicted phosphorylation sites in its C-terminal amino acid sequence, is required for FB formation and spermatogenesis^29^. SPE-55 functions upstream of SPE-18 and its kinase activity is essential for the localization of SPE-18 on the MO surface in spermatocytes. It has been confirmed in vitro that bacteria-expressed SPE-18 remains soluble at 95 °C, thus demonstrating heat resistance. Based on these findings^29^, we hypothesize that SPE-18, together with SPE-55, may promote MSP condensate assembly through phase separation. Further studies are required to elucidate the MSP dynamics mediated by SPE-55 and SPE-18 and to test this phase separation hypothesis in *C. elegans* sperm.

Meanwhile, we also observed that VHA-18, the H subunit of v-ATPase, was localized on MOs in spermatocytes, which indicates that v-ATPase may have been assembled in the spermatocytes and pumps the nearby protons into the MOs’ lumen^46^. SPE-55 functions upstream of SPE-18 and its kinase activity is essential for the localization of SPE-18 on the MO surface in spermatocytes. VHA-18 loss leads to defective FB–MO morphogenesis, resulting in naked FBs within the cytoplasm of *vha-18* spermatocytes. Consequently, this defect impairs the efficiency and/or efficacy of MSP filament sorting and partitioning into spermatids. Several proteins have been identified that influence MO morphology, including the v-ATPase B subunit SPE-5^12,26^, as well as SPE-4^30^, SPE-10^31^, and SPE-39^41^, all of which contribute to the structural organization of MOs within the spermatocytes. SPE-5 exhibits the highest degree of similarity to VHA-18. SPE-5 and VHA-18 both affect MO morphology and cause MO vacuolation beginning at the spermatocyte stage. Loss of either gene results in multinucleate spermatocytes, MSP sorting defects during the budding stage, and abnormal sperm activation. However, the severity of their phenotypes differs. The *spe-5* hermaphrodites were almost completely sterile, producing few or virtually no functional sperm, and spermatogenesis was typically arrested at the spermatocyte stage, with individual cells containing four haploid nuclei. In contrast, *vha-18* mutants rarely exhibited multinucleate (or multiple chromatin masses) spermatocytes, and hermaphrodites typically produced around 100 progenies, indicating substantially higher fertility. Furthermore, *spe-5* mutants revealed a cold-sensitive phenotype, with more severe sterility at 16 °C, a feature that has not been examined in *vha-18*. In addition, intact FBs were detected in *vha-18* spermatids (Supplementary Fig. 7A), but no such phenotype was observed in *spe-5* mutants^12,26^. Previous studies on SPE-5 relied on antibody staining to assess its expression pattern, whereas our CRISPR-Cas9–generated VHA-18::mCherry knock-in strain enabled more accurate and convenient visualization of its expression stage and pattern in vivo. Owing to its relatively high fertility, *vha-18* represents a more suitable model for investigating MO development.

During spermatogenesis, MSPs assemble into FBs in spermatocytes or the meshwork cytoskeleton in the pseudopod of spermatozoa, while MSP dimers are soluble in spermatids. Thus, MSPs exhibit three different phases, namely solid and less dynamic FBs, free and soluble dimers, and a highly motile gel-like cytoskeleton. The membrane and its immediate microenvironment might define which phase the MSPs would be in. The cargo sorting mechanism mediated by the interaction between the membraneless condensate and membrane-bound organelles demonstrated in this study might apply to intracellular biomacromolecules (such as RNA), protein trafficking, or the fine-tuning of their activities from prokaryotic to eukaryotic cells. SPE-55 functions upstream of SPE-18, and the kinase activity of SPE-55 is essential for the proper localization of SPE-18 to the MO surface. This is one of the key discoveries in this study. However, it remains unclear whether SPE-55 and SPE-18 interact directly or if other unidentified proteins are involved. Moreover, SPE-55 is a soluble protein without a transmembrane domain in its amino acid sequence. However, SPE-55::GFP exhibits a boat-like structure at the periphery of FBs, which are closely associated with the MOs. Therefore, it is plausible that SPE-55 is recruited by an unidentified protein or anchored to the MOs via certain types of modifications. Further improvement in experimental techniques will help to clarify the MSP dynamics mediated by SPE-55 in *C. elegans* sperm.

## Methods

### *C. elegans* strains and culture

Most *C. elegans* strains were maintained at 20 °C and cultured on plates containing nematode growth medium seeded with OP50 *Escherichia coli* as a food source, as previously reported^46^. Several strains used in this study were initially cultured at 16 °C and then shifted to 25 °C during the egg stage to induce temperature-sensitive phenotypes, such as *fem-1(hc17), fem-3(q96)*, and *glp-4(bn2)*. *him-5(e1490) or him-8(e1489)* was used as the control in most experiments to ensure an adequate number of males for the assays. The knock-out strains *spe-55(ibp107), spe-55(ibp116), spe-55(ibp129)*, and *spe-18(ibp158)*, as well as the knock-in strains SPE-55::GFP and SPE-18::mCherry, were generated using the CRISPR-Cas9 genome editing system. Other strains were provided by the *Caenorhabditis* Genetics Center or from our laboratory collection.

### Molecular biology techniques for transgenic strain construction

The CRISPR-Cas9–based genome editing was performed using the plasmid pDD162 carrying the target gene single guide RNA (sgRNA) sequence. The target sgRNA sequence for knock-out or knock-in was obtained from the online CRISPR design website (https://sg.idtdna.com/site/order/designtool/). Prior to inserting the target sequence into the pDD162 plasmid, the plasmid was PCR-amplified using Q5 high-fidelity DNA polymerase (New England Biolabs) with the primers listed in Supplementary Data 1. Subsequently, the PCR products were digested with *Dpn*I (New England Biolabs) for 2 h at 37 °C and transformed into DH5α competent cells.

For the construction of the *spe-55::gfp, spe-18::mCherry*, and *vha-18::mCherry* template plasmids for homologous recombination, the pPD95.75 plasmid served as the backbone and donor of the *GFP* sequence. In addition, the pCFJ150-mCherrydPiRNA plasmid (a gift from Dr Donglei Zhang’s Lab from Huazhong University of Science and Technology) was used as the donor of the mCherry sequence. The sgRNA target sequences were selected in proximity to the stop codon sequence of *spe-55, spe-18*, or *vha-18*. An approximately 2 kb fragment, encompassing segments of approximately 1 kb flanking the 5′ and 3′ ends of the target sgRNA sequence in the genome, was ligated into the GFP-removed fragment from pPD95.75. The 2 kb fragment was PCR-amplified using the N2 or *him-5* worm lysate as the template. The ligation was performed using the ClonExpress II One Step Cloning Kit (Vazyme). Subsequently, the GFP or mCherry fragment without a stop codon was PCR-amplified and ligated into the plasmid containing the 2 kb fragment immediately before the stop codon of *spe-55*, *spe-18, or vha-18*. A linker region (5′-AGCTTGCATGCCTGCAGGTCGACTCTAGAGGATCCCCGGGATT GGCCAAAGGACCCAAAGGTATGTTTCGAATGATACTAACATAACATAGAACATTTTCAGGAGGACCCTTGGAGGGTACCGGTAGAAAAA-3′) was inserted at the 5′ end of the GFP or mCherry sequences of *spe-55, spe-18, or vha-18*. Synonymous coding sequences were introduced into the sgRNA targeting sequence in the plasmid via PCR to prevent Cas9 cleavage activity. The constructed template plasmid, pDD162, which contains the target sequence, and the pRF4 plasmid, which carries the dominant Roller marker *rol-6(su1006)*, were microinjected into young adult N2 hermaphrodites at a concentration of 70 ng/µL.

To generate the point mutation that might lead to SPE-55 inactivation, we substituted the putative T195 site in the activation loop of SPE-55 with A. Similar to the process described above, a 2 kb fragment containing approximately 1 kb upstream and 1 kb downstream of the T195 locus was PCR-amplified and then ligated into the *GFP*-removed fragment from pPD95.75. Subsequently, the T195A point mutation and synonymous coding sequences were introduced into the plasmid via PCR. This template was co-injected with pRF4 and pDD162, which carries the target sequence.

### Fertility assays

A single L4-stage hermaphrodite was placed on each plate, with approximately 20 replicates per plate. The hermaphrodite was then transferred to a freshly marked plate every day until it ceased laying eggs. Thereafter, the number of progeny produced by each hermaphrodite was counted.

For the hermaphrodite fertility complemented assay, one L4 hermaphrodite and three virgin adult males were placed on a mating plate with an *E. coli* bacterial lawn and cultured for 24 h. This allowed sufficient time for the males to mate with the hermaphrodite and transfer sperm to the hermaphrodite’s uterus. Subsequently, the males were removed, and the hermaphrodite was transferred to individual fresh plates every day until no progeny was sired. The number of progeny sired by each hermaphrodite was counted.

To assess male fertility potential, we placed three 1-day post-L4 virgin males and one L4 *spe-8;dpy-4* hermaphrodite on a mating plate for 24 h. The males were removed thereafter, and the hermaphrodite was transferred to a new individual plate every day until no progeny was sired. The number of progeny with a normal or dumpy phenotype sired by each hermaphrodite was counted.

### *C. elegans* strain imaging

For hermaphrodite imaging, L4 hermaphrodites were selected and cultured for 12–16 h to reach the young adult stage. They were then placed on a 2% agarose pad and anesthetized with 0.1 mM levamisole in M9 buffer. Imaging was performed using an Olympus FV3000 confocal microscope. For male imaging, L4-stage males were selected and cultured for 48 h, followed by the same procedure as described above for the hermaphrodites.

### Sperm activation assays

L4 males were selected and cultured for 48–60 h to accumulate sufficient sperm. Subsequently, the males were dissected to release the sperm in a 3.5 μL sperm medium (SM buffer: 50 mM HEPES, 50 mM NaCl, 25 mM KCl, 1 mM MgSO_4_, 5 mM CaCl_2_, and 10 mg/mL polyvinylpyrrolidone, pH 7.8). The sperm were then perfused with various activators (200 mg/mL pronase, 1 mM ZnSO_4_, 40 mM NH_4_Cl, 30 mM TEA, or 40 nM monensin) along with membrane dyes (10 mg/mL FM 1–43; ThermoFisher Scientific) prepared in SM buffer. The images were captured using an Olympus FV1200 confocal microscope.

### Motility assay of male sperm labeled by MitoTracker Red

To assess the male-derived sperm motility after sperm activation in vivo, we cultured 2-day post-L4 virgin males overnight in a mating plate containing 50 μM MitoTracker Red (ThermoFisher Scientific). Three MitoTracker Red-labeled males and one L4 WT hermaphrodite were then placed on a normal mating plate for 8 h. Subsequently, the hermaphrodites were isolated and transferred to new plates for an additional 30 min. The hermaphrodites were then placed on a 2% agarose pad and anesthetized with 0.1 mM levamisole in M9 buffer. The position of male sperm in the hermaphrodite was observed by MitoTracker fluorescence using FV3000 confocal microscopy.

### TEM

*C. elegans* males at 48 h post-L4 stage were placed into 4 μL of SM buffer on a poly-lysine-coated Thermanox plastic coverslip and dissected to release sperm or gonad. The sperm or gonad was fixed by adding 100 μL SM buffer containing 2.5% glutaraldehyde for 2 h. The samples were then post-fixed with 1% osmium tetroxide for 2 h on ice, stained with 1% uranyl acetate overnight at 4 °C, and dehydrated with a graded series of ethanol followed by acetone transition. Thereafter, the samples were embedded in a flat mold with fresh Embed 812 resin and polymerized in an oven at 60 °C for 48 h. The coverslips were then peeled off, leaving the sample on the surface of the resin block. The trimmed block with the sample facing out was glued to a supporting blank resin block. Ultrathin sections (~80 nm) were cut using an ultramicrotome (Leica EM UC7, Germany) and observed on an FEI Tecnai Spirit 120 kV electron microscope operating at 100 kV.

### Immunofluorescence microscopy assay

Spermatids or spermatocytes dissected from virgin males at 30–48 h post-L4 stage were fixed with 4% paraformaldehyde in SM buffer overnight at 4 °C. After washing three times with phosphate-buffered saline (PBS), the sperm were permeabilized with 1% Triton ×-100 for 30 min and blocked with 2% bovine serum albumin (BSA) in PBS for 4–6 h. Subsequently, the sperm were incubated with diluted primary antibodies (MSP 1:50 or GFP 1:200; Affbiotech) overnight at 4 °C. The sperm were then washed three times with PBS and incubated with diluted Alexa 488-conjugated goat anti-mouse antibody (1:400; Invitrogen) or Alexa 546-conjugated goat anti-rabbit antibody (1:400; Invitrogen) at 25 °C for 2 h. Following another three PBS washes, the slides were sealed and analyzed using an FV3000 confocal microscope.

For the germline immunofluorescence microscopy assay, male germlines were dissected in SM buffer on a poly-lysine pad and then fixed and permeabilized with pre-cooled methanol overnight at −20 °C. After washing three times with PBS, the germlines were blocked with 0.5% BSA and 0.1% Tween-20 in M9 for 30 min and incubated with the diluted primary antibody for 2 h at room temperature. The subsequent steps were the same as those for sperm immunofluorescence.

### Cryo-immuno EM

*C. elegans* at 48 h post-L4 stage were transferred to an SM buffer on a Thermanox plastic coverslip and dissected to release the sperm or gonad. The sample was fixed with 100 μL cool PB buffer containing 4% paraformaldehyde and 0.1% glutaraldehyde at 4 °C for 2 h. Sample processing for ultrathin cryosectioning and immunolabeling was performed according to the protein A-gold method. In brief, fixed samples were embedded in 12% gelatin in 0.1 M PB at 37 °C. After solidification on ice, the gelatin block was cut into small blocks. The small blocks were then infiltrated with 2.3 M sucrose at 4 °C for two days, mounted on cryospecimen pins, and frozen in liquid nitrogen. Ultrathin cryosections (~80 nm thickness) were cut with an ultramicrotome and transferred to formvar-coated grids with 2.3 M sucrose and 2% methylcellulose. The ultrathin cryosections were immunogold-labeled and stained as previously described. In brief, the thawed sections were blocked with 100 mM glycine for 5 min and then incubated for 2 h with the primary antibody (mouse 1CB4 monoclonal antibody, 1:100) and subsequently with 10-nm colloidal gold-conjugated secondary antibody (goat anti-mouse antibody, 1:100) for 1 h. In the negative group, incubation with the primary antibody was omitted. The floating samples were fixed with a droplet of 2.5% glutaraldehyde in PBS for 5 min and collected with a wire loop with an adhering droplet of 0.2% uranyl acetate and 1.8% methylcellulose. The cryosections were observed and photographed in an FEI Tecnai Spirit 120 kV transmission electron microscope at 100 kV.

### Quantification and statistical analysis

Where *p*-values are specified, *t*-tests were performed using GraphPad Prism software. Values and definitions of *N* are provided in the text and/or figure legends. Source data are provided as a Source data file.

### Reporting summary

Further information on research design is available in the Nature Portfolio Reporting Summary linked to this article.

## Supplementary information

Supplementary Information

Description of Additional Supplementary Files

Supplementary Data 1

Supplementary Video 1

Supplementary Video 2

Supplementary Video 3

Supplementary Video 4

Reporting Summary

Transparent Peer Review file

## Data Availability

The RNA-seq data generated in this study have been deposited in the Zenodo. The DOI is: https://doi.org/10.5281/zenodo.19785628. Uncropped RT-PCR gel is available in the Source Data file. Source data are provided with this paper. Further information and requests for resources and reagents should be directed to and will be fulfilled by the corresponding author, Long Miao (lmiao@bnu.edu.cn).
