# Supplementary material for: SPE-55 and VHA-18 are essential for FB-MO complex assembly and transport during spermatogenesis in C. elegans
Source: Nat Commun. 2026 Sep 7;17:10588. doi: 10.1038/s41467-026-75577-6 (PMC13645734; doi:10.1038/s41467-026-75577-6)
Supplement: Supplementary file 1 [file 41467_2026_75577_MOESM1_ESM.pdf]

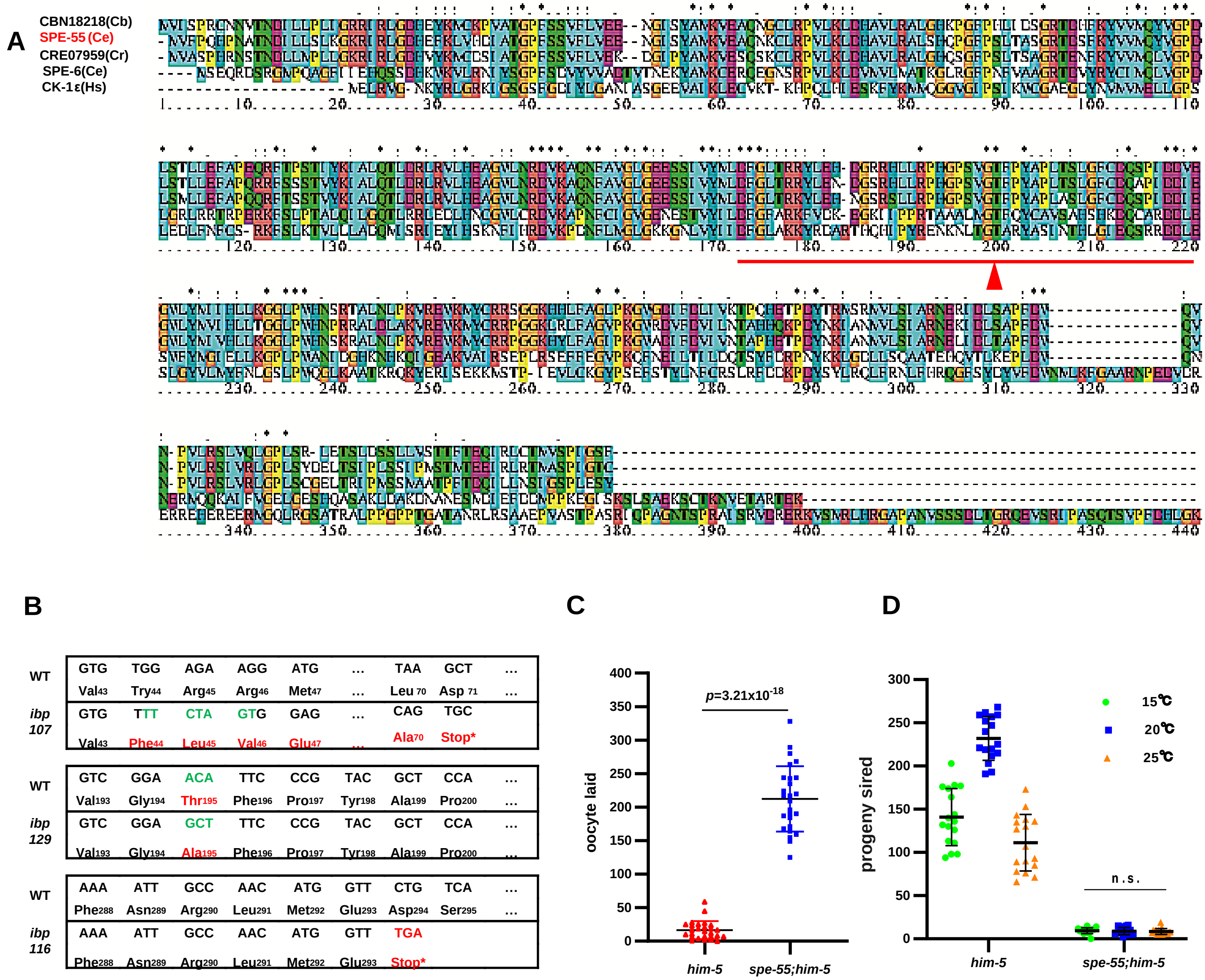

Figure S1. SPE-55 is a casein-like kinase required for spermatogenesis.

(A) Sequence alignment of the amino acids in SPE-55 and other CK1 kinases. "*" represents identical amino acids, ":" represents amino acids with high similarity, and "." represents amino acids with less similarity. Top to bottom: *Caenorhabditis brenneri* protein CBN18218, C. elegans protein SPE-55, *C. remanei* protein CRE07959, *C. elegans* protein SPE-6, and human CK1ε. The sequence starting from the DFG amino acids marked with a red line is the putative activation loop in the kinase domain. The T labeled by a red triangle is the conserved amino acid that is necessary for kinase activity in the CK1 family.

(B) The amino acid information of *spe-55* alleles. *spe-55(ibp107)* is a frameshift mutation caused by a 7 bp insertion in the first exon, resulting in a premature termination at the 71st amino acid. *spe-55(ibp129)* is a point mutation (T195A) at the conserved active site; *spe-55(ibp116)* is a C-terminus deletion allele.

(C) The number of oocytes laid by unmated hermaphrodites of the *spe-55(ibp107);him-5* and *him-5* control strains.

(D) The self-fertility of *spe-55(ibp107)* hermaphrodites was not temperature-sensitive. There was no significant difference in hermaphrodite self-fertility of *spe-55(ibp107)* at 15°C, 20°C, and 25°C. Values in (C) and (D) are presented as the mean ± standard deviation (SD). ****$p < 0.0001$. Two-sided unpaired t-test; no multiple comparison adjustment applied. n>25 hermaphrodites from three biological replicates. Source data of (C) and (D) are provided as a Source Data file.

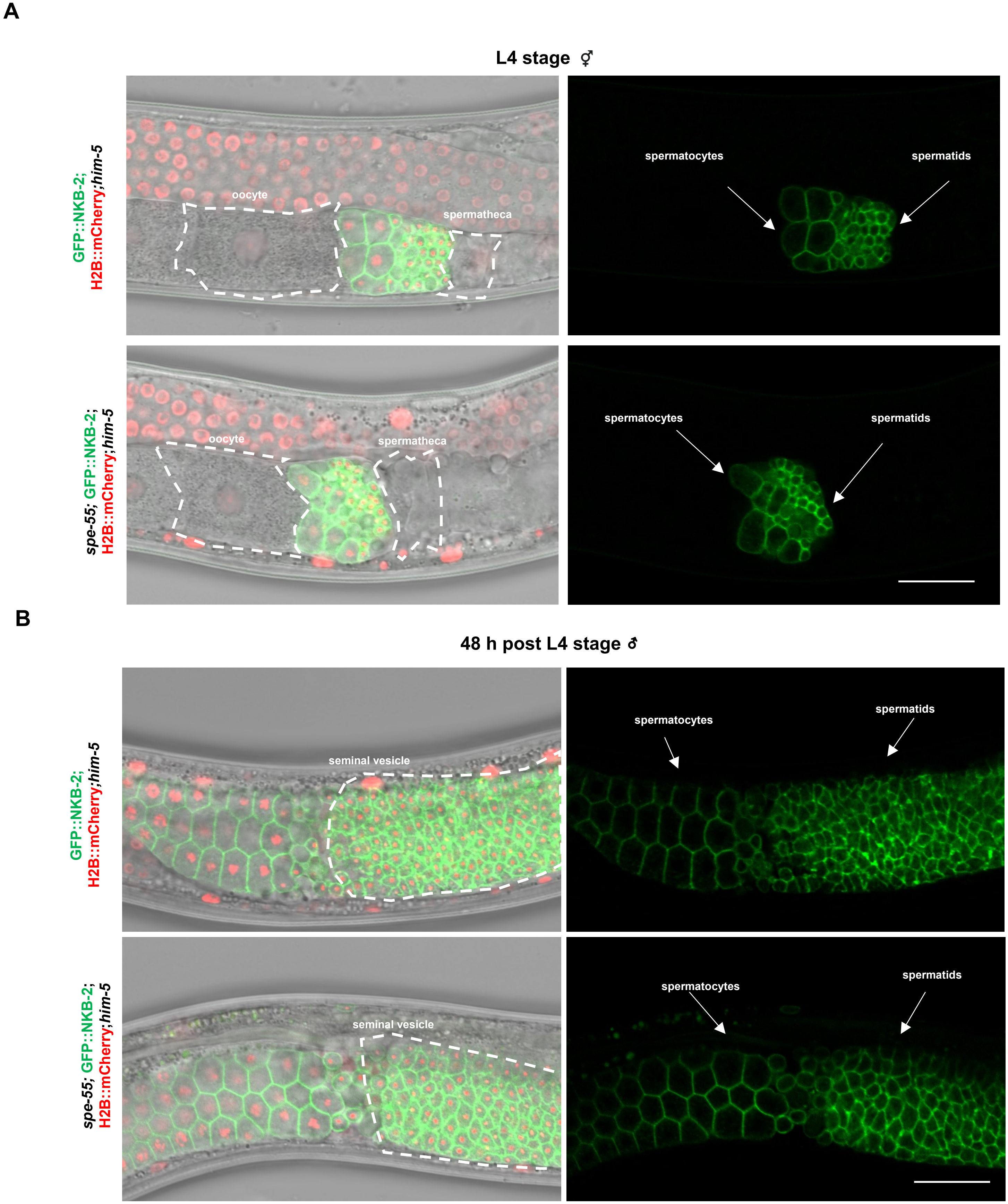

Figure S2: *spe-55* does not affect spermatogenesis.

(A) and (B) SPE-55 loss did not affect the number of spermatids produced in hermaphrodites and males. Live-cell images were captured before the first ovulation in L4 hermaphrodites (A) and at 48 h post-L4 stage males (B). GFP::NKB-2 was uniformly localized on the plasma membrane (PM) of spermatocytes and spermatids, with nuclei highlighted by H2B::mCherry fluorescence. Scale = 20 μm. Shown are representative images of three independent experiments.

A

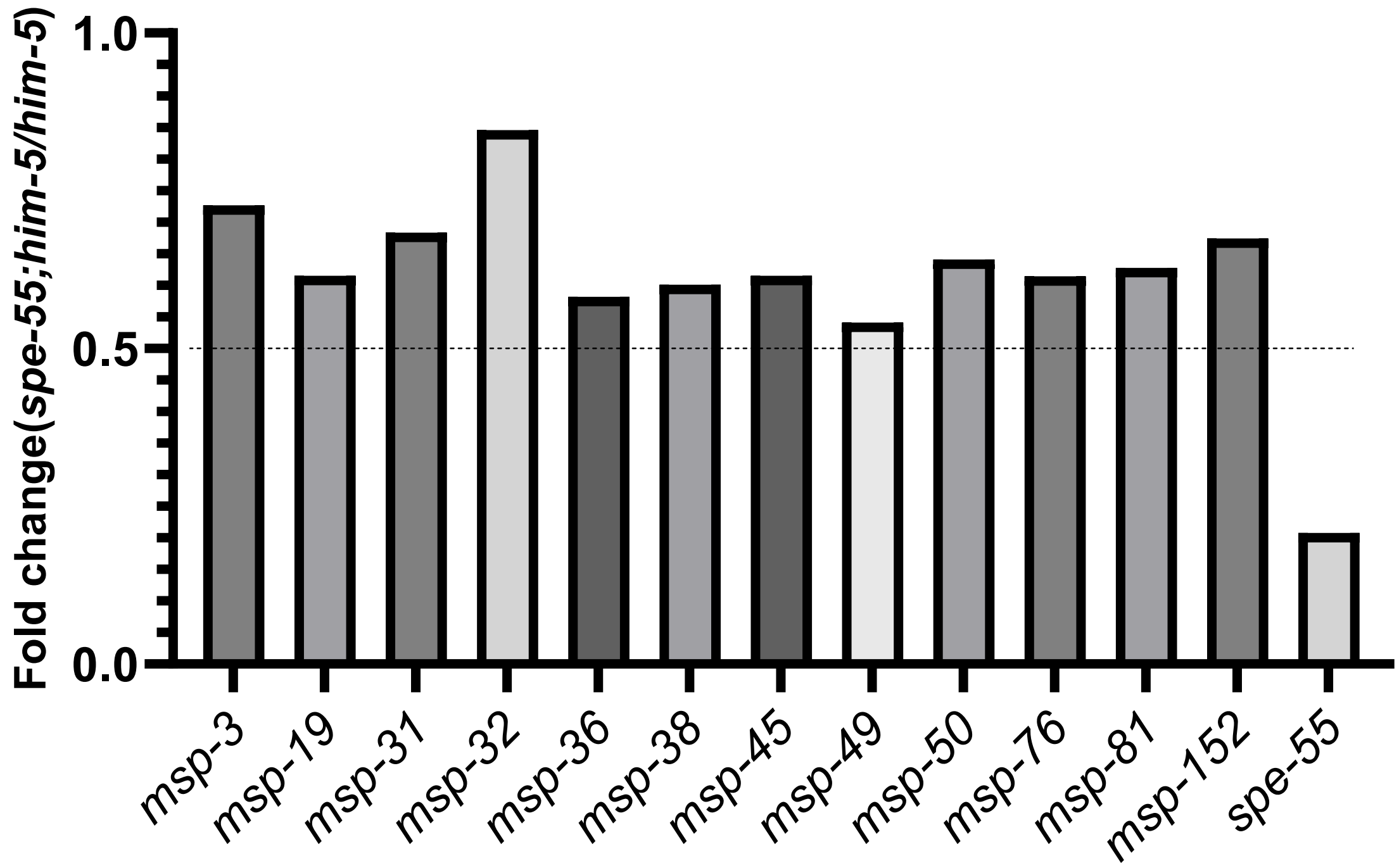

Figure S3. Loss of SPE-55 does not affect major sperm protein (MSP) mRNA levels when compared with the control.

RNA-seq analysis showed that all identified MSP mRNA levels did not change significantly in *spe-55* compared to those in the control, as determined by a significance criterion of fold change ≥2 or ≤0.5. Source data is provided as a Source Data file.

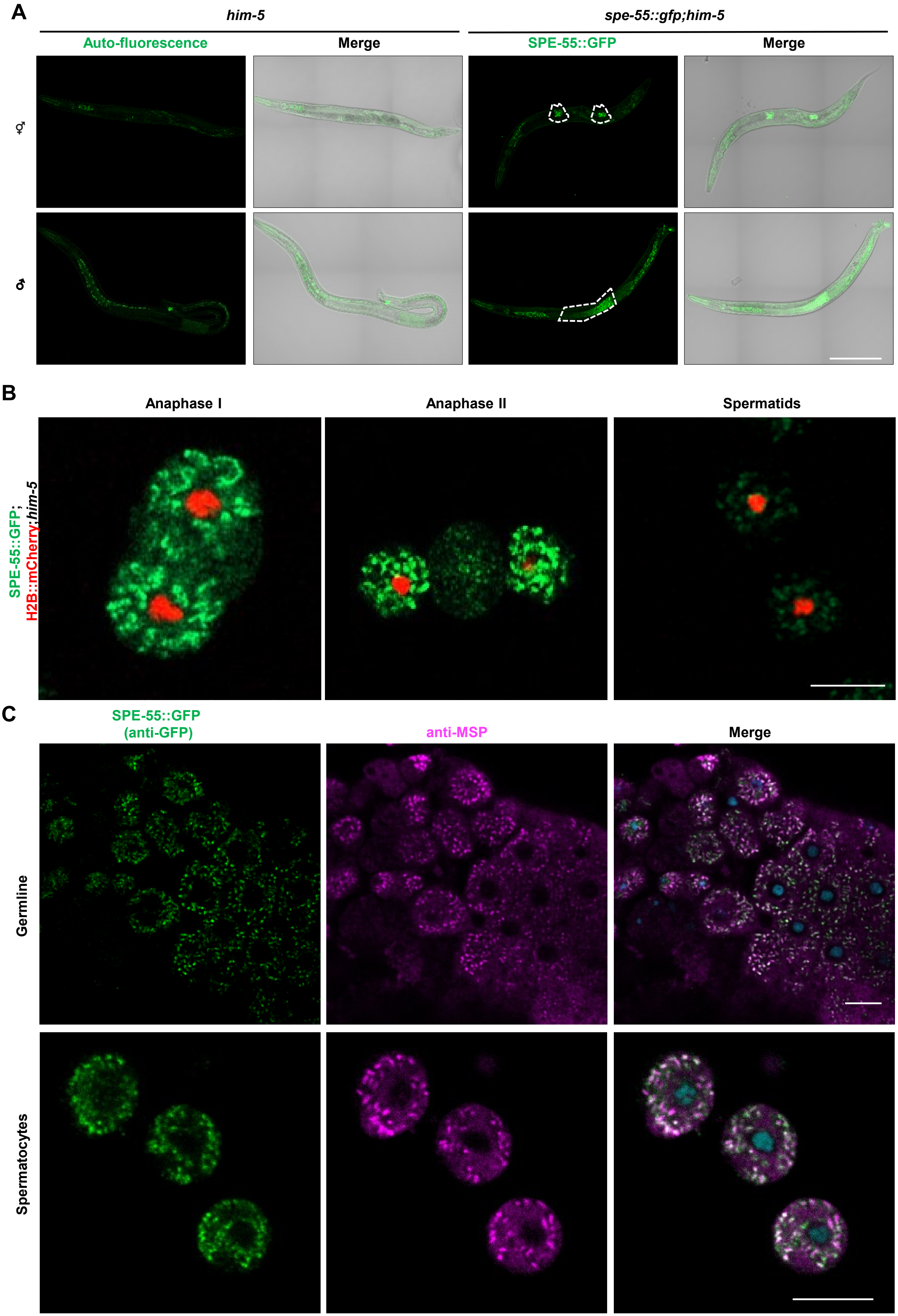
A
him-5
spe-55::gfp;him-5
Auto-fluorescence
Merge
SPE-55::GFP
Merge
⚥
♂
B
Anaphase I
Anaphase II
Spermatids
SPE-55::GFP;
H2B::mCherry;him-5
C
SPE-55::GFP
(anti-GFP)
anti-MSP
Merge
Germline
Spermatocytes

Figure S4： SPE-55 is primarily expressed in the gonad during spermatogenesis.

(A) The distribution of SPE-55::GFP in hermaphrodites and males. The autofluorescence in *him-5* males and hermaphrodites is shown in the left panels. The boxed areas indicate the distribution position of SPE-55::GFP. Scale = 20 μm. Shown are representative images of three independent experiments.
(B) Live-cell imaging of SPE-55::GFP in dividing spermatocytes and sperm. Scale = 5 μm. Shown are representative images of three independent experiments.
(C) The expression pattern of SPE-55::GFP and major sperm protein (MSP) in spermatocytes. Immunofluorescence microscopy assay showed that SPE-55 (green) was localized to the periphery of fibrous bodies (FBs) (Magenta) in meiotically dividing spermatocytes. The nuclei were labeled with DAPI. Scale bars in the upper and lower panels represent 20 μm. Shown are representative images of three independent experiments.

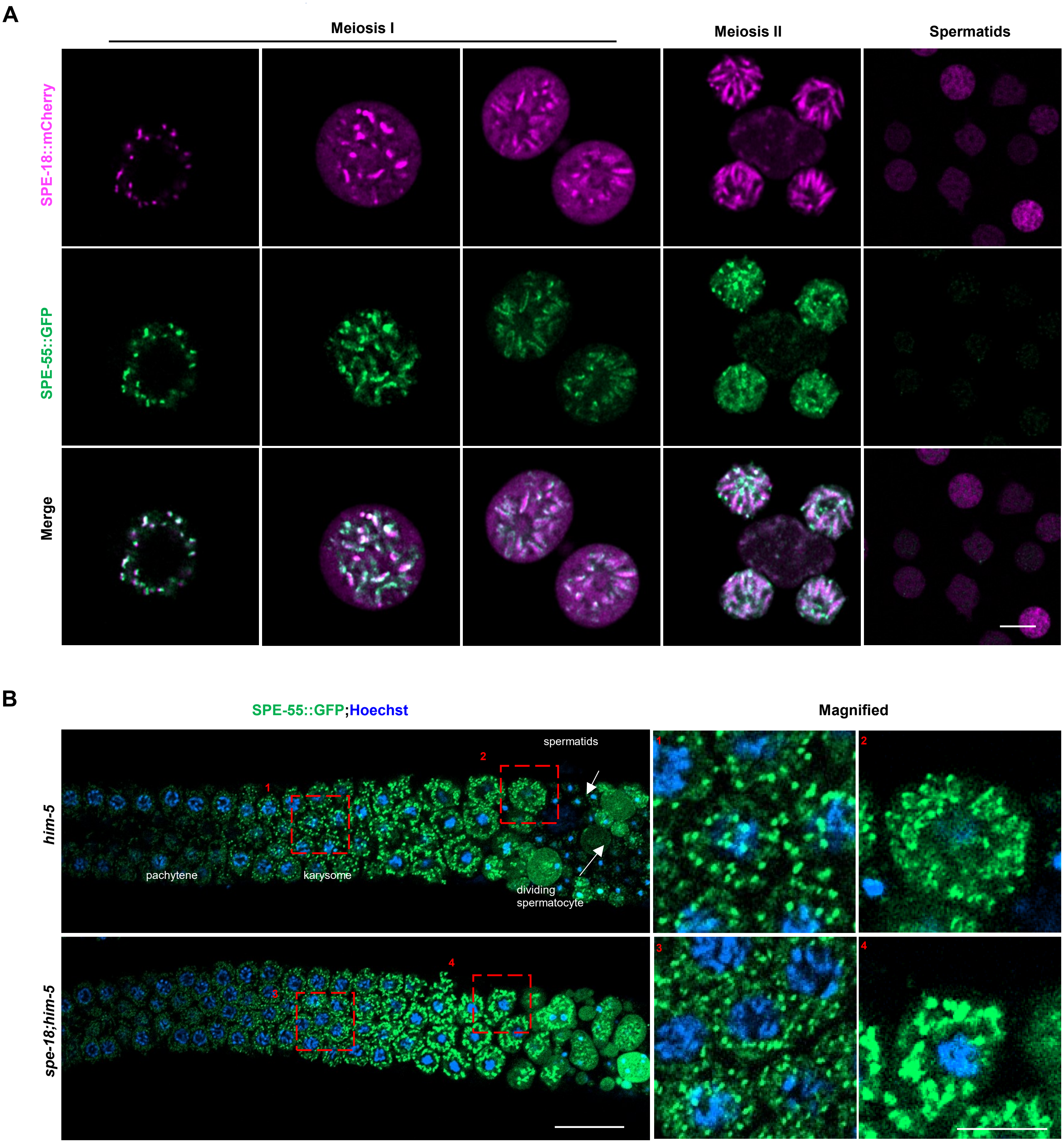

Figure S5. The localization of SPE-55 and SPE-18.

(A) SPE-18::mCherry was partially colocalized with SPE-55::GFP in spermatocytes, but not in spermatids. Scale = 5 µm. Shown are representative images of three independent experiments.
(B) Loss of SPE-18 had little to no effect on the formation of SPE-55::GFP puncta in spermatocytes. The boxed areas on the left are magnified on the right. Scale bars in the left and right panels represent 20 and 10 µm, respectively. Shown are representative images of three independent experiments.

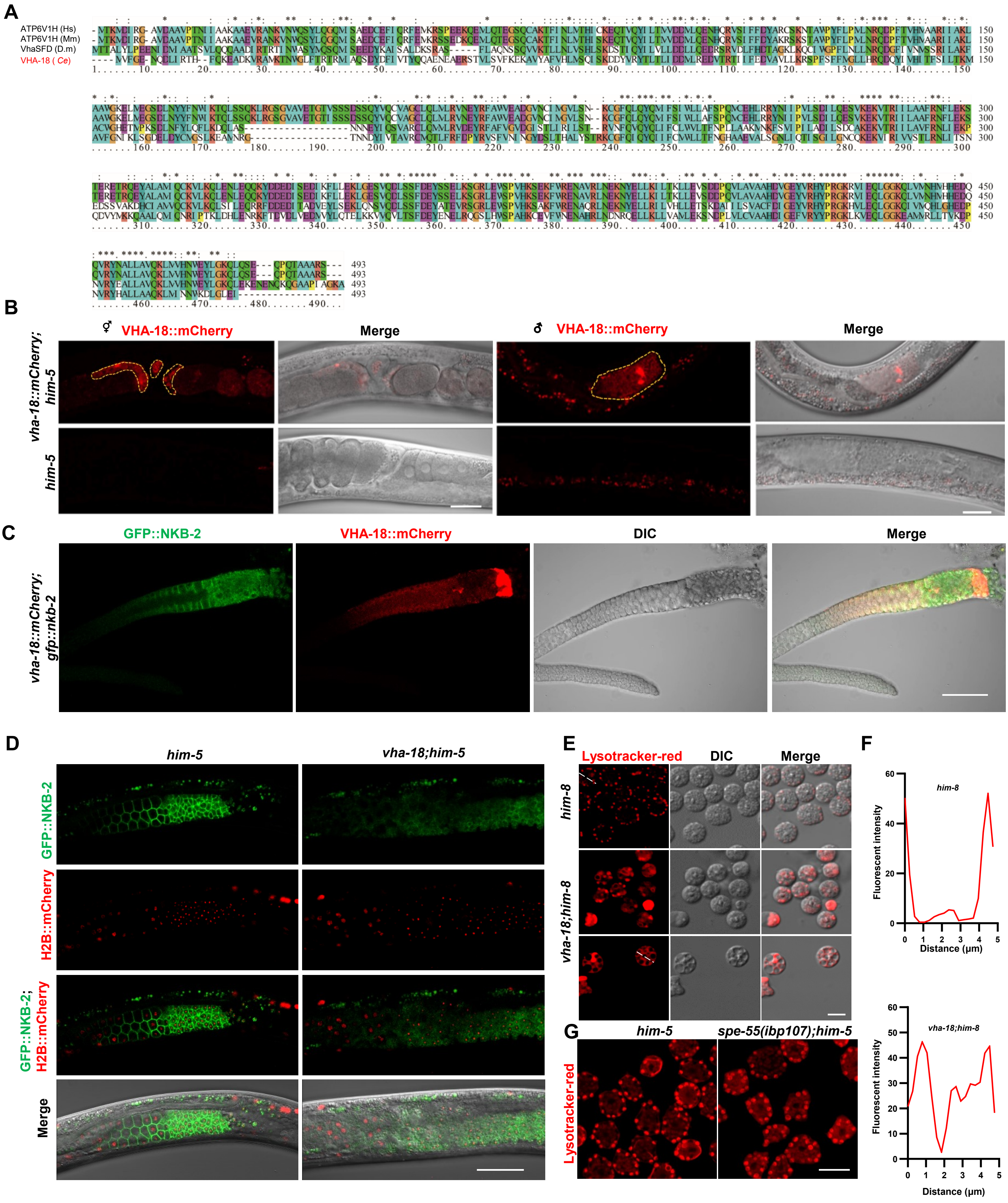
A
ATP6V1H (Hs)
ATP6V1H (Mm)
VhaSFD (D.m)
VHA-18 (Ce)
B
⚥ VHA-18::mCherry
Merge
♂ VHA-18::mCherry
Merge
vha-18::mCherry; him-5
him-5
C
GFP::NKB-2
VHA-18::mCherry
DIC
Merge
vha-18::mCherry; gfp::nkb-2
D
him-5
vha-18;him-5
GFP::NKB-2
H2B::mCherry
GFP::NKB-2; H2B::mCherry
Merge
E
Lysotracker-red
DIC
Merge
him-8
vha-18;him-8
F
him-8
Fluorescent intensity
Distance (μm)
vha-18;him-8
Fluorescent intensity
Distance (μm)
G
him-5
spe-55(ibp107);him-5
Lysotracker-red

Figure S6. Expression pattern of VHA-18.

(A) Sequence alignment of the amino acids of VHA-18 and v-ATP H subunits in other species. From top to bottom: *Homo sapiens* ATP6V1H, *Mus musculus* ATP6V1H, *Drosophila melanogaster* VhaSFD, and *Caenorhabditis elegans* VHA-18.
(B) VHA-18::mCherry was expressed in the regions where spermatogenesis occurs in both hermaphrodites and males. The distribution of VHA-18::mCherry is indicated by yellow dashed lines. The bottom panels are the autofluorescence in control hermaphrodites and males. Scale = 20 μm. Shown are representative images of three independent experiments.
(C) VHA-18::mCherry was primarily expressed in the proximal terminal of the male gonad, as indicated by the expression of GFP::NKB-2. Scale = 20 μm. Shown are representative images of three independent experiments.
(D) VHA-18 loss affects spermatogenesis and the localization of GFP::NKB-2. The live-cell image was captured from males that were 24 h post-L4 stage. GFP::NKB-2 was uniformly localized on the plasma membrane (PM) of spermatocytes and spermatids, with their nuclei highlighted by H2B::mCherry fluorescence. Scale = 20 μm. Shown are representative images of three independent experiments.
(E) LysoTracker Red staining of dissected *vha-18;him-8* spermatids showed that some LysoTracker Red-stained structures were larger and scattered within the cell (middle), whereas in another case, LysoTracker Red only stained the outer spaces between the MOs (bottom). This pattern differs from the control *him-8*, where LysoTracker Red-labeled puncta (MOs) are located just beneath the PM (top). Scale = 5 μm. Shown are representative images of three independent experiments.
(F) Two graphs of *him-8*(upper) and *vha-18;him-8*(lower) indicates the quantification of the fluorescence intensity in the dashed lines of *him-8* and *vha-18;him-8* group shown in (E). Source data is provided as a Source Data file.
(G) SPE-55 loss does not affect the morphology or acidification of MOs in spermatids. LysoTracker staining shows no difference between the spermatids of *him-5* and *spe-55;him-5*. Scale = 5 μm. Shown are representative images of three independent experiments.

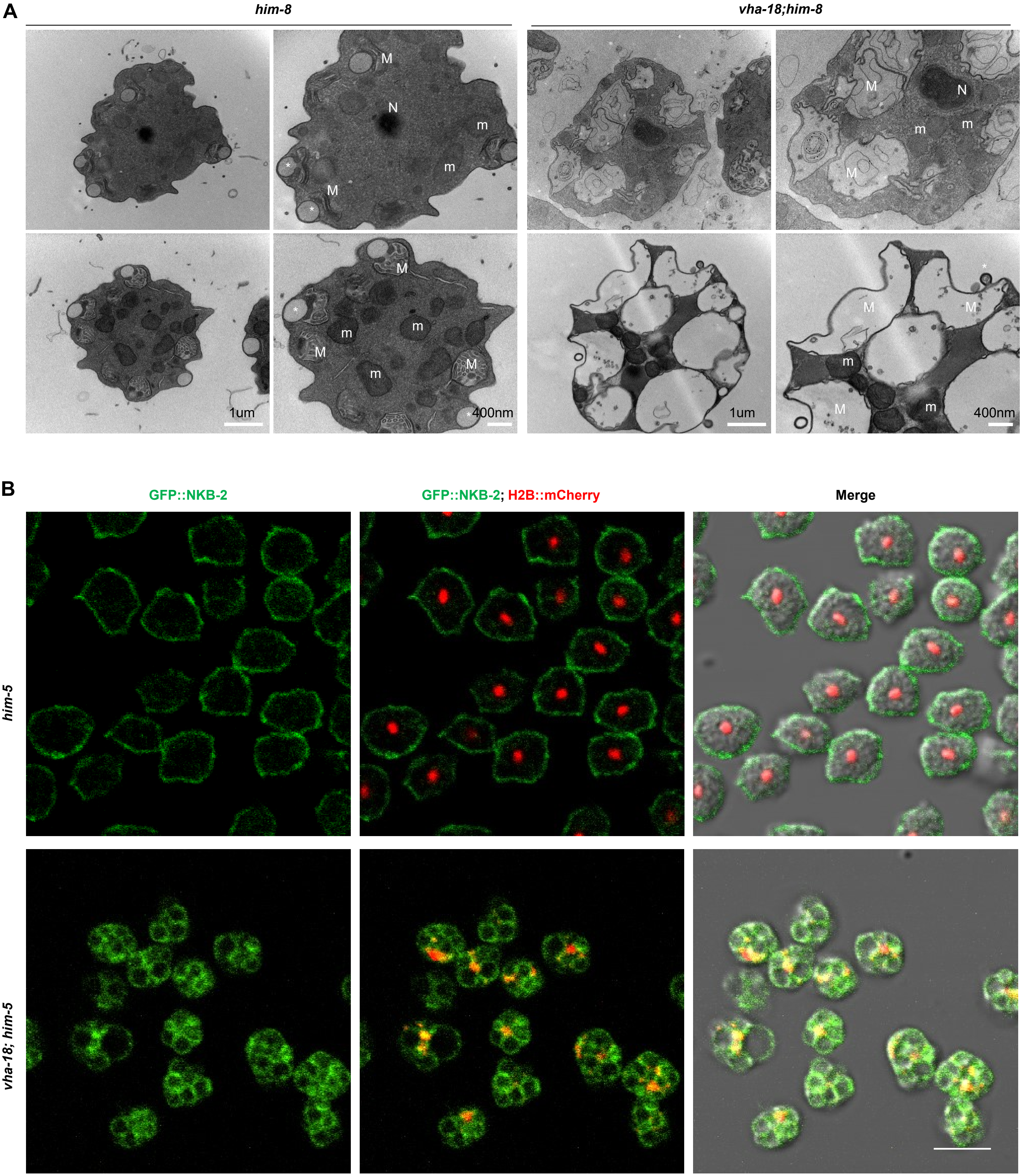
A
him-8
vha-18;him-8
M
N
m
m
M
*
*
M
m
m
M
M
m
1um
400nm
M
N
m
m
M
M
M
m
m
M
*
1um
400nm
B
GFP::NKB-2
GFP::NKB-2; H2B::mCherry
Merge
him-5
vha-18; him-5

Figure S7. VHA-18 is essential for fibrous body (FB)-membranous organelle (MO) morphogenesis and protein sorting.

(A) Electron microscopy (EM) images of spermatids reveal multiple morphological defects in *vha-18;him-8* mutant spermatids. The upper row shows irregular and disordered vesicles within the spermatids. The lower row displays intact FBs, swollen MOs, and MOs with notably smaller heads. Labels: “N” indicates nucleus; “M” indicates MO; “m” indicates mitochondria; “*” marks the MO’s head. The blue arrow indicates an intact FB structure. Scale: left column, 1 μm; right column, 400 nm. The images correspond to wild-type *him-8* (left) and mutant *vha-18;him-8* (right), respectively. Shown are representative images of three independent experiments.
(B) Live-cell imaging of spermatids from *vha-18;gfp::nkb-2;H2B::mCherry* shows numerous GFP-positive vesicles accompanied by abnormal mCherry-labeled nuclei. In contrast, the control spermatids display evenly localized GFP on the plasma membrane (PM) without internal organelle labeling. Scale = 5 μm. Shown are representative images of three independent experiments.
