# Supplementary material for: SPE-55 and VHA-18 are essential for FB-MO complex assembly and transport during spermatogenesis in C. elegans
Source: Nat Commun. 2026 Sep 7;17:10588. doi: 10.1038/s41467-026-75577-6 (PMC13645734; doi:10.1038/s41467-026-75577-6)
Supplement: Supplementary file 2 [file 41467_2026_75577_MOESM2_ESM.pdf]

Description of Additional Supplementary Files

Supplementary Video 1. Time-lapse analysis shows that ectopically activated spermatozoa with punctate GFP::NKB-2 fluorescence are highly motile, actively moving around within the seminal vesicle of the control swm-1;him-5 males (related to Fig. 2B). N = 5 worms measured in this assay. Scale = 10 µm

Supplementary Video 2. Time-lapse analysis shows that ectopically yet incompletely activated sperm with polarized GFP::NKB-2 puncta remain immobile within the seminal vesicle of spe-55;swm-1;him-5 males (related to Fig. 2B). N = 5 worms measured in this assay. Scale = 10 µm with

Supplementary Video 3. Time-lapse analysis shows that male sperm GFP::NKB-2; H2B::mCherry fluorescence predominantly accumulated within the spermatheca of feminized fog-2 worms (related to Fig. 2B and Fig. 1F). N = 5 fog-2 worms measured in this assay. Scale = 50 µm

Supplementary Video 4. Time-lapse analysis shows that sperm from spe-55 mutant males rarely accumulated in the spermatheca and instead remained dispersed throughout the uterus of feminized fog-2 worms (related to Fig. 2B and Fig. 1F). N = 5 fog-2 worms measured in this assay. Scale = 50 µm

Supplementary Data 1 - Supplementary Data 1 contains information about reagents, primers, plasmids and strains.
