# Supplementary material for: SPE-55 and VHA-18 are essential for FB-MO complex assembly and transport during spermatogenesis in C. elegans
Source: Nat Commun. 2026 Sep 7;17:10588. doi: 10.1038/s41467-026-75577-6 (PMC13645734; doi:10.1038/s41467-026-75577-6)
Supplement: Supplementary file 3 [file 41467_2026_75577_MOESM8_ESM.pdf]

nature portfolio

Corresponding author(s): Long Miao, Yanmei Zhao,Yong Liu, Peng Wang

Last updated by author(s): April 25, 2026

Reporting Summary

Nature Portfolio wishes to improve the reproducibility of the work that we publish. This form provides structure for consistency and transparency in reporting. For further information on Nature Portfolio policies, see our Editorial Policies and the Editorial Policy Checklist.

Statistics

For all statistical analyses, confirm that the following items are present in the figure legend, table legend, main text, or Methods section.

n/a	Confirmed	
☐	☒	The exact sample size (*n*) for each experimental group/condition, given as a discrete number and unit of measurement
☐	☒	A statement on whether measurements were taken from distinct samples or whether the same sample was measured repeatedly
☐	☒	The statistical test(s) used AND whether they are one- or two-sided<br>*Only common tests should be described solely by name; describe more complex techniques in the Methods section.*
☒	☐	A description of all covariates tested
☐	☒	A description of any assumptions or corrections, such as tests of normality and adjustment for multiple comparisons
☐	☒	A full description of the statistical parameters including central tendency (e.g. means) or other basic estimates (e.g. regression coefficient) AND variation (e.g. standard deviation) or associated estimates of uncertainty (e.g. confidence intervals)
☐	☒	For null hypothesis testing, the test statistic (e.g. *F*, *t*, *r*) with confidence intervals, effect sizes, degrees of freedom and *P* value noted<br>*Give P values as exact values whenever suitable.*
☒	☐	For Bayesian analysis, information on the choice of priors and Markov chain Monte Carlo settings
☒	☐	For hierarchical and complex designs, identification of the appropriate level for tests and full reporting of outcomes
☒	☐	Estimates of effect sizes (e.g. Cohen's *d*, Pearson's *r*), indicating how they were calculated

Our web collection on statistics for biologists contains articles on many of the points above.

Software and code

Policy information about availability of computer code

Data collection	Microscope: Olympus FV1200; Olympus FV3000; Zeiss LSM 980; FEI Tecnai Spirit 120 electron microscope; Ultramicrotome (Leica EM UC7)
Data analysis	ImageJ (Fiji),GraphPad Prism 5, Microsoft Excel

For manuscripts utilizing custom algorithms or software that are central to the research but not yet described in published literature, software must be made available to editors and reviewers. We strongly encourage code deposition in a community repository (e.g. GitHub). See the Nature Portfolio guidelines for submitting code & software for further information.

Data

Policy information about availability of data

All manuscripts must include a data availability statement. This statement should provide the following information, where applicable:

- Accession codes, unique identifiers, or web links for publicly available datasets
- A description of any restrictions on data availability
- For clinical datasets or third party data, please ensure that the statement adheres to our policy

This study did not generate unique code. The RNA-seq data generated in this study have been deposited in XXX under Number XXX. All data supporting the findings of the study are available within the main manuscript and its supplementary information. Caenorhabditis elegans strains, plasmids and oligonucleotides information used in the paper are listed in Supplementary Table.

Research involving human participants, their data, or biological material

Policy information about studies with human participants or human data. See also policy information about sex, gender (identity/presentation), and sexual orientation and race, ethnicity and racism.

Reporting on sex and gender	This study does not involve human participants.
Reporting on race, ethnicity, or other socially relevant groupings	This study does not involve human participants.
Population characteristics	This study does not involve human participants.
Recruitment	This study does not involve human participants.
Ethics oversight	This study does not involve human participants.

Note that full information on the approval of the study protocol must also be provided in the manuscript.

Field-specific reporting

Please select the one below that is the best fit for your research. If you are not sure, read the appropriate sections before making your selection.

☒ Life sciences ☐ Behavioural & social sciences ☐ Ecological, evolutionary & environmental sciences

For a reference copy of the document with all sections, see nature.com/documents/nr-reporting-summary-flat.pdf

Life sciences study design

All studies must disclose on these points even when the disclosure is negative.

Sample size	The information of related sample size has been shown in the figure legend or method of main text. For fertility assays, number of worms in each group is over 20; For motility assay of male sperm labeled by MitoTracker Red, 6 worms in each group were measured; For worm imaging collection, n=5; For sperm imaging, more than 10 worms were dissected to obtain sperm. For electron microscopy, more than 30 worms were dissected to obtain sperm.
Data exclusions	No experimental data was excluded.
Replication	Experiments were independently repeated two or more times, and all attempts at replication were successful.
Randomization	Samples were allocated randomly into experimental groups without bais.
Blinding	All experiment were blinded and did not involve bias during data collection.

Behavioural & social sciences study design

All studies must disclose on these points even when the disclosure is negative.

Study description	
Research sample	
Sampling strategy	
Data collection	
Timing	
Data exclusions	
Non-participation	
Randomization	

Ecological, evolutionary & environmental sciences study design

All studies must disclose on these points even when the disclosure is negative.

Study description	
Research sample	
Sampling strategy	
Data collection	
Timing and spatial scale	
Data exclusions	
Reproducibility	
Randomization	
Blinding	

Did the study involve field work? ☐ Yes ☐ No

Field work, collection and transport

Field conditions	
Location	
Access & import/export	
Disturbance	

Reporting for specific materials, systems and methods

We require information from authors about some types of materials, experimental systems and methods used in many studies. Here, indicate whether each material, system or method listed is relevant to your study. If you are not sure if a list item applies to your research, read the appropriate section before selecting a response.

Materials & experimental systems

n/a	Involved in the study	
☐	☒	Antibodies
☒	☐	Eukaryotic cell lines
☒	☐	Palaeontology and archaeology
☐	☒	Animals and other organisms
☒	☐	Clinical data
☒	☐	Dual use research of concern
☒	☐	Plants

Methods

n/a	Involved in the study	
☒	☐	ChIP-seq
☒	☐	Flow cytometry
☒	☐	MRI-based neuroimaging

Antibodies

Antibodies used	1. MSP monoclonal antibody 4A5 (DSHB; Cat#4A5; RRID:AB_760348); Dilution: 1:50.<br>2.GFP-Tag Rabbit Polyclonal Antibody (Affinity Biosciences; Cat#T0006, RRID::AB_2839423); Dilution: 1:200.<br>2. Goat anti-Rabbit IgG (H+L) CrossAdsorbed Secondary Antibody (Invitrogen; Cat#A-11008; RRID: AB_143165); Dilution: 1:400.<br>3. Goat anti-Mouse IgG (H+L) CrossAdsorbed Secondary Antibody (Invitrogen; Cat#A-11003; RRID:AB_2206621); Dilution: 1:400.<br>4. Goat anti-Mouse IgG (H+L) CrossAdsorbed Secondary Antibody (Invitrogen; Cat#A-21422; RRID: AB_2535844); Dilution: 1:400.
Validation	1. MSP monoclonal antibody (DSHB; Cat#4A5; RRID:AB_760348): Manufacturer validated for immunofluorescent (IF); And validated by MSP detection, see Fig3A-3D; Fig4C,4D; Fig7C.

2. Goat anti-Rabbit IgG (H+L) CrossAdsorbed Secondary Antibody (Invitrogen; Cat#A-11008; RRID: AB_143165) and Goat anti-Mouse IgG (H+L) CrossAdsorbed Secondary Antibody (Invitrogen; Cat#A-11003; RRID:AB_2206621) and Goat anti-Mouse IgG (H+L) CrossAdsorbed Secondary Antibody (Invitrogen; Cat#A-21422; RRID: AB_2535844): Manufacturer validated for IF, see Fig3A-3D; Fig4C,4D;4E; Fig7C; FigS4C.
3. GFP-Tag Rabbit Polyclonal Antibody (Affinity Biosciences; Cat#T006, RRID: AB_2839423): Manufacturer validated for IF, see Fig 4E; FigS4C.

Eukaryotic cell lines

Policy information about cell lines and Sex and Gender in Research

Cell line source(s)

Authentication

Mycoplasma contamination

Commonly misidentified lines
(See ICLAC register)

Palaeontology and Archaeology

Specimen provenance

Specimen deposition

Dating methods

☐ Tick this box to confirm that the raw and calibrated dates are available in the paper or in Supplementary Information.

Ethics oversight

Note that full information on the approval of the study protocol must also be provided in the manuscript.

Animals and other research organisms

Policy information about studies involving animals; ARRIVE guidelines recommended for reporting animal research, and Sex and Gender in Research

Laboratory animals: Caenorhabditis elegans - N2 Bristol strain, him-5(e1490) and him-8(e1489) were used as control animals. Both hermaphrodites and male animals were used in this study. Animals' stage was indicated at corresponding figure legends and methods. All strains used in the study and their sources are listed in Supplementary Table.

Wild animals: This study did not involve wild animals.

Reporting on sex: There are hermaphrodites and males in Caenorhabditis elegans. And both hermaphrodites and male animals were used to study the process of spermatogenesis.

Field-collected samples: Strains were cultured on the standard NGM agar plates at 20°C, and using Escherichia coli (E. coli) OP50 as food source.

Ethics oversight: All animals are Caenorhabditis elegans, therefore no ethical approval was required in this study.

Note that full information on the approval of the study protocol must also be provided in the manuscript.

Clinical data

Policy information about clinical studies
All manuscripts should comply with the ICMJE guidelines for publication of clinical research and a completed CONSORT checklist must be included with all submissions.

Clinical trial registration

Study protocol

Data collection

Outcomes

Dual use research of concern

Policy information about dual use research of concern

Hazards

Could the accidental, deliberate or reckless misuse of agents or technologies generated in the work, or the application of information presented in the manuscript, pose a threat to:

No	Yes	
☐	☐	Public health
☐	☐	National security
☐	☐	Crops and/or livestock
☐	☐	Ecosystems
☐	☐	Any other significant area

Experiments of concern

Does the work involve any of these experiments of concern:

No	Yes	
☐	☐	Demonstrate how to render a vaccine ineffective
☐	☐	Confer resistance to therapeutically useful antibiotics or antiviral agents
☐	☐	Enhance the virulence of a pathogen or render a nonpathogen virulent
☐	☐	Increase transmissibility of a pathogen
☐	☐	Alter the host range of a pathogen
☐	☐	Enable evasion of diagnostic/detection modalities
☐	☐	Enable the weaponization of a biological agent or toxin
☐	☐	Any other potentially harmful combination of experiments and agents

Plants

Seed stocks	Plant samples were not involved in this study.
Novel plant genotypes	Plant samples were not involved in this study.
Authentication	Plant samples were not involved in this study.

ChIP-seq

Data deposition

☐ Confirm that both raw and final processed data have been deposited in a public database such as GEO.

☐ Confirm that you have deposited or provided access to graph files (e.g. BED files) for the called peaks.

Data access links<br>*May remain private before publication.*	
Files in database submission	
Genome browser session<br>(e.g. UCSC)	

Methodology

Replicates	

Sequencing depth

Antibodies

Peak calling parameters

Data quality

Software

Flow Cytometry

Plots

Confirm that:

- [ ] The axis labels state the marker and fluorochrome used (e.g. CD4-FITC).
- [ ] The axis scales are clearly visible. Include numbers along axes only for bottom left plot of group (a 'group' is an analysis of identical markers).
- [ ] All plots are contour plots with outliers or pseudocolor plots.
- [ ] A numerical value for number of cells or percentage (with statistics) is provided.

Methodology

Sample preparation

Instrument

Software

Cell population abundance

Gating strategy

- [ ] Tick this box to confirm that a figure exemplifying the gating strategy is provided in the Supplementary Information.

Magnetic resonance imaging

Experimental design

Design type

Design specifications

Behavioral performance measures

Acquisition

Imaging type(s)

Field strength

Sequence & imaging parameters

Area of acquisition

Diffusion MRI ☐ Used ☐ Not used

Preprocessing

Preprocessing software

Normalization

Normalization template

Noise and artifact removal

Volume censoring

Statistical modeling & inference

Model type and settings

Effect(s) tested

Specify type of analysis: ☐ Whole brain ☐ ROI-based ☐ Both

Statistic type for inference

(See Eklund et al. 2016)

Correction

Models & analysis

n/a	Involved in the study
☐	☐ Functional and/or effective connectivity
☐	☐ Graph analysis
☐	☐ Multivariate modeling or predictive analysis

Functional and/or effective connectivity

Graph analysis

Multivariate modeling and predictive analysis
