# Supplementary material for: SPE-55 and VHA-18 are essential for FB-MO complex assembly and transport during spermatogenesis in C. elegans
Source: Nat Commun. 2026 Sep 7;17:10588. doi: 10.1038/s41467-026-75577-6 (PMC13645734; doi:10.1038/s41467-026-75577-6)
Supplement: Supplementary file 4 [file 41467_2026_75577_MOESM9_ESM.pdf]

nature portfolio

Peer Review File

SPE-55 and VHA-18 are essential for FB-MO complex assembly and transport during spermatogenesis in C. elegans

Corresponding Author: Dr Peng Wang

This file contains all reviewer reports in order by version, followed by all author rebuttals in order by version.

Version 0:

Reviewer comments:

Reviewer #1

(Remarks to the Author)
In nematode spermatocytes, localized assembly of the major sperm protein (MSP) into fibrous bodies is critical for efficient post-meiotic partitioning of this key motility protein to the haploid sperm. We are only just beginning to understand how these fibrous bodies assemble on the cytosolic side of Golgi-derived membranous organelles. Previous work identified involvement of an intrinsically disordered protein SPE-18, this study by Ma et al greatly expands our understanding of the assembly process by identifying specific role for the CK1 related kinase (SMNK-1) in the fibrous body assembly process – which occurs in meiotic prophase spermatocytes. In addition, they show a role for the v-ATPase in MO integrity in meiotic stage and budding figure spermatocytes. The greater significance of this work is that it highlights an experimentally accessible example of how cytoskeletal elements can assemble adjacent to a biological membrane with distinct contributions of a regulatory kinase and intrinsically disordered protein as well as role for a localized alkaline microenvironment during later stages of the process. This work should be of interest to a variety of readers from those interested in gametogenesis to those interested in liquid condensate mediated complex assembly. There is a lot of nice work in this paper but there are issues in the text and documentation of the experiments that need to be addressed.

The title, the abstract (line 28 “meanwhile”) and the end of the introduction (Line 89-92) are a bit misleading as they could easily give the impression that the alkaline environment is critical for the initial assembly of fibrous bodies in the meiotic prophase spermatocytes. The actual data shows a role for VHA-8 in later meiotic stage spermatocytes (MO integrity) and post-anaphase II budding figures (efficient MSP segregation). Thus VHA-8 may be more important in maintaining the integrity of the MOs and either directly or indirectly the already formed or final growth stages of the FBs. The authors should clarify these distinctions in these instances and throughout the paper. SMNK-1 is important for the initial assembly of the FB-MO complex assembly (especially the FB) whereas VHA-8 may be more important of maintaining the complex (especially the MO) once the FB-MO complex has already formed.

Naming of the C09B9.4 gene. Gene names in C. elegans must be approved in advanced. It is unclear why C09B9.4 has not been given a Spe (spermatogenesis defective) designation since it clearly fits within this class of genes. Has the name smnk-1 been approved by S. L’Hernault and/or Tim Schedl?

In supplementary figure S1A, there is a problem with the amino acid numbering as the aligned proteins are not all the same length and the arrow that is supposed to indicate a critical conserved Thr is pointing to a glycine. Fix in the figure and edit main text appropriately.

Paragraph 149-154 should be moved up – before the section on the spe-8 crosses.

Line. 148 – the conclusion from the spe-8 crosses should be that 1) the males have a fertility severe fertility defect, 2) their seminal fluid contains functional sperm activator, and 3) the smnk-1 male sperm does not out-compete the spe-8 hermaphrodite sperm.

In Figure 2A, it would be helpful to include H2B in the enlarged images of the spermatozoa to give a better sense of how many sperm are being shown in the mutant image.

Line 186 – change “apparent” to “immediately apparent” since the analysis of potential germline defects is limited. Authors could note or quantify if a normal number of spermatids are produced.

Videos of sperm within swm-1 males is a somewhat artificial environment. However, this result is paired with other lines of evidence; so, it is ok. A better experiment would be to video the GFP::NKB-2 male sperm (+/- the smnk-1 mutation) within the uterus of feminized non-smnk-1 worms.

The studies documenting the defects in MO fusion but not MO numbers are nice! It is worth noting that fewer MOs fuse in smnk-1 sperm – but that their data suggest that all sperm respond to activation by fusing at least some MOs (even if the sperm aren’t extending pseudopods). The authors should make the point that all sperm are responding to activation – albeit weakly and with defects.

Documenting the failure of MSP to assemble into FBs with both anti-MSP immunofluorescence and TEM is really nice. The images for Fig3F are very nice. Interpretation of the budding figure (Fig3G) is less clear. It’s hard to tell if that is an MO material rather than Golgi or ER material in the residual body, and the data in Figure 2DF suggests that smnk-1 sperm have normal numbers of MOs and therefore are unlikely to losing MOs in the residual bodies.

Line 273-274 (Fig S4 B). Given how little SMNK-1 is in the spermatids, how do the authors account for the seeming bright labeling of SMNK-1::GFP in the cell body of the spermatozoon? Authors should provide information about how many worms or sperm were imaged to arrive at this assessment. If they are not confident in this result, they should leave it out of the paper.

Line 287 (Fig S4 C). For non-specialists, it will be helpful to indicate the developmental progression along the gonad – to show the earlier expression of the 1CB4 antigen. Based on the images shown (it would be nice to include an enlarged SMNK-1 only image), there may be some co-localization of SMNK-1 and the 1CB4 MO marker, but it is only partial. The statement in the text that SMNK-1 and 1CB4 co-localize is too strong.

Line 298 (Fig 5AB) The authors state that SMNK-1 and SPE-18 partially co-localize in the late pachytene stage, however their image and quantification suggest that the two are typically adjacent rather than co-localizing at that stage. There is partial co-localization in karyosome spermatocytes (5AB) and later stages (S5A) – but the pattern is clearly complex.

The finding that the SPE-18 fails to localize in smnk-1 mutants while conversely SMNK-1 localizes normally in spe-18 mutants is convincing. For line 309 and the data shown, it would be more appropriate to “hypothesize” that SMNK-1 directly or indirectly recruits SPE-18 to the MO surface – since the co-localization results are complicated.

The BFA experiments are nice!

In Figure 6C, what stage spermatocytes are being shown? Based on 6D, there isn’t much lysotracker labelling of meiotic prophase spermatocytes on the rachis – so are these meiotically dividing spermatocytes?

Lines 337-340. Based on several other Spe mutants, the “large abnormal vacuoles” are undoubtedly swollen MOs. It would be worth noting that similar structures have been previously reported in other Spe mutants.

For Figure 6D, it is difficult to compare & contrast the wildtype and vha-18 images shown. In the wildtype image, the karyosome spermatocytes are in the middle of the image. In the vha-18 image, the karyosome spermatocytes are in the right third and then there is a zone in the middle that is not labelling strongly with either SMNK-1:GFP or Lysotracker. Was this pattern something that the authors observed in multiple gonads? Also, the gonads in 6D should be flipped 180oC so that the developmental progression is from left to right (like all other images), and they should label key stages. The authors should either substitute a different mutant gonad OR comment on the additional defects (if the image shown is truly representative).

Line 346-348 In the differentially positioned gonads (Fig 6D), the SMNK-1::GFP patterns seem to be unaltered in the karyosome stage spermatocytes. It may be that SMNK-1 disappears faster in vha-8 mutant gonads. However, the images do not suggest that VHA-8 is required for SMNK-1 localization in developing FB-MO complexes. They do suggest a role in maintaining MO integrity in later meiotic stage spermatocytes.

Figure 7. Without additional context, it is hard to stage the mutant spermatocytes shown. It would be nice to include immunolabeling of wildtype and vha-8 mutant gonad to determine whether vha-8 spermatocytes in meiotic prophase have MO defects (1CB4 labeling) or FB defects (MSP labeling – as done for smnk-1 in Figure 3D) – or whether the vha-8 defects are occurring only after rachis detachment. Authors should emphasize the key differences between the smnk-1 and vha-8 phenotypes by including analogous images.

Line 371. Using the word “multi-nuclei” may be misleading. The chromatin in C. elegans spermatids is not surrounded by a nuclear envelope, so wildtype spermatids have a “chromatin mass” rather than a true nucleus. In the absence of quantifying the amount of DNA, it is unclear if the mutant sperm have > haploid DNA content (consistent with them being multi-nucleate) OR whether what is being shown is a haploid but fragmented chromatin mass.

Line 439- The idea that a v-ATPase in the MO membrane causes a localized alkaline environment on the cytosolic surface which in turn contributes to MSP polymerization is intriguing. However, the data shown (Fig 6 and S6C) does not provide

evidence that VHA-8 is functioning during the initial stages of FB assembly. Instead, it seems to be functioning only after spermatocyte detach from the rachis – during the meiotic divisions and in the budding figures. Furthermore, if the authors hypothesize that an alkaline microenvironment is promoting localized MSP assembly/stability, they need to explain why continued pH gradients in spermatids (which label strongly with lysotracker) are permissive for the disassembly of MSP polymers into dimers.

Minor edits:
Line 50-52. Add Smith (2006) citation here (already in references).
Line 57-61. Move Hu citation to this sentence. Add two references
1) Winter ES, Schwarz A, Fabig G, Feldman JL, Pires-daSilva A, Müller-Reichert T, Sadler PL, Shakes DC. (2017). Cytoskeletal variations in an asymmetric cell division support diversity in nematode sperm size and sex ratios. Development 144(18):3253-3263. doi: 10.1242/dev.153841.
2) Roberts TM, Pavalko FM, Ward S (1986) Membrane and cytoplasmic proteins are transported in the same complex during spermatogenesis. J Cell Biol 102 (5): 1787–1796.
Line 64. Change "encased by the membrane of the MOs" to clarify that the FBs are assembling on the cytosolic face of the MO.
Line 110-111. Intended meaning? As appropriate, edit to "conserved homologs of SMNK-1 in other Caenorhabditis species C. brenneri and C. remanei
Line 124 – reference Figure 1D for statement about brood size.
Line 124-125 Edit the sentence to "However, smnk-1(ibp107) hermaphrodites produce normal numbers of progeny when mated with him-5 males, indicating that smnk-1 infertility defect is due to defective sperm and not defective oocytes." (edit the "rescue" language in the figure legend as well).
Line 127 – replace "Besides" with "In addition"
Line 173 – edit "puncta on the side of cell body" to "a crescent of puncta at the plasma membrane of the cell body". It's hard to see individual puncta in the picture and thus the current wording might be confusing to non-specialist readers.
Line 174. – replace "H2B" with Histone H2B (H2B), at least for the first reference.
Line 179 – refer to Fig. 2A after "spermatheca" in this sentence.
Line 209 – replace "evidences" with "results"
Line 254-262 – In this section which then supports Figure 4, the authors should cite Shakes et al 2009 (DC, Wu JC, Sadler PL, Laprade K, Moore LL, Noritake A, Chu DS. Spermatogenesis-specific features of the meiotic program in Caenorhabditis elegans. PLoS Genet. 2009 Aug;5(8): e1000611) as this was the first publication to clarify some of these stages, especially the karyosome stage.
Line 290. Change. "reminds" to "is reminiscent"
Line 291 – add reference 15 to the list cited here.
Figure 7E – the label vha-18Xhim-5 is hard to read and easy to miss. It would be better to have "self-fertilizing" below the "blue" data and 'him-5 mated" below the "green" data.
Figure 7F – add "outcross" and "self-cross" below the non-dpy and dumpy labels – for clarity
Line 424 - change to "Once spermatids bud off the RB, the FBs begin to disassemble into soluble MSP dimers."
Figure legend 1A – For the RT-PCR result – were these L4s or mixed stage worms?
Figure 1F – for the mitotracker experiments, what are the n values?
Figure 3B&E – what are the n values (sperm? worms?). For "E" what was measured as the spermatocytes vary in intensity by stage?
Figure 4 B – The third red box appears to be a diakinesis stage spermatocyte, but the adjacent text "spermatids" might mislead a non-specialist reader to think that the selected cell was a spermatid.
Figure legend S1C – clarify unmated hermaphrodites; S1D clarify "self-fertility"
Figure legend S7A – why use the language "resembling the lysosome" when these are swollen MOs?
S7B – mCherry labelled chromatin mass (these are not surrounded by nuclear envelopes).
Videos – clarify the time lapse factor.

Reviewer #2

(Remarks to the Author)
This study by Ma et al. identified a novel casein-like kinase named SMNK-1 in the nematode C. elegans, which is necessary for the normal spermiogenesis by promoting major sperm protein (MSP) polymerization and subsequent transportation of MSPs into spermatids. Through a genetic screening, the authors identified the v-ATPase subunit VHA-18, is crucial for spermiogenesis, and loss of vha-18 or inhibition of acidification of membranous organelle resulted in the mis-localization of SMNK-1. The authors also identified that the kinase activity of SMNK-1 is indispensable for recruiting SPE-18 to facilitate MSP condensation and fibrous body (FB) assembly. As a result, loss of SMNK-1 induces the failure of MSP sorting into spermatids, which eventually renders the infertility of worms. Considering the relative intact and innovative storyline of this study, I recommend that this work could be published in Nature Communications. However, there are some issues to be addressed before publication, as listed below.

Major points:
1. Although C. elegans is a useful model organism in cell biological studies, it is quite different from mammals (or humans). Therefore, to better benefit the scientific society, I recommend the authors to discuss how their findings are associated with higher organisms (e.g., the role of SMNK-1 / CK1e in human spermiogenesis or infertility).

2. The statistics section is missing in this manuscript. What statistical methods (t test?) are employed to compare the difference between two groups?

3. The quality of figures should be systematically improved. For instance, in Figure 1B, the coloring was wrong in the last group. In Figure 3C (or 3D, or 6C), the positions of lines should be refined. In figure 7C or 7D, the positions of words were quite disordered.

Minor points:
1. It would be better to show the mean ± SD rather than SEM, as SD reflects the deviation of samples in a group (e.g., in Figure 1B, 1D, 1E…)

2. Line830: The format of symbols should be identical in figures and figure legends. For example, '****p<0.0001'; 'n.s.' ...

3. The temporal scale of the two supplementary movies is lost.

4. Typing error: (line 112) 'Ser / Thr'
Figure 1B: "RNA expression" ...

Reviewer #3

(Remarks to the Author)
I co-reviewed this manuscript with one of the reviewers who provided the listed reports. This is part of the Nature Communications initiative to facilitate training in peer review and to provide appropriate recognition for Early Career Researchers who co-review manuscripts.

Reviewer #4

(Remarks to the Author)
In this paper the authors identify SMNK-1 and show that it is necessary for fibrous body assembly during C. elegans spermatogenesis. They provide evidence that the kinase activity of SMNK-1 is required for this function. Additionally, they investigate whether there is a relationship between SMNK-1 and SPE-18 and test whether VHA-18 has a role in C. elegans spermatogenesis. This is a solid paper and the methodology meets, and in some cases exceeds, the standards in the field. Characterization of two new factors required for spermatogenesis, SMNK-1 and VHA-18, is of interest and importance to the field. Minor points below are suggested for polishing the manuscript and improving readability and ability to interpret the data. The only concern is that the conclusions somewhat overreach what can reasonably be determined by the data presented as noted in the comments below:

• Figure 2A is insufficient to determine if the NKB-2 signal is reduced or to determine whether the hermaphrodite-derived sperm from the smnk-1 mutants are activating in vivo or if there are any defects in MO fusion. While later data support the defects in activation and pseudopod formation of sperm from males, I see no convincing differences between control and mutant in Figure 2A. The legend refers to the "number of GFP puncta" but there was no quantification of the data. I also did not see a methods section related to this (and Figure S2); what age range is a young adult? To better determine the effect of the mutation on hermaphrodite sperm the authors should consider dissecting the hermaphrodite sperm out of the spermatheca.
• I fully agree that there is reduced MSP seen in the mutant in Figure 3A. However, I'm not sure the authors convincingly show that this is entirely due to failure to assemble into FBs and not due to reduced expression or stability of MSP. The RNAseq data in S3 shows a log2fc below 1 for all msp genes, and while it's stated the change is not significant at least msp-3 appears below 0.5 which I believe would be considered significant. qRT-PCR would be better to look at all these genes, and the authors should also take into account that collectively small reduction in every gene could lead to an overall reduction in protein. In 3C the authors state MSP is "completely dispersed in the cytoplasm" (line 233), however their description in the figure legend that it is "diffusive in the cortical area" is more accurate. They also say that part of the MSP is retained in the residual body, but based on the images shown it does not appear that there is more MSP in the residual body of the mutant than there is in the control. Ultimately, it's not clear if it is a partitioning defect which is what is concluded in lines 250 – 251.
• The data in Figure 5 is not sufficient to conclude that SMNK-1 recruits SPE-18 to the surface of MOs. It clearly has an effect on SPE-18 localization/puncta formation, but this effect can be indirect and may regulate some other aspect of SPE-18 association with MOs/FBs other than recruitment. It's worth noting that here the intensity of the signal remains bright even though puncta don't form, which is not what was observed for MSP in the smnk-1 mutant. The authors should tone down the conclusion that SMNK-1 "recruits" SPE-18 throughout the manuscript (including lines 459 – 461). They also show no evidence that SMNK-1 phosphorylates SPE-18, and while this is a viable hypothesis they should be careful how they discuss this in the Discussion section (line 432 is not supported by any data presented).
• In their discussion of VHA-18 the authors should cite the spe-5 papers (SPE-5 is a v-ATPase B subunit) and discuss their results in the context of similarities and differences between the vha-18 and spe-5 phenotypes. Note, treatment with bafilomycin was also done in the 2012 spe-5 paper.
• The authors conclude that the reduced MSP in the smnk-1 mutant spermatids is the reason they fail to activate (line 398), however some MO fusion is seen indicating that partial activation occurs and while the reduced MSP would affect pseudopod size/formation they do not provide evidence how MSP may affect MO fusion or prevent activation (vs the activated sperm can't form a pseudopod).
• The authors conclude that MSP assembly into FBs depends on membrane from the MOs (line 449 – 451), but it's not clear which data they are drawing this conclusion from.
• Based on the DAPI staining in Figure 3D, there are noticeable differences in the germline/nuclear staining/presence of

spermatocytes in the mutants compared to control, especially smnk-1(ibp116). The authors should address this and a similar image of the germline should be shown for the (ibp107) allele. It would be helpful if DIC images of the germlines were shown as well.

Other minor comments:

• Line 35 in Abstract: What is meant by “vectorial transport”? Did the authors mean “vesicular transport”?
• In the introduction section, the authors mention human infertility. Are there any known roles for casein kinases in human sperm? If so, the authors should cite these.
• Lines 40 and 53: “sperm infertility” doesn’t make sense since it is the organism and not the sperm that is “fertile” or “infertile”. Perhaps “infertility due to defects in sperm”?
• Typo in Figure 1D, second and fifth sets of data points labeled “smnk-(ibp107);him-5” should be “smnk-1(ibp107);him-5”
• Line 124: “progenies” should be “progeny”
• Difference in scale bars between Figure 1D and 1E make it difficult to tell if males are more severely sterile/sub-fertile than the hermaphrodites. The authors state in the text the average number of hermaphrodite self-progeny for 1D, it would be helpful if they could similarly state the average number of cross-progeny produced by the mutant males.
• How were the zones determined for Figure 1F? This is not mentioned in the results or methods sections. The cartoon and graph are not mentioned in the figure legend.
• Supplementary Figure 1A: the triangle does not actually point to the T.
• Please indicate which allele(s) of smnk-1 are used in Figure 2.
• Missing the figure legend for Supplementary figure 2B.
• Line 193: when the authors say “remained still” do they mean the sperm were not moving?
• Figure 2D: the smnk-1 sperm looks smaller and the MOs more compact. Is this just the representative image? Because it also seems like the sperm might be smaller in Figure 2A.
• It seems like there’s some polarity to the fused MOs in Figure 2F that isn’t really seen in Figure 2C. I wonder if it would be more accurate to use FM1-43 to quantify the number of fused MOs rather than relying on GFP::NKB-2 which could itself be affected by smnk-1? Alternatively, could the authors co-stain with lysotracker to also identify the unfused MOs in the Pronase exposed sperm? The assumption is that all fuse in the control, while some fail to fuse in the mutant.
• I am not particularly convinced by Figure 3G. The authors do not show with any other MO marker that some MOs or MO components are abnormally segregated to the residual body in the smnk-1 mutant. And they show with their own quantification that the number of MOs in the spermatids is not reduced compared to control (Fig 2E).
• Lines 255 and 256: Don’t need “the” before mitosis or meiosis.
• I assume Figure 4A is from a wild-type animal? This should be clearly stated in the figure legend.
• In Figure 4 it would be helpful for readers if in panels B and C it was clearly labeled that the green signal is SMNK-1 (without having to refer to figure legend).
• Figure S4: The signal is surprisingly bright in the spermatozoa compared to the spermatids. Autofluorescence in the green channel is known to be high in the cell body of the spermatozoa. Showing a negative (no GFP) control would be helpful to know how much signal is from autofluorescence vs GFP. DIC to know where the pseudopod is would also be helpful.
• Figure 4C: It is a bit of a stretch to say that the GFP signal “surrounded” the MSP signal. They do seem to be associated, but the red is not completely enclosed within the green. A more magnified inset of this image would be helpful. In Figure S4C there is only minimal SMNK-1 colocalization with 1CB4. The green and red signals are mostly distinct with limited yellow appearing in the merged image (magnified 2).
• Line 298: They appear to partially co-localize in the karyosome stage, not late pachytene (Fig 5 A and B, colocalization is seen in box c but not box b).
• Can the authors differentiate between defective vesicular acidification vs morphological defects and improperly formed MOs (which they show by EM) using lysotracker staining (lines 341 – 344)? Similarly Figure 6D seems to show a loss of SMNK-1::GFP rather than altered localization.
• I’m not sure what is being show in Figure 7A(b). This is control, but no MO membrane is indicated and the FBs look very different from (a).
• Line 376-377: Figure 7C shows that the MSP looks different, and more MSP ends up in the residual body, but does not convincingly show there is less MSP in the spermatids.
• Maybe move the 1CB4 staining from S4 to the main figure? This data is important for conclusions drawn. Similarly, S6D and S6E feel like they should be in the main figure.
• Figure 7D: Is there a reason the author chose to activate with TEA instead of pronase or zine which tend to be more robust in vitro activators?
• Cite MSP constitutes 80% of proteins in the pseudopod (line 426 – 427).

Reviewer #5

(Remarks to the Author)
I co-reviewed this manuscript with one of the reviewers who provided the listed reports. This is part of the Nature Communications initiative to facilitate training in peer review and to provide appropriate recognition for Early Career Researchers who co-review manuscripts.

Version 1:

Reviewer comments:

Reviewer #2

(Remarks to the Author)
The revision has improved the manuscript and I recommend its publication in Nature Communications.

Reviewer #3

(Remarks to the Author)
I co-reviewed this manuscript with one of the reviewers who provided the listed reports. This is part of the Nature Communications initiative to facilitate training in peer review and to provide appropriate recognition for Early Career Researchers who co-review manuscripts.

Reviewer #4

(Remarks to the Author)
The authors have addressed a number of our concerns and made significant revisions. There are still a few outstanding issues that we feel ought to be addressed before publication.
Major:
Line 109 – 110: Regarding Reviewer 1 comment #2. I can find no evidence that the smnk-1 gene name is an approved name for C09B9.4. smnk-1 does not show up on WormBase or the Genome Alliance database or Uniprot. All instances of smnk-1 in the manuscript should be replaced with spe-55, which is the community approved and searchable name for this gene.

Line 490 – 492: Revise to "SMNK-1 functions upstream of SPE-18 and its kinase activity is essential for the localization of SPE-18 on the MO 492 surface in spermatocytes."
To state that SMNK-1 functions "as an upstream protein kinase of SPE-18" implies direct regulation and phosphorylation that were not shown in this study.

Minor:
Line 62 – 68: I understand the authors were trying to improve the accuracy of their statement, but this sentence is confusing as written. We suggest at least simplifying to: "During the meiosis II sorting stage of spermatogenesis in C. elegans, the developing spermatid containing DNA, some mitochondria, and membranous organelles (MOs) is budded away from the residual body (RB), where unnecessary components, such as the endoplasmic reticulum, ribosomes, tubulin, and actin, are disposed.

Fix gene labels in Sup Fig S1A.

Title and legend for Sup Fig S2 should be revised. The whole manuscript presents data that FB assembly during spermatogenesis requires SPE-55, so saying it does not affect spermatogenesis is inaccurate. I believe the authors mean to say that it does not affect the number of spermatids produced.

Line 154: Remove "rescue" language here and elsewhere. In cases of sperm-specific defects hermaphrodites can produce normal numbers of progeny when mated to wildtype males, but this is not a "rescue" experiment. The genetic defect of the hermaphrodite is not rescued.

Lines 192 to 198 need to be rephrased. Simply reading them is confusing. The authors first state that there was no difference in hermaphrodite spermatids before ovulation and after ovulation the spermatids activated. This implies that spermatogenesis and sperm activation are unaffected in the hermaphrodite. But then they end the paragraph that the data "provided a cue for us to test the impact of the smnk-1 mutation on spermatogenesis". While we think it is important that the authors acknowledged our initial concern that the data are insufficient to determine if the NKB-2 signal is reduced, it does not logically transition to the conclusion that they should look at spermatogenesis in more detail or that they should look at male-derived sperm.

Lines 200-203: The sentence repeats itself, and without quantification the authors can't definitively say they have a "normal number". Shorten to: "There was no obvious difference in the number of spermatids produced between the mutant and WT animals (Supplementary Fig. 2B)."

From our comment in the original review: Please indicate which allele(s) of smnk-1 are used in Figure 2. The allele name is now mentioned in the beginning of the results sub-section, but not in the figure or the figure legend itself. We recommend the explanation given in the rebuttal, "for the smnk-1 allele shown in Figure 2 and other unlabeled instances, it is ibp107, which is a frameshift mutation", should be mentioned in the manuscript to avoid confusion.

Figure 6 – stage of spermatocytes being shown should be specified in figure legend (per comment from Reviewer 1)

Line 372: Authors cite Reference 23 when it should be Reference 22

Line 377: Per Reviewer 1 "Based on several other Spe mutants, the "large abnormal vacuoles" are undoubtedly swollen MOs. It would be worth noting that similar structures have been previously reported in other Spe mutants." While the authors

changed the language to refer to the observed structures as swollen MOs, they failed to note that similar structures have been reported previously or provide any citations to these previous observations that inform their conclusion.

Line 392: There is no Supplementary Figure 6H

Line 395 – 397: It is not clear what is meant by “exhibited less of a reduction compared to that of the control group”. The language provided in the Response to Reviewers is much clearer. We suggest using this same language in the manuscript: “we also observed variation in the SMNK-1::GFP signal within the germline of vha-18;him-5 mutants, with some individuals displaying a modest reduction, whereas others revealed a significant decrease compared to the control group.”

Reviewer comments not addressed, even though rebuttal says they were:

Line 124 – reference Figure 1D for statement about brood size. Now lines 135 -138.

Line 50-52. Add Smith (2006) citation here (already in references). This reference is now completely omitted. Should be cited on line 58 of revised manuscript.

Line 57-61. Move Hu citation to this sentence. Add two references
1) Winter ES, Schwarz A, Fabig G, Feldman JL, Pires-daSilva A, Müller-Reichert T, Sadler PL, Shakes DC. (2017). Cytoskeletal variations in an asymmetric cell division support diversity in nematode sperm size and sex ratios. Development 144(18):3253-3263. doi: 10.1242/dev.153841.
2) Roberts TM, Pavalko FM, Ward S (1986) Membrane and cytoplasmic proteins are transported in the same complex during spermatogenesis. J Cell Biol 102 (5): 1787–1796.
None of these citations are in the correct place. Should be cited at the end of line 68 of revised manuscript.

Difference in scale bars between Figure 1D and 1E make it difficult to tell if males are more severely sterile/sub-fertile than the hermaphrodites. The authors state in the text the average number of hermaphrodite self-progeny for 1D, it would be helpful if they could similarly state the average number of cross-progeny produced by the mutant males.

Reviewer #5

(Remarks to the Author)
I co-reviewed this manuscript with one of the reviewers who provided the listed reports. This is part of the Nature Communications initiative to facilitate training in peer review and to provide appropriate recognition for Early Career Researchers who co-review manuscripts.

Version 2:

Reviewer comments:

Reviewer #4

(Remarks to the Author)
1. The authors have replaced smnk-1 with spe-55 throughout the manuscript and corresponding figures. However, they did not replace smnk-1 in the REAGENT or RESOURCE SOURCE IDENTIFIER. A simple search shows 26 instances of "smnk-1" still in the manuscript in the strain list, oligonucleotides, and recombinant DNA.

2. Lines 508-510 are "MOs’ lumen, thereby making the immediate cytoplasmic microenvironment around MOs more alkaline. This result is consistent with a previous study in which a higher pH is favorable for MSP polymerization at the leading edge of". This is not the text the authors claim to have revised which was meant to be "SMNK-1 functions upstream of SPE-18 and its kinase activity is essential for the localization of SPE-18 on the MO surface in spermatocytes."

3. Where it was suggested the authors indicate which allele(s) are used in Figure 2 the added sentence "For the spe-55 allele shown in other unlabeled is ibp107 allele." could use to be revised for clarity. [Please also note this is lines 132-133, not 134-135 as stated in the rebuttal.]

4. Previous comment: Line 50-52. Add Smith (2006) citation here (already in references). This reference was then omitted in the revision. Should have been cited on line 58 of revised manuscript.
Author reply: We thank the reviewer for pointing out this oversight. We agree that the work of
Smith (2011) is highly relevant here. We have incorporated this reference into the revised manuscript. (Line 59)
Note: Smith (2011) is not the right reference. Smith (2006) that was referenced in the original manuscript is the reference that should be added back.

Reviewer #5

(Remarks to the Author)
I co-reviewed this manuscript with one of the reviewers who provided the listed reports. This is part of the Nature Communications initiative to facilitate training in peer review and to provide appropriate recognition for Early Career Researchers who co-review manuscripts.

Open Access This Peer Review File is licensed under a Creative Commons Attribution 4.0 International License, which permits use, sharing, adaptation, distribution and reproduction in any medium or format, as long as you give appropriate credit to the original author(s) and the source, provide a link to the Creative Commons license, and indicate if changes were made.
In cases where reviewers are anonymous, credit should be given to 'Anonymous Referee' and the source.
The images or other third party material in this Peer Review File are included in the article’s Creative Commons license, unless indicated otherwise in a credit line to the material. If material is not included in the article’s Creative Commons license and your intended use is not permitted by statutory regulation or exceeds the permitted use, you will need to obtain permission directly from the copyright holder.
To view a copy of this license, visit https://creativecommons.org/licenses/by/4.0/

Ma et al. **Nature Communication** Tracking #: NCOMMS-25-11037A

Response to reviewer comments

We sincerely thank the five reviewers for their positive and constructive feedback on our manuscript. We also appreciate their recognition that our study provides valuable insights into the co-regulation of FB-MO complex assembly and transport by SMNK-1 and VHA-18 during *C. elegans* spermatogenesis.

In response to all of the reviewers' comments and suggestions, we have thoroughly revised the manuscript. Below, we provide detailed, point-by-point responses to each of the reviewer's remarks. Reviewer comments are quoted in blue italics, followed by our replies in black Roman font. In addition, we have engaged a native English speaker to polish the language throughout the manuscript.

We believe that these revisions, including the updated title, have substantially strengthened the manuscript. We hope that the revised version will now meet the standards required for publication in ***Nature Communications***.

Reviewer #1 (Remarks to the Author):

In nematode spermatocytes, localized assembly of the major sperm protein (MSP) into fibrous bodies is critical for efficient post-meiotic partitioning of this key motility protein to the haploid sperm. We are only just beginning to understand how these fibrous bodies assemble on the cytosolic side of Golgi-derived membranous organelles. Previous work identified involvement of an intrinsically disordered protein SPE-18, this study by Ma et al greatly expands our understanding of the assembly process by identifying specific role for the CK1 related kinase (SMNK-1) in the fibrous body assembly process – which occurs in meiotic prophase spermatocytes. In addition, they show a role for the v-ATPase in MO integrity in meiotic stage and budding figure spermatocytes. The greater significance of this work is that it highlights an experimentally accessible example of how cytoskeletal elements can assemble adjacent to a biological membrane with distinct contributions of a regulatory kinase and intrinsically disordered protein as well as role for a localized alkaline microenvironment during later stages of the process. This work should be of interest to a variety of readers from those interested in gametogenesis to those interested in liquid condensate mediated complex assembly. There is a lot of nice work in this paper but there are issues in the text and documentation of the experiments that need to be addressed.

We sincerely thank Reviewer 1 for their overall enthusiasm and valuable suggestions regarding our manuscript.

1. The title, the abstract (line 28 "meanwhile") and the end of the introduction (Line 89-92) are a bit misleading as they could easily give the impression that the alkaline

environment is critical for the initial assembly of fibrous bodies in the meiotic prophase spermatocytes. The actual data shows a role for VHA-8 in later meiotic stage spermatocytes (MO integrity) and post-anaphase II budding figures (efficient MSP segregation). Thus VHA-8 may be more important in maintaining the integrity of the MOs and either directly or indirectly the already formed or final growth stages of the FBs. The authors should clarify these distinctions in these instances and throughout the paper. SMNK-1 is important for the initial assembly of the FB-MO complex assembly (especially the FB) whereas VHA-8 may be more important of maintaining the complex (especially the MO) once the FB-MO complex has already formed.

Thank you for your insightful suggestions, and we fully agree with your interpretation based on the data. SPE-55/SMNK-1 is primarily involved in the initial assembly of the FB-MO complex, especially the FB, whereas VHA-8 plays a more critical role in maintaining the FB-MO complex, especially the MO. Accordingly, we have revised the description of VHA-18 throughout the manuscript to reflect this. The title has also been updated as follows:

SPE-55/SMNK-1 mediated fibrous body assembly and VHA-18-dependent membranous organelle maintenance regulate efficient major sperm protein transport during *Caenorhabditis elegans* spermatogenesis

We also revised the abstract as follows: "Furthermore, we discovered that the H subunit of v-ATPase, which was localized on MOs, played a crucial role in maintaining MO morphology and function, promoting FB–MO connections, and thus ensuring efficient MSP sorting during spermatogenesis."

2. Naming of the C09B9.4 gene. Gene names in C. elegans must be approved in advanced. It is unclear why C09B9.4 has not been given a Spe (spermatogenesis defective) designation since it clearly fits within this class of genes. Has the name smnk-1 been approved by S. L'Hernault and/or Tim Schedl?
Indeed, *C09B9.4* is a *Spe* gene. We contacted Drs. Steven L'Hernault and Tim Schedl to seek their approval of this new Spe designation, and they named this gene as *spe-55/smnk-1*, where *spe-55* and *smnk-1* refer to the same gene.

3. In supplementary figure S1A, there is a problem with the amino acid numbering as the aligned proteins are not all the same length and the arrow that is supposed to indicate a critical conserved Thr is pointing to a glycine. Fix in the figure and edit main text appropriately.
We appreciate your careful observation. We have modified Figure S1A and edited the main text.

4. Paragraph 149-154 should be moved up – before the section on the spe-8 crosses.
Thank you for your helpful advice. We have moved these descriptions to before the section on *spe-8* crosses.

5. Line. 148 – the conclusion from the spe-8 crosses should be that 1) the males have a fertility severe fertility defect, 2) their seminal fluid contains functional sperm activator, and 3) the smnk-1 male sperm does not out-compete the spe-8 hermaphrodite sperm.

Thank you for this valuable suggestion. We have supplemented the relevant descriptions, and the corresponding revision can be found at lines 167–170 in the revised manuscript.

6. In Figure 2A, it would be helpful to include H2B in the enlarged images of the spermatozoa to give a better sense of how many sperm are being shown in the mutant image.

Thank you for your suggestion. We have revised Figure 2A as recommended.

7. Line 186 – change "apparent" to "immediately apparent" since the analysis of potential germline defects is limited. Authors could note or quantify if a normal number of spermatids are produced.

Thank you for this helpful advice. We have revised the expression from 'apparent' to 'immediately apparent'. We also noted in the revised manuscript, "There was no obvious difference in the number of spermatids produced between the mutant and WT animals, and smnk-1 mutants exhibited a normal number of spermatids compared to that of the WT animals (Supplementary Fig. 2B)." (see lines 200–203)

8. Videos of sperm within swm-1 males is a somewhat artificial environment. However, this result is paired with other lines of evidence; so, it is ok. A better experiment would be to video the GFP::NKB-2 male sperm (+/- the smnk-1 mutation) within the uterus of feminized non-smnk-1 worms.

We fully understand your concerns. The *swm-1* phenotype is a robust and reproducible result that we confirmed in multiple independent experiments. In addition, we successfully obtained videos of GFP::NKB-2 male sperm (+/- the *smnk-1* mutation) within the uterus of feminized non-*smnk-1* worms. Sperm from *smnk-1* mutant males were dispersed in the uterus and rarely reached the spermatheca of feminized worms, whereas sperm from control males mostly accumulated in the spermatheca. We have added this data to Video S3 and Video S4. These data are consistent with Figure 1F, where sperm from *smnk-1; him-5* exhibit defective motility.

9. The studies documenting the defects in MO fusion but not MO numbers are nice! It is worth noting that fewer MOs fuse in smnk-1 sperm – but that their data suggest that all sperm respond to activation by fusing at least some MOs (even if the sperm aren't extending pseudopods). The authors should make the point that all sperm are responding to activation – albeit weakly and with defects.

Thank you for your suggestion. We have revised our description for sperm activation as suggested. (see lines 242–246)

10. Documenting the failure of MSP to assemble into FBs with both anti-MSP immunofluorescence and TEM is nice. The images for Fig3F are very nice. Interpretation of the budding figure (Fig3G) is less clear. It's hard to tell if that is an MO material rather than Golgi or ER material in the residual body, and the data in Figure 2DF suggests that smnk-1 sperm have normal numbers of MOs and therefore are unlikely to losing MOs in the residual bodies.

Thank you for your recognition of our anti-MSP immunofluorescence and TEM imaging data, and we appreciate your valuable comments. Reviewer 3 raised the same criticism about Figure 3G. After double-checking our data, we agree that these structures are unlikely to be the MOs. We have revised the legend of Figure 3G (see

lines 1039–1042) and the description of budding in Figure 3G in the revised manuscript. (see lines 272–274)

11. Line 273-274 (Fig S4 B). Given how little SMNK-1 is in the spermatids, how do the authors account for the seeming bright labeling of SMNK-1::GFP in the cell body of the spermatozoon? Authors should provide information about how many worms or sperm were imaged to arrive at this assessment. If they are not confident in this result, they should leave it out of the paper.

We have repeated this assay and observed that the SMNK-1::GFP fluorescence signal in the spermatozoon cell body was extremely faint after removing the *him-5* background fluorescence. Therefore, relevant images and descriptions corresponding to this observation have been removed from the manuscript as suggested. Thank you for this wonderful advice.

12. Line 287 (Fig S4 C). For non-specialists, it will be helpful to indicate the developmental progression along the gonad – to show the earlier expression of the 1CB4 antigen. Based on the images shown (it would be nice to include an enlarged SMNK-1 only image), there may be some co-localization of SMNK-1 and the 1CB4 MO marker, but it is only partial. The statement in the text that SMNK-1 and 1CB4 co-localize is too strong.

Thank you for this helpful advice. We have adjusted the figure and annotated the corresponding developmental stage in Figure 4E (originally S4C). Indeed, SMNK-1 and 1CB4, the MO marker, do not completely colocalize with each other. We have thus revised the corresponding description in the manuscript according to this reviewer's advice. (see lines 316–318)

13. Line 298 (Fig 5AB) The authors state that SMNK-1 and SPE-18 partially co-localize in the late pachytene stage, however their image and quantification suggest that the two are typically adjacent rather than co-localizing at that stage. There is partial co-localization in karyosome spermatocytes (5AB) and later stages (S5A) – but the pattern is clearly complex.

We really appreciate your valuable comments. We fully agree that in the pachytene stage, these two proteins are typically adjacent to each other. The partial colocalization or association begins from the karyosome stage. Therefore, we have revised the relevant description in the manuscript to improve clarity and avoid misunderstanding. (see lines 326–331)

14. The finding that the SPE-18 fails to localize in smnk-1 mutants while conversely SMNK-1 localizes normally in spe-18 mutants is convincing. For line 309 and the data shown, it would be more appropriate to "hypothesize" that SMNK-1 directly or indirectly recruits SPE-18 to the MO surface – since the co-localization results are complicated.

Thank you for this helpful advice. We have revised our statement as suggested. (see lines 340–343)

15. The BFA experiments are nice! In Figure 6C, what stage spermatocytes are being shown? Based on 6D, there isn't much lysotracker labelling of meiotic prophase spermatocytes on the rachis – so are these meiotically dividing spermatocytes?

Thank you for your positive comments about the BFA experiments. The spermatocytes that we dissected were mainly meiotically dividing spermatocytes. This

information has been added to the manuscript and annotated in the corresponding figures. (see lines 362–364)

16. Lines 337-340. Based on several other Spe mutants, the "large abnormal vacuoles" are undoubtedly swollen MOs. *It would be worth noting that similar structures have been previously reported in other Spe mutants.*
Thank you for this helpful advice. We agree that the "large abnormal vacuoles" are undoubtedly swollen MOs. Therefore, we have revised our statement as suggested, with swollen MOs throughout the manuscript.

17. For Figure 6D, it is difficult to compare & contrast the wildtype and vha-18 images shown. In the wildtype image, the karyosome spermatocytes are in the middle of the image. In the vha-18 image, the karyosome spermatocytes are in the right third and then there is a zone in the middle that is not labelling strongly with either SMNK-1:GFP or Lysotracker. Was this pattern something that the authors observed in multiple gonads? Also, the gonads in 6D should be flipped 180ºC so that the developmental progression is from left to right (like all other images), and they should label key stages. The authors should either substitute a different mutant gonad OR comment on the additional defects (if the image shown is truly representative).

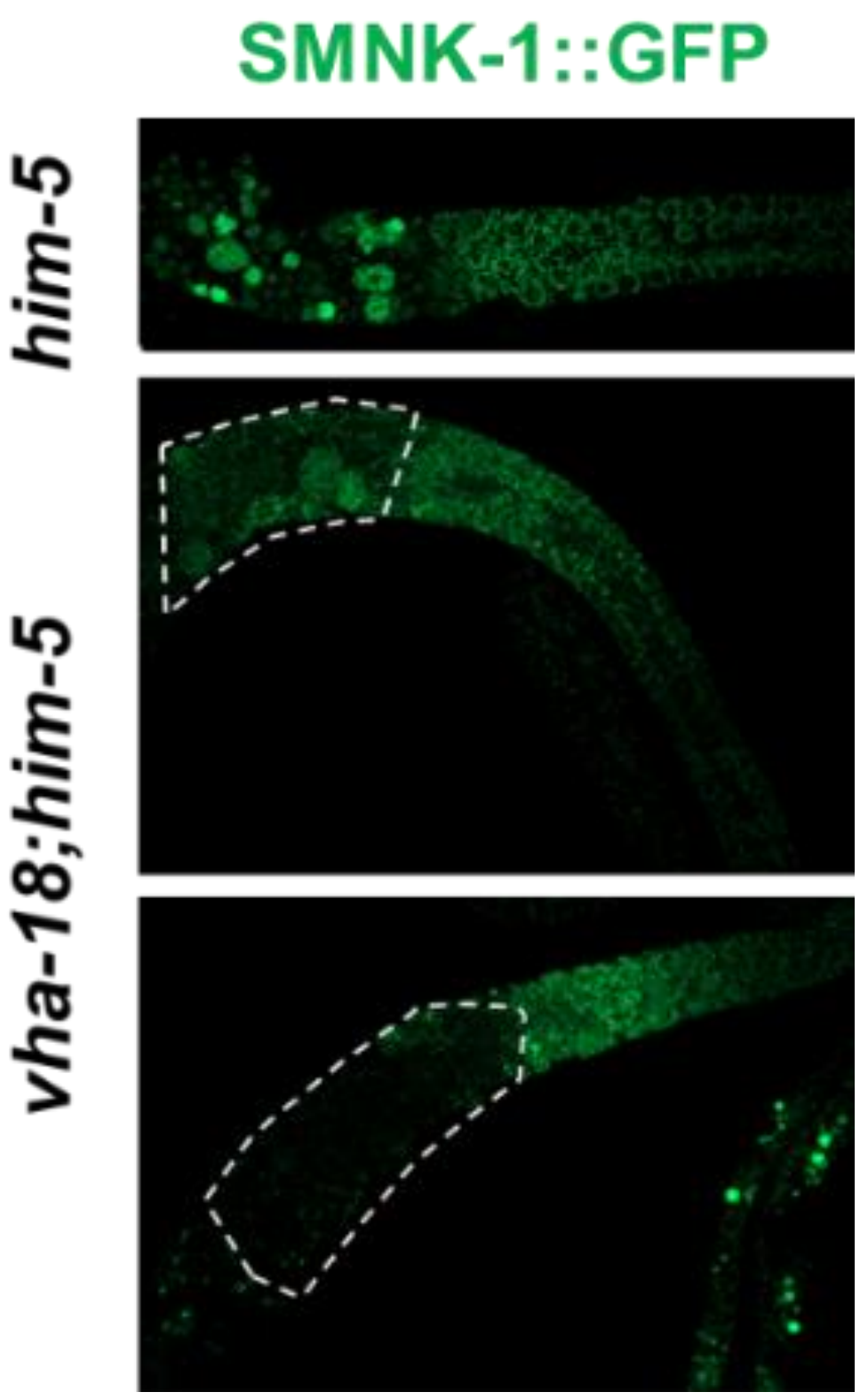

Figure R1

Thank you for your valuable comments. Yes, we observed this pattern in our numerous and repeated experiments. We have adjusted the orientation of the gonads in Figure 6G (originally 6D) and indicated the corresponding developmental stages based on the nucleus morphology. In addition, we adjusted the images to ensure that the positions of the control and *smnk-1;him-5* groups at the same stage are consistent in the images. The reason why there is a zone in the middle with weak or no labeling

of either SMNK-1::GFP or Lysotracker is that sperm development may become abnormal starting from this region under the *vha-18* mutant background. VHA-18 loss disrupts the MO structure and localization of SMNK-1::GFP, which leads to its faster disappearance compared to the control. Meanwhile, we also observed variation in the SMNK-1::GFP signal within the germline of *vha-18;him-5* mutants, with some individuals displaying a modest reduction, whereas others revealed a significant decrease compared to the control group. We suppose that this discrepancy may arise from the differential effects of the vha-18 mutation across individuals. As shown in Figure R1, we have included representative images illustrating the two distinct phenotypic classes observed in *vha-18* mutants and have correspondingly revised the relevant descriptions in the manuscript. Moreover, the Lysotracker staining signal is weaker in meiotically dividing spermatocytes than in spermatids; this is likely due to reduced acidity and a higher pH environment. We need to maintain consistent parameters during imaging; therefore, it looks as if there is no labeling of either SMNK-1::GFP or Lysotracker in the germline.

18. Line 346-348 In the differentially positioned gonads (Fig 6D), the SMNK-1::GFP patterns seem to be unaltered in the karyosome stage spermatocytes. It may be that SMNK-1 disappears faster in vha-8 mutant gonads. However, the images do not suggest that VHA-8 is required for SMNK-1 localization in developing FB-MO complexes. They do suggest a role in maintaining MO integrity in later meiotic stage spermatocytes.

Thank you for your comments. SMNK-1::GFP may disappear more rapidly in *vha-18* mutant gonads. As SMNK-1::GFP localization is closely associated with MO structures, we propose that *vha-18* plays an important role in maintaining the MO structure and function. Thus, the disrupted MO integrity in *vha-18* mutants likely results in the altered SMNK-1::GFP localization. We acknowledge that our original description was misleading and have revised the text accordingly (see lines 392-397).

19. Figure 7. Without additional context, it is hard to stage the mutant spermatocytes shown. It would be nice to include immunolabeling of wildtype and vha-8 mutant gonad to determine whether vha-8 spermatocytes in meiotic prophase have MO defects (1CB4 labeling) or FB defects (MSP labeling – as done for smnk-1 in Figure 3D) – or whether the vha-8 defects are occurring only after rachis detachment. Authors should emphasize the key differences between the smnk-1 and vha-8 phenotypes by including analogous images.

Thank you for this valuable comment. We agree that, in principle, immunofluorescence could provide a better assessment of MO and FB defects in the *vha-18* mutant background. However, due to the specific structure of the gonad, we were unable to obtain satisfactory immunofluorescence signals despite testing multiple fixations and permeabilization methods. Although some images were captured, the poor permeabilization resulted in weak and unclear antibody staining. Representative immunofluorescence images have been included in Figure R2.

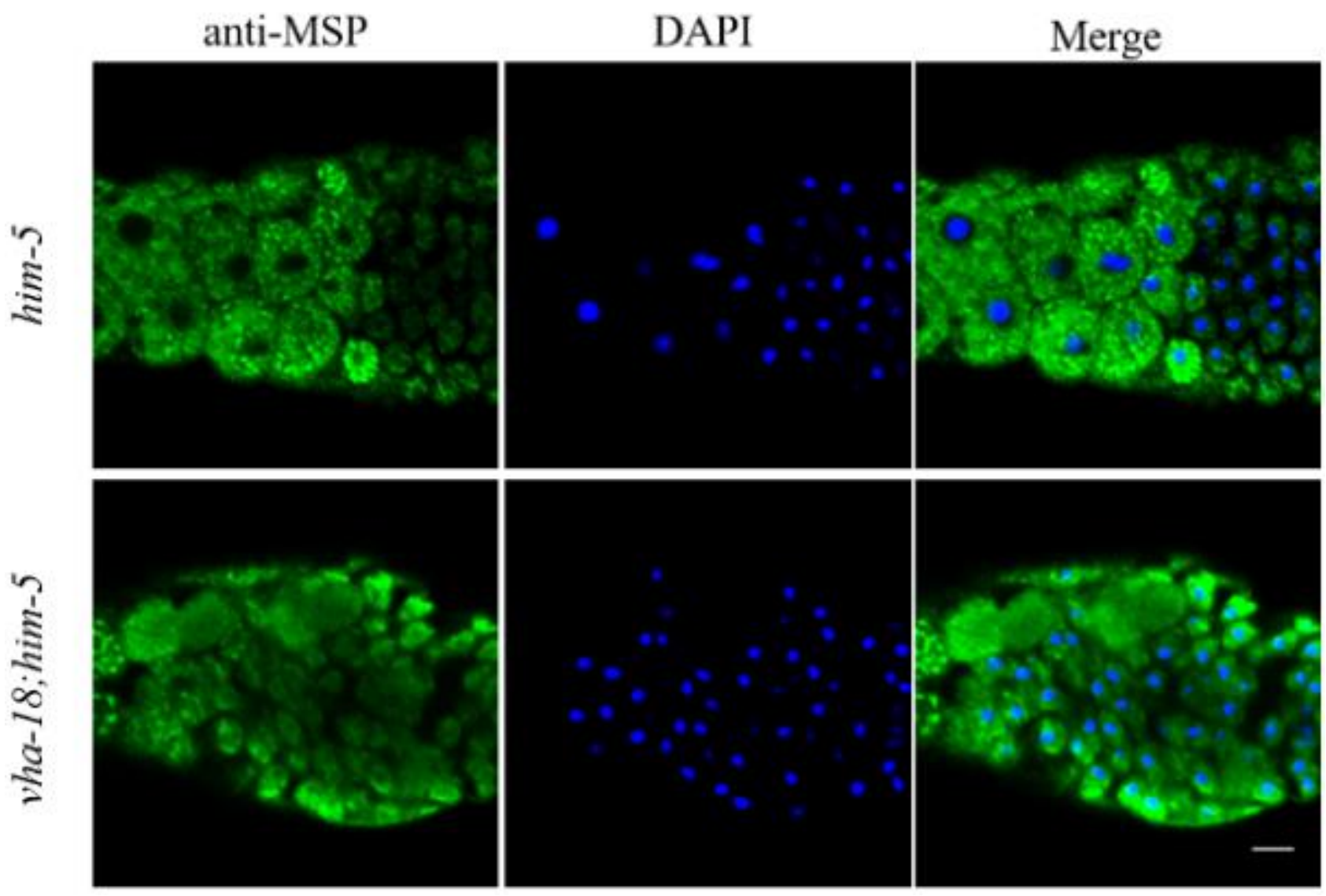

Figure R2

Importantly, the TEM results unambiguously revealed that MO fails to form properly in early and late spermatocytes (Fig. 7A) and spermatids (Fig. 7B) of *vha-18* mutants. Moreover, we added GFP::NKB-2 imaging in *him-5* and *vha-18* backgrounds, which further supports that *vha-18* mutants display early defects during spermatogenesis (Fig. S6D).

20. Line 371. Using the word "multi-nuclei" may be misleading. The chromatin in C. elegans spermatids is not surrounded by a nuclear envelope, so wildtype spermatids have a "chromatin mass" rather than a true nucleus. In the absence of quantifying the amount of DNA, it is unclear if the mutant sperm have > haploid DNA content (consistent with them being multi-nucleate) OR whether what is being shown is a haploid but fragmented chromatin mass.
Thank you for this suggestion. We have revised our statement and used the term "chromatin mass."

21. Line 439- The idea that a v-ATPase in the MO membrane causes a localized alkaline environment on the cytosolic surface which in turn contributes to MSP polymerization is intriguing. However, the data shown (Fig 6 and S6C) does not provide evidence that VHA-8 is functioning during the initial stages of FB assembly. Instead, it seems to be functioning only after spermatocyte detach from the rachis – during the meiotic divisions and in the budding figures. Furthermore, if the authors hypothesize that an alkaline microenvironment is promoting localized MSP assembly/stability, they need to explain why continued pH gradients in spermatids (which label strongly with lysotracker) are permissive for the disassembly of MSP polymers into dimers.
We appreciate your insightful comments. Our VHA-18::mCherry data demonstrate that VHA-18 is already detectable in early spermatocytes, thus indicating that its function might begin before spermatocytes detach from the rachis. Consistently, our TEM results reveal that the MO morphology is abnormal at these early stages in *vha-18* mutants, which supports that VHA-18 plays a crucial role in maintaining MO function from the onset of FB–MO formation. There is no direct mechanistic

relationship between VHA-18 and MSP, but rather an indirect effect that is mediated through the integrity of MO membranes.
We propose a hypothesis that VHA-18 regulates the MO lumen pH and thus its morphology and function, which might indirectly facilitate the association of MO with FB, which is regulated by SMNK-1. Taken together, VHA-18 and SMNK-1 act in parallel but complementary pathways to coordinate the proper assembly and function of the FB–MO complex. (see lines 346–356 and lines 498–502)

Minor edits:
Line 50-52. Add Smith (2006) citation here (already in references).
Thank you for this advice; we have cited the reference as suggested.

Line 57-61. Move Hu citation to this sentence. Add two references
1) Winter ES, Schwarz A, Fabig G, Feldman JL, Pires-daSilva A, Müller-Reichert T, Sadler PL, Shakes DC. (2017). Cytoskeletal variations in an asymmetric cell division support diversity in nematode sperm size and sex ratios. Development 144(18):3253-3263. doi: 10.1242/dev.153841.
2) Roberts TM, Pavalko FM, Ward S (1986) Membrane and cytoplasmic proteins are transported in the same complex during spermatogenesis. J Cell Biol 102 (5): 1787–1796.
Thank you for your advice; we have cited the suggested references.

Line 64. Change "encased by the membrane of the MOs" to clarify that the FBs are assembling on the cytosolic face of the MO.
Thank you for the advice. We have modified our expression as follows:
"Subsequently, the MSPs are polymerized into filaments, which are hexagonally packed and assembled on the cytosolic face of the MO, forming the MO-associated fibrous body (FB) complex, named the FB–MO complex." (see lines 70–73)

Line 110-111. Intended meaning? As appropriate, edit to "conserved homologs of SMNK-1 in other Caenorhabditis species C. brenneri and C. remanei.
Thank you for your advice. We have modified our expression based on your suggestion as follows: "BLASTP analysis identified two highly conserved SMNK-1 homologs in other *Caenorhabditis* species, *C. brenneri* and *C. remanei* (Supplementary Fig. 1A)." (see lines 122–124)

Line 124 – reference Figure 1D for statement about brood size.
Thank you for your advice. We have modified our statement according to your advice as follows,
"Each control hermaphrodite could produce brood sizes of more than 200, while each *smnk-1(ibp107*) hermaphrodite only produced brood sizes of about 10 because of defective sperm or oocytes, thus making them nearly sterile." (see lines 135–138)

Line 124-125 Edit the sentence to "However, smnk-1(ibp107) hermaphrodites produce normal numbers of progeny when mated with him-5 males, indicating that smnk-1 infertility defect is due to defective sperm and not defective oocytes." (edit the "rescue" language in the figure legend as well).
Thank you for your advice. We have modified our statement based on your suggestion as follows: "However, *smnk-1(ibp107)* hermaphrodites produce normal numbers of

progeny when mated with *him-5* males, which indicates that the *smnk-1* infertility defect is due to defective sperm and not defective oocytes." (see lines 138–140)
Also, we have modified the corresponding figures S1C and S1D legend as follows.
"*Figure S1C*: (C)The number of oocytes laid by unmated hermaphrodites of the *smnk-1(ibp107)*;*him-5* and *him-5* control strains.
Figure S1D: The self-fertility of *smnk-1 (ibp107)* hermaphrodites was not temperature-sensitive. There was no significant difference in hermaphrodite self-fertility of *smnk-1(ibp107)* at 15°C, 20°C, and 25°C. Values in (C) and (D) are presented as the mean ± standard deviation (SD). ****p < 0.0001.
(see lines 1199–1202)

Line 127 – replace "Besides" with "In addition"
Thank you for your advice; we have replaced this expression.

Line 173 – edit "puncta on the side of cell body" to "a crescent of puncta at the plasma membrane of the cell body". It's hard to see individual puncta in the picture and thus the current wording might be confusing to non-specialist readers.
Thank you for your helpful advice; we have replaced this expression.

Line 174. – replace "H2B" with Histone H2B (H2B), at least for the first reference.
Thank you for your helpful reminder; we have replaced "H2B" with Histone H2B (H2B).

Line 179 – refer to Fig. 2A after "spermatheca" in this sentence.
Thank you for your advice; we have revised and referred to Fig. 2A after "spermatheca".

Line 209 – replace "evidences" with "results"
Thank you for your advice; we have replaced this expression.

Line 254-262 – In this section which then supports Figure 4, the authors should cite Shakes et al 2009 (DC, Wu JC, Sadler PL, Laprade K, Moore LL, Noritake A, Chu DS. Spermatogenesis-specific features of the meiotic program in Caenorhabditis elegans. PLoS Genet. 2009 Aug;5(8): e1000611) as this was the first publication to clarify some of these stages, especially the karyosome stage.
Thank you for your valuable suggestion. We have cited these references in the revised manuscript.

Line 290. Change. "reminds" to "is reminiscent."
Thank you for your advice; we have replaced this expression.

Line 291 – add reference 15 to the list cited here.
Thank you very much. We have added the citation.

Figure 7E – the label vha-18Xhim-5 is hard to read and easy to miss. It would be better to have "self-fertilizing" below the "blue" data and 'him-5 mated" below the "green" data.
Thank you for these valuable suggestions. We have clearly indicated the label as suggested in the revised manuscript.

Figure 7F – add "outcross" and "self-cross" below the non-dpy and dumpy labels – for clarity
Thank you for your valuable suggestion. We have now clearly indicated the label as suggested in the revised manuscript.

Line 424 - change to "Once spermatids bud off the RB, the FBs begin to disassemble into soluble MSP dimers."
Thank you for your valuable advice; we have revised our statement.

Figure legend 1A – For the RT-PCR result – were these L4s or mixed stage worms?
Thank you for pointing out the lack of clarity in our original description. For the RT-PCR results, the strains used in this experiment were at the day 1 adult stage. We have added this description to the legend of Figure 1A in the revised manuscript.

Figure 1F – for the mitotracker experiments, what are the n values?
Thank you for your question. The "n values" in this experiment refer to the number of measured germlines based on the MitoTracker fluorescence intensities within the zones indicated in the diagram. We have included this clarification in the figure legend of the revised manuscript.

Figure 3B&E – what are the n values (sperm? worms?). For "E" what was measured as the spermatocytes vary in intensity by stage?
Thank you for bringing this important point to our attention that we had previously overlooked. The n value represents the number of sperm in Figure 3B and spermatocytes in Figure 3E. In 3E, we measured the average fluorescence intensity primarily in spermatocytes with apparent FB–MOs in the wild-type corresponding region in the *smnk-1;him-5* mutant based on the nucleus morphology. We have added the corresponding clarification in the figure legends of the revised manuscript.

Figure 4 B – The third red box appears to be a diakinesis stage spermatocyte, but the adjacent text "spermatids" might mislead a non-specialist reader to think that the selected cell was a spermatid.
We appreciate the reviewer's careful observation. Indeed, the third red box in Figure 4B mistakenly indicated a diakinesis-stage spermatocyte rather than a spermatid. This has been corrected, and the figure has been appropriately relabeled in the revised manuscript.

Figure legend S1C – clarify unmated hermaphrodites; S1D clarify "self-fertility"
Thank you for your advice. We have supplied a description in Figure legend S1C and S1D. For Figure legend S1C, our description is changed to "The number of oocytes laid by unmated hermaphrodites of the *smnk-1(ibp107);him-5* and *him-5* control strains." For Figure legend S1D, our description is changed to "The self-fertility of smnk-1 (ibp107) hermaphrodites was not temperature-sensitive. There was no significant difference in hermaphrodite self-fertility of *smnk-1(ibp107)* at 15°C, 20°C, and 25°C. Values in (C) and (D) are presented as the mean ± standard deviation (SD). ****p < 0.0001."

Figure legend S7A – why use the language "resembling the lysosome" when these are swollen MOs?
Thank you for your valuable comment. We have revised the Figure legend S7A, replacing "resembling the lysosome" with swollen MOs in the revised manuscript.

S7B – mCherry labelled chromatin mass (these are not surrounded by nuclear envelopes).
Thank you for your valuable comment. We have amended our statement to "abnormal chromatin mass" in the revised manuscript.

Videos – clarify the time lapse factor.
Thank you for your valuable comment. We have added the time-lapse factor in the videos as suggested.

Reviewer #2 (Remarks to the Author):

This study by Ma et al. identified a novel casein-like kinase named SMNK-1 in the nematode C. elegans, which is necessary for the normal spermiogenesis by promoting major sperm protein (MSP) polymerization and subsequent transportation of MSPs into spermatids. Through a genetic screening, the authors identified the v-ATPase subunit VHA-18, is crucial for spermiogenesis, and loss of vha-18 or inhibition of acidification of membranous organelle resulted in the mis-localization of SMNK-1. The authors also identified that the kinase activity of SMNK-1 is indispensable for recruiting SPE-18 to facilitate MSP condensation and fibrous body (FB) assembly. As a result, loss of SMNK-1 induces the failure of MSP sorting into spermatids, which eventually renders the infertility of worms. Considering the relative intact and innovative storyline of this study, I recommend that this work could be published in Nature Communications. However, there are some issues to be addressed before publication, as listed below.
We thank Reviewer 2 for their overall enthusiasm and helpful advice for this manuscript.

Major points:
1. Although C. elegans is a useful model organism in cell biological studies, it is quite different from mammals (or humans). Therefore, to better benefit the scientific society, I recommend the authors to discuss how their findings are associated with higher organisms (e.g., the role of SMNK-1 / CK1e in human spermiogenesis or infertility).

Thank you for your advice. We have added this content to the beginning of the discussion section.

2. The statistics section is missing in this manuscript. What statistical methods (t test?) are employed to compare the difference between two groups?

Thank you for the advice. We have added the statistical methods at the end of the METHODS DETAILS section.

3. The quality of figures should be systematically improved. For instance, in Figure 1B, the coloring was wrong in the last group. In Figure 3C (or 3D, or 6C), the positions of lines should be refined. In figure 7C or 7D, the positions of words were quite disordered.

Thank you for your advice; we have revised these in Figure 3 and 7.

Minor points:
1. It would be better to show the mean ± SD rather than SEM, as SD reflects the deviation of samples in a group (e.g., in Figure 1B, 1D, 1E…)

Thank you for your advice; we have revised this in the manuscript.
*2. Line830: The format of symbols should be identical in figures and figure legends. For example, '****p<0.0001'; 'n.s.'…*
Thank you for your advice; we have revised this in the manuscript.

3. The temporal scale of the two supplementary movies is lost.
Thank you for your advice; we have revised this in the manuscript.

4. Typing error: (line 112) 'Ser / Thr.'

Thank you for your advice; we revised this typing error.

5. Figure 1B: "RNA expression" …
Thank you for your advice; we have revised this error.

Reviewer #3 (Remarks to the Author):

I co-reviewed this manuscript with one of the reviewers who provided the listed reports. This is part of the Nature Communications initiative to facilitate training in peer review and to provide appropriate recognition for Early Career Researchers who co-review manuscripts.
We thank Reviewer 3 for their overall enthusiasm and helpful advice for this manuscript.

Reviewer #4 (Remarks to the Author):

In this paper the authors identify SMNK-1 and show that it is necessary for fibrous body assembly during C. elegans spermatogenesis. They provide evidence that the kinase activity of SMNK-1 is required for this function. Additionally, they investigate whether there is a relationship between SMNK-1 and SPE-18 and test whether VHA-

18 has a role in C. elegans spermatogenesis. This is a solid paper and the methodology meets, and in some cases exceeds, the standards in the field. Characterization of two new factors required for spermatogenesis, SMNK-1 and VHA-18, is of interest and importance to the field. Minor points below are suggested for polishing the manuscript and improving readability and ability to interpret the data. The only concern is that the conclusions somewhat overreach what can reasonably be determined by the data presented as noted in the comments below:
We thank Reviewer 4 for their overall enthusiasm and helpful advice for this manuscript.

1. Figure 2A is insufficient to determine if the NKB-2 signal is reduced or to determine whether the hermaphrodite-derived sperm from the smnk-1 mutants are activating in vivo or if there are any defects in MO fusion. While later data support the defects in activation and pseudopod formation of sperm from males, I see no convincing differences between control and mutant in Figure 2A. The legend refers to the "number of GFP puncta" but there was no quantification of the data. I also did not see a methods section related to this (and Figure S2); what age range is a young adult? To better determine the effect of the mutation on hermaphrodite sperm the authors should consider dissecting the hermaphrodite sperm out of the spermatheca.

We sincerely appreciate your valuable advice, which has greatly helped improve our manuscript. We recognize that our initial conclusions were overly absolute and have revised the sentences as follows:

"In the enlarged spermatozoa region in Fig. 2A, the GFP::NKB-2 signal does not appear to be especially reduced in the *smnk-1* mutant when compared with the control. This provided a cue for us to test the impact of the *smnk-1* mutation on spermatogenesis." (see lines 195–198)

Regarding the "number of GFP puncta," we acknowledge the challenge of accurately counting within the hermaphrodite uterus and have revised the relevant text to address this technical consideration.

We are grateful for the reminder to clarify the methodological details. We have supplemented the methods for Figure 2A and Figure S2 and confirm that the young adults in Figure 2A were analyzed at 12–16 h post-L4 stage. In addition, we attempted to dissect hermaphrodite sperm from the spermatheca, but regrettably, we were unable to obtain clear images for reliable analysis.

2. I fully agree that there is reduced MSP seen in the mutant in Figure 3A. However, I'm not sure the authors convincingly show that this is entirely due to failure to assemble into FBs and not due to reduced expression or stability of MSP. The RNAseq data in S3 shows a log2fc below 1 for all msp genes, and while it's stated the change is not significant at least msp-3 appears below 0.5 which I believe would be

considered significant. qRT-PCR would be better to look at all these genes, and the authors should also take into account that collectively small reduction in every gene could lead to an overall reduction in protein. In 3C the authors state MSP is "completely dispersed in the cytoplasm" (line 233), however their description in the figure legend that it is "diffusive in the cortical area" is more accurate. They also say that part of the MSP is retained in the residual body, but based on the images shown it does not appear that there is more MSP in the residual body of the mutant than there is in the control. Ultimately, it's not clear if it is a partitioning defect which is what is concluded in lines 250 – 251.

Thank you for this insightful advice. We cannot fully rule out that the SMNK-1 mutation affects MSP expression, not just assembly. In our RNA-seq analysis, using a threshold FC of *(him-5/smnk-1*) ≥2 ($\log_2$ FC ≥ 1), each MSP (including MSP-3) exhibited no significant differences. To clarify, we adopted a new presentation format with the significance threshold defined as FC (*smnk-1;him-5/him-5*) ≥2 or ≤0.5, which was used to replace the previous criterion. We considered qRT-PCR for MSP levels, but designing specific primers is challenging because of the high sequence similarity among the MSP molecules.

We agree with the perspective that a collectively small reduction in every gene could result in an overall reduction in protein. Based on this interpretation, we have refined our conclusions regarding the functional role of SMNK-1 in MSP expression and assembly. (see lines 275–282)

Thank you for this suggestion; we realized that the description of MSP localization in the *smnk-1* mutant was not sufficiently precise and have therefore amended the relevant statements in the revised manuscript.

3. The data in Figure 5 is not sufficient to conclude that SMNK-1 recruits SPE-18 to the surface of MOs. It clearly has an effect on SPE-18 localization/puncta formation, but this effect can be indirect and may regulate some other aspect of SPE-18 association with MOs/FBs other than recruitment. It's worth noting that here the intensity of the signal remains bright even though puncta don't form, which is not what was observed for MSP in the smnk-1 mutant. The authors should tone down the conclusion that SMNK-1 "recruits" SPE-18 throughout the manuscript (including lines 459 – 461). They also show no evidence that SMNK-1 phosphorylates SPE-18, and while this is a viable hypothesis they should be careful how they discuss this in the Discussion section (line 432 is not supported by any data presented).

Thank you for your suggestion. As you mentioned, we cannot confirm a direct recruitment of SPE-18 by SMNK-1 and have revised the statement to "SMNK-1 functions as an upstream protein kinase of SPE-18, and its kinase activity is essential for the localization of SPE-18 on the MO surface in spermatocytes." (see lines 490–492)

Regarding the Discussion section, we have modified the expression to frame it as a

possibility.

4. In their discussion of VHA-18 the authors should cite the spe-5 papers (SPE-5 is a v-ATPase B subunit) and discuss their results in the context of similarities and differences between the vha-18 and spe-5 phenotypes. Note, treatment with bafilomycin was also done in the 2012 spe-5 paper.

Thank you for your advice; we have cited the reference and added related context in this manuscript, as part of the Results and Discussion sections. (see lines 356–359 and lines 523–530).

"SPE-5 exhibits the highest degree of similarity to VHA-18. SPE-5 and VHA-18 both affect MO morphology and cause MO vacuolation beginning at the spermatocyte stage. Loss of either gene results in multinucleate spermatocytes, MSP sorting defects during the budding stage, and abnormal sperm activation. However, the severity of their phenotypes differs. The *spe-5* hermaphrodites were almost completely sterile, producing few or virtually no functional sperm, and spermatogenesis was typically arrested at the spermatocyte stage, with individual cells containing four haploid nuclei. In contrast, *vha-18* mutants rarely exhibited multinucleate (or multiple chromatin masses) spermatocytes, and hermaphrodites typically produced around 100 progenies, indicating substantially higher fertility. Furthermore, *spe-5* mutants revealed a cold-sensitive phenotype, with more severe sterility at 16°C, a feature that has not been examined in *vha-18*. In addition, intact FBs were detected in *vha-18* spermatids (Supplementary Fig. 7A), but no such phenotype was observed in *spe-5* mutants. Previous studies on SPE-5 relied on antibody staining to assess its expression pattern, whereas our CRISPR–Cas9–generated VHA-18:mCherry knock-in strain enabled more accurate and convenient visualization of its expression stage and pattern in vivo. Due to its relatively high fertility, *vha-18* represents a more suitable model for investigating MO development."

5. The authors conclude that the reduced MSP in the smnk-1 mutant spermatids is the reason they fail to activate (line 398), however some MO fusion is seen indicating that partial activation occurs and while the reduced MSP would affect pseudopod size/formation they do not provide evidence how MSP may affect MO fusion or prevent activation (vs the activated sperm can't form a pseudopod).

Thank you for your insightful advice; our previous statement was insufficiently rigorous. Indeed, *smnk-1* mutant sperm cannot be fully activated to form complete pseudopods or achieve sufficient MO fusion. Therefore, current evidence cannot establish a definitive link between pseudopod formation and adequate MO fusion. The relevant statements have been amended by removing these unverified causal relationships in a revised manuscript.

6. The authors conclude that MSP assembly into FBs depends on membrane from the MOs (line 449 – 451), but it's not clear which data they are drawing this conclusion from.

Thank you very much for your careful review and for pointing out this issue. Currently, we cannot conclude that MSP assembly into FBs depends on membranes derived from the MOs. Our data indicate that the MO membrane of *vha-18* mutant sperm influences MSP sorting, which leads to the mislocalization of a portion of MSP into the residual body, and was also reported in *spe-5* mutants. However, we did not observe any direct effects on FB assembly. Therefore, we have removed the relevant statements from the revised manuscript.

7. Based on the DAPI staining in Figure 3D, there are noticeable differences in the germline/nuclear staining/presence of spermatocytes in the mutants compared to control, especially smnk-1(ibp116). The authors should address this and a similar image of the germline should be shown for the (ibp107) allele. It would be helpful if DIC images of the germlines were shown as well.

Thank you for your advice; we have included DIC images in Figure 3D.

As for the MSP staining of the *smnk-1(ibp107)* germline, we tried several times to obtain clear images; however, the permeabilization effect on the germline was not sufficient. Therefore, we supplemented it in Figure R3. We observe diffused MSP fluorescence in the *smnk-1(ibp107)* mutant group, whereas assembled FB structures are present in the *him-5* control group, which is consistent with the other two alleles in Figure 3D.

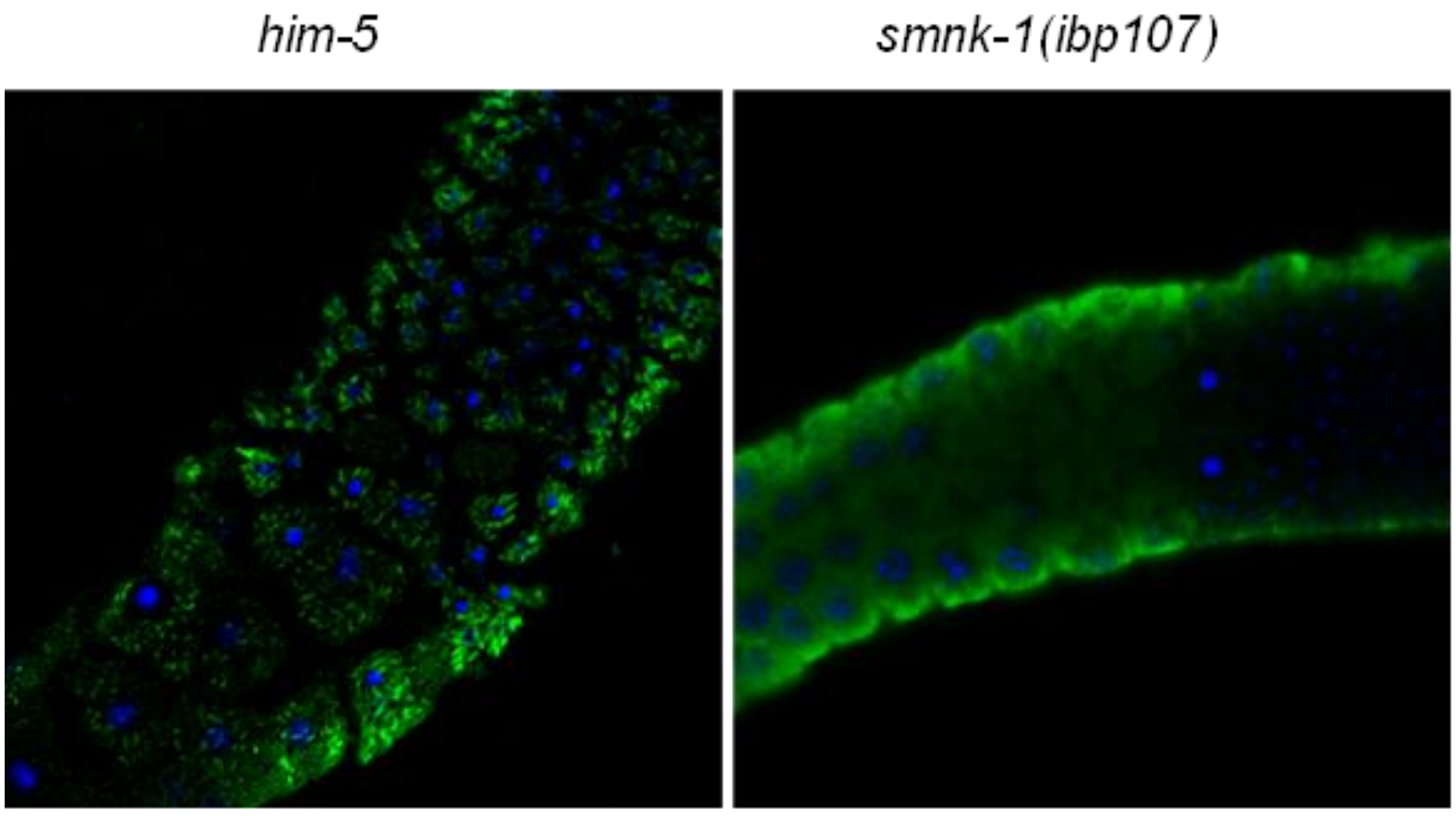

Figure R3

Other minor comments:

*• Line 35 in Abstract: What is meant by "**vectorial** transport"? Did the authors mean*

"vesicular transport"?

We want to express that this transportation is directional, but it may be misunderstood; therefore, we have revised this expression. Thank you for the advice.

• In the introduction section, the authors mention human infertility. Are there any known roles for casein kinases in human sperm? If so, the authors should cite these.

Thank you for your advice; we have supplied the known roles of casein kinases in human sperm at the beginning of the Discussion section.

• Lines 40 and 53: "sperm infertility" doesn't make sense since it is the organism and not the sperm that is "fertile" or "infertile". Perhaps "infertility due to defects in sperm"?

Thank you for the advice; we have revised this expression.

• Typo in Figure 1D, second and fifth sets of data points labeled "smnk-(ibp107);him-5" should be "smnk-1(ibp107);him-5"

Thank you for your advice; we have amended this error.

• Line 124: "progenies" should be "progeny"

Thank you for the advice; we have revised this expression.

• Difference in scale bars between Figure 1D and 1E make it difficult to tell if males are more severely sterile/sub-fertile than the hermaphrodites. The authors state in the text the average number of hermaphrodite self-progeny for 1D, it would be helpful if they could similarly state the average number of cross-progeny produced by the mutant males.

Thank you for your advice; we have revised this content in Figure 1E and the text.

• How were the zones determined for Figure 1F? This is not mentioned in the results or methods sections. The cartoon and graph are not mentioned in the figure legend.

Thank you for this suggestion. Figure 1F primarily shows the uterine regions on both sides of the vulva, and we have labeled the position of the vulva in Figure 1F in the revised manuscript.

The zones extending from the vulva to the spermatheca were divided into three equal segments. We then quantified the percentage of sperm within the four distinct zones, including the seminal vesicle zone. We have added the corresponding description in the Figure 1F legend.

• Supplementary Figure 1A: the triangle does not actually point to the T.

Thank you for your advice; we have corrected this content in Supplementary Figure 1A.

• Please indicate which allele(s) of smnk-1 are used in Figure 2.

Thank you for your advice. We have revised this content in the manuscript. Specifically, for the *smnk-1* allele shown in Figure 2 and other unlabeled instances, it is *ibp107*, which is a frameshift mutation.

• Missing the figure legend for Supplementary figure 2B.

Thank you for your suggestion; we have added a figure legend for Supplementary Figure 2B.

• Line 193: when the authors say "remained still" do they mean the sperm were not moving?

Yes, "still" means not moving, and we have amended this to "immobile" to avoid confusion.

• Figure 2D: the smnk-1 sperm looks smaller and the MOs more compact. Is this just the representative image? Because it also seems like the sperm might be smaller in Figure 2A.

Yes, as you noted, the smaller size of *smnk-1* sperm was observed in most images, and not just the representative image. This is probably because the MOs look more compact.

• It seems like there's some polarity to the fused MOs in Figure 2F that isn't really seen in Figure 2C. I wonder if it would be more accurate to use FM1-43 to quantify the number of fused MOs rather than relying on GFP::NKB-2 which could itself be affected by smnk-1? Alternatively, could the authors co-stain with lysotracker to also identify the unfused MOs in the Pronase exposed sperm? The assumption is that all fuse in the control, while some fail to fuse in the mutant.

Thank you for your valuable advice. We have supplied this assay, using FM1-43 and GFP::NKB-2 to quantify the number of fused MOs, as shown in Figure R4 and Figure R5. We found no obvious difference between these two labeling methods to indicate fused MOs.

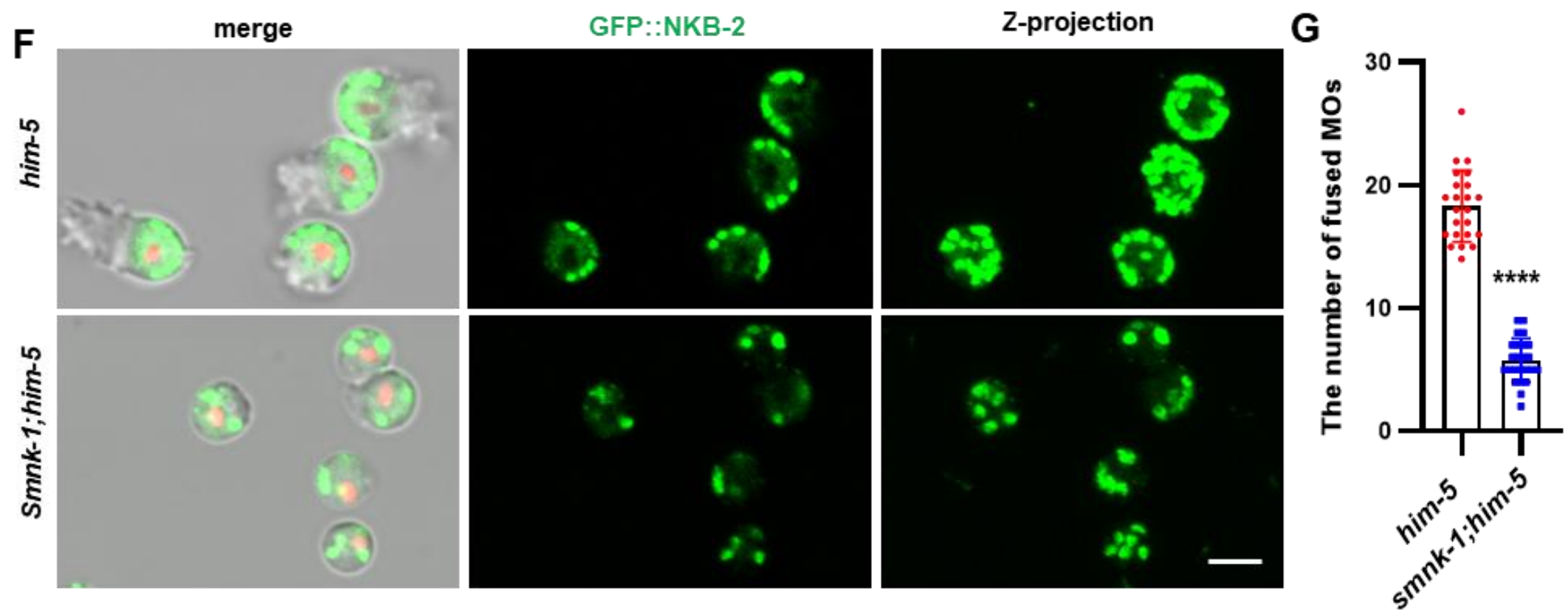

Figure R4 (Main Figure 2F-G)

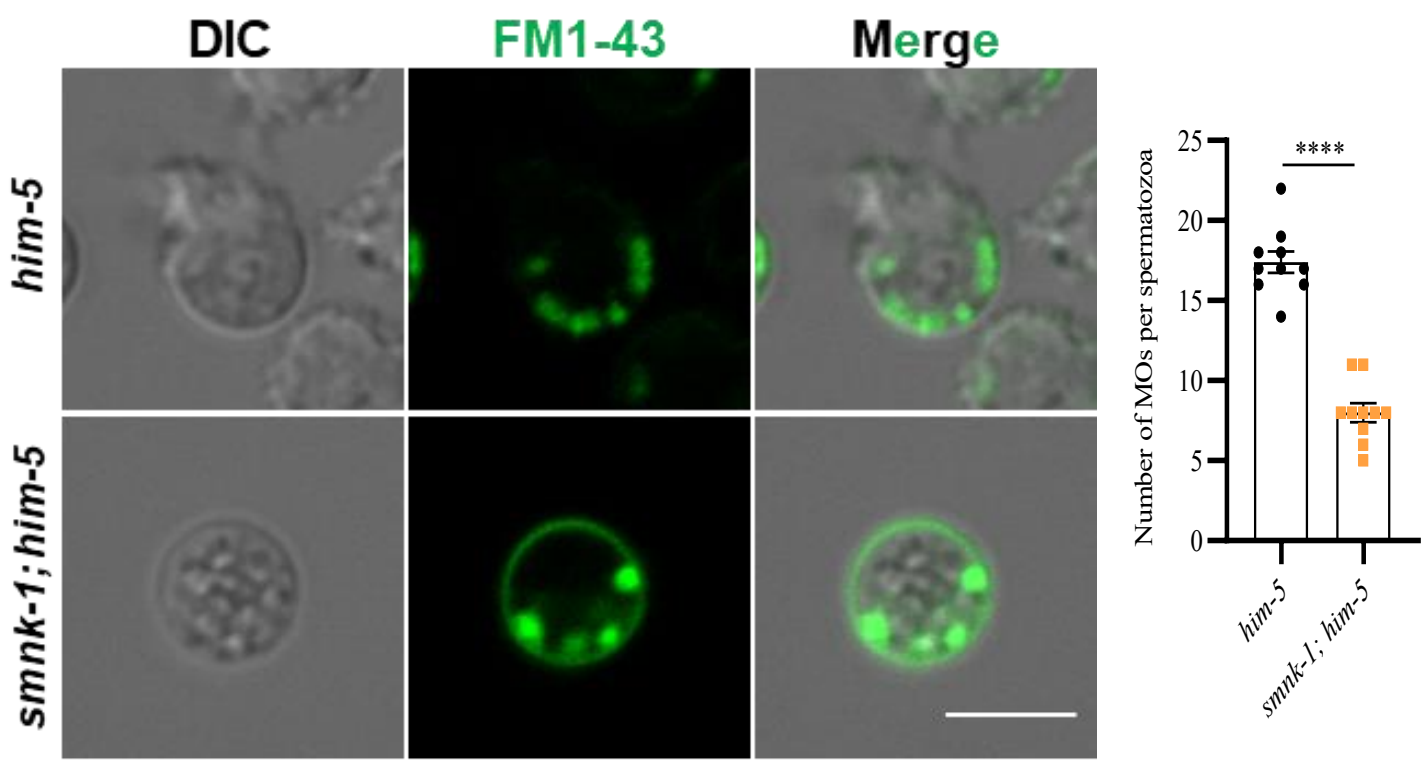

Figure R5

• I am not particularly convinced by Figure 3G. The authors do not show with any other MO marker that some MOs or MO components are abnormally segregated to the residual body in the smnk-1 mutant. And they show with their own quantification that the number of MOs in the spermatids is not reduced compared to control (Fig 2E).

Thank you for pointing this out. Reviewer 1 raised the same concern. We have revised the legend of Fig. 3G (see lines 1039-1042), and we have added the interpretation for the budding Figure 3G in the manuscript. (see lines 272–274)

• Lines 255 and 256: Don't need "the" before mitosis or meiosis.

Thank you for the comment; we have revised this expression.

• I assume Figure 4A is from a wild-type animal? This should be clearly stated in the figure legend.

Yes, as you mentioned, Figure 4A is from a wild-type animal. Thank you for your suggestion. We have labeled the WT in Figure 4A and the Figure 4A legend.

• In Figure 4 it would be helpful for readers if in panels B and C it was clearly labeled that the green signal is SMNK-1 (without having to refer to figure legend).

Thank you for your suggestion; we have labeled it so in Figures 4B and C.

• Figure S4: The signal is surprisingly bright in the spermatozoa compared to the spermatids. Autofluorescence in the green channel is known to be high in the cell body of the spermatozoa. Showing a negative (no GFP) control would be helpful to know how much signal is from autofluorescence vs GFP. DIC to know where the pseudopod is would also be helpful.

Thank you for this great advice. In the latest experiment, we observed that after removing the autofluorescence, the SMNK-1::GFP spermatozoa exhibited only faint green fluorescence. The relevant content has been revised accordingly in the manuscript.

• Figure 4C: It is a bit of a stretch to say that the GFP signal "surrounded" the MSP signal. They do seem to be associated, but the red is not completely enclosed within the green. A more magnified inset of this image would be helpful. In Figure S4C there is only minimal SMNK-1 colocalization with 1CB4. The green and red signals are mostly distinct with limited yellow appearing in the merged image (magnified 2).

Thank you, we appreciate the constructive criticism regarding the spatial correlation between SMNK-1::GFP, MSP, and the 1CB4 labeled-MO. We have thoroughly revised the relevant sections of the manuscript according to your advice.

To our knowledge, the SMNK-1::GFP signal is not uniformly distributed along the periphery of the FBs; instead, it displays marked enrichment at both termini of the boat-shaped structures. We have provided more immunofluorescent images of SMNK-1::GFP and MSP in spermatocytes, which were also included in the Supplementary Figures. To more clearly illustrate this distinct spatial patterning of SMNK-1::GFP and MSP in spermatocytes, we have included a schematic diagram in Figure 4C.

Based on the current immunofluorescent images, we conclude that SMNK-1 only partially colocalizes with MOs, which are indicated by the 1CB4 antibody. We have revised the related descriptions in the manuscript.

• Line 298: They appear to partially co-localize in the karyosome stage, not late pachytene (Fig 5 A and B, colocalization is seen in box c but not box b).

We appreciate your comment; we have revised this according to your advice.

• Can the authors differentiate between defective vesicular acidification vs morphological defects and improperly formed MOs (which they show by EM) using lysotracker staining (lines 341 – 344)? Similarly Figure 6D seems to show a loss of SMNK-1::GFP rather than altered localization.

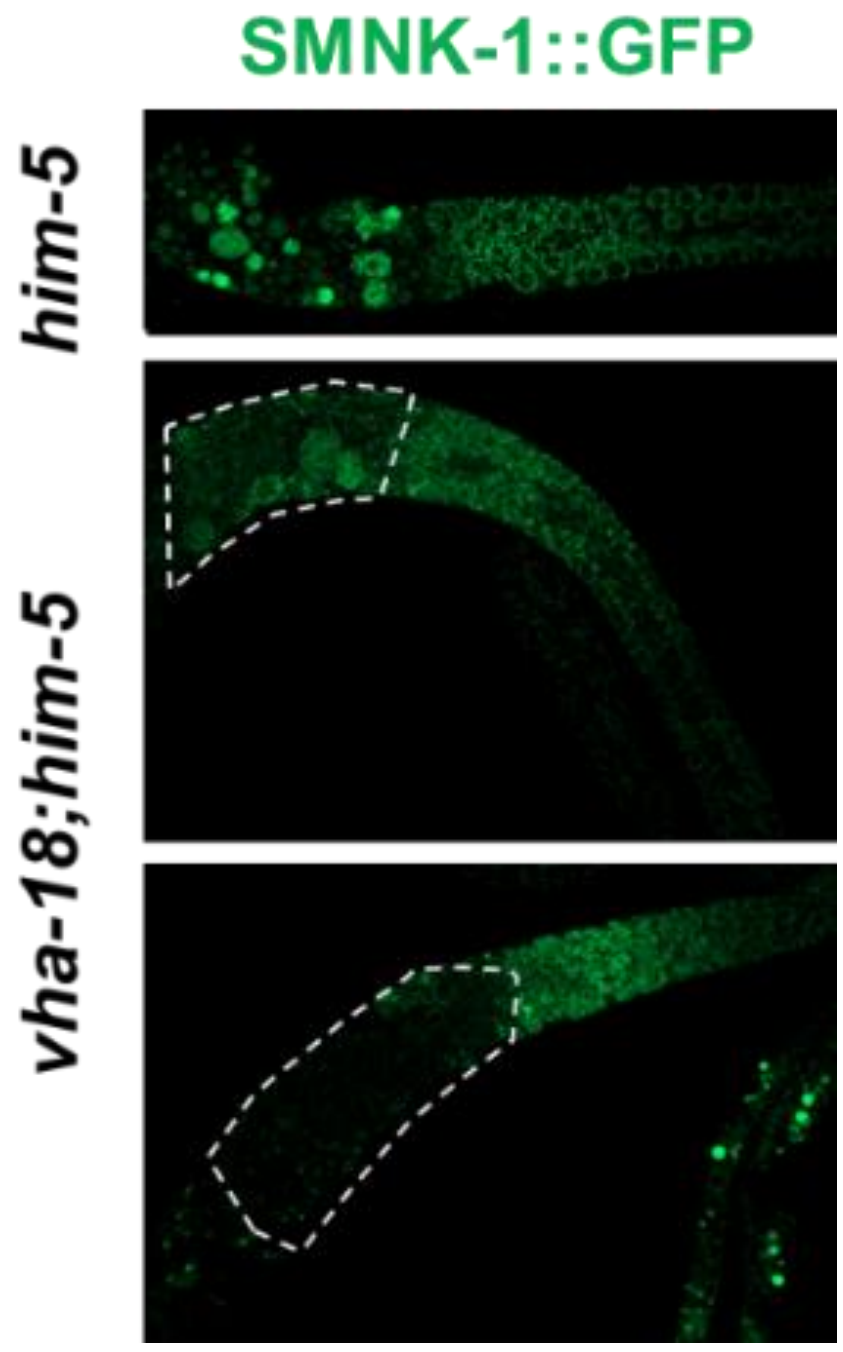

Figure R6

We sincerely appreciate your insightful comment. We agree that LysoTracker staining alone cannot distinguish between vesicular acidification and morphological defects; however, LysoTracker staining revealed abnormal acidification patterns. In addition, our TEM analysis provides evidence that MO morphology was profoundly altered in *vha-18;him-8* mutant sperm, revealing swollen and abnormal vesicular structures (Figure 7B), thus supporting a primary structural defect. This suggests that the v-ATPase defect directly affects MO integrity. We have revised the corresponding description as shown in lines 384–390.

Regarding the localization of SMNK-1::GFP, we repeated this experiment numerous times. We found variation in the SMNK-1::GFP signal within the germline of *vha-18; him-5* mutants, with some individuals displaying only a modest reduction, whereas others exhibited a significant decrease compared to the control group, like the above figure. We suppose that this discrepancy may arise from the differential effects of the *vha-18* mutation across individuals, but all images showed reduced SMNK-1::GFP signals to different degrees (see Figure R6).

• I'm not sure what is being show in Figure 7A(b). This is control, but no MO membrane is indicated and the FBs look very different from (a).

Thank you for your valuable advice. Figure 7A(a) and Figure 7A(b) are both the control group, and Figure 7A(c) and Figure 7A(d) are both the *vha-18* mutant group.

We identify the developmental stage based on the size of FB-MO. Therefore, Figure 7A(a) and 7A(c) represent an earlier developmental stage, during which the assembled FB–MOs are relatively small. In contrast, Figure 7A(b) and 7A(d) correspond to a later stage, where the FB–MOs are larger than those in Figure 7A(a) and 7A(c).

We acknowledge that the original labeling is not clear, and we have revised the figure legends accordingly.

Furthermore, the apparent morphological variations in the FB–MO observed in Figure 7A may be reflected better by TEM imaging. Therefore, we have replaced the original image with a more representative micrograph showing the typical structural features at this developmental stage.

• Line 376-377: Figure 7C shows that the MSP looks different, and more MSP ends up in the residual body, but does not convincingly show there is less MSP in the spermatids.

Thank you for this valuable suggestion. We replaced this figure with another, more representative image with significant MSP in the residual body. Our data indicated that most of the MSPs are deposited into the RB, whereas some remained in the budding spermatids. We did not find a reduction of MSP in the spermatids of the *vha-18* mutant. We have removed the misleading description in the manuscript.

• Maybe move the 1CB4 staining from S4 to the main figure? This data is important for conclusions drawn. Similarly, S6D and S6E feel like they should be in the main figure.

Thank you for your advice. We have moved these figures to the main Figure 7B and systematically reorganized the corresponding textual content to ensure logical coherence.

• Figure 7D: Is there a reason the author chose to activate with TEA instead of pronase or zine which tend to be more robust in vitro activators?

Thank you for this query. Although pronase and zinc both activate sperm robustly, very few of the resultant spermatozoa migrate, whereas most of the spermatozoa activated by TEA crawl on the substrate, similar to the dissected spermatozoa that were activated *in vivo*.

• Cite MSP constitutes 80% of proteins in the pseudopod (line 426 – 427).。

Thank you for your advice. We have cited the reference L'Hernault, S.W. (1997). Spermatogenesis. In *C. elegans* II. Cold Spring Harbor Laboratory Press, Cold Spring Harbor (NY), pp. 271–294, and revised the manuscript as suggested. "MSP

constitutes 10%–15% of the total protein and 40% of the soluble cellular protein in sperm. Notably, MSPs make up approximately 80% of the pseudopods' total protein" (see lines 482–484).

Reviewer #5 (Remarks to the Author):

I co-reviewed this manuscript with one of the reviewers who provided the listed reports. This is part of the Nature Communications initiative to facilitate training in peer review and to provide appropriate recognition for Early Career Researchers who co-review manuscripts.

We thank Reviewer 5 for their overall enthusiasm and helpful advice for this manuscript.

Ma et al. ***Nature Communication*** Tracking #: NCOMMS-25-11037A

Response to reviewer comments

We sincerely thank the three reviewers for their positive and constructive feedback on our manuscript. We greatly appreciate their recognition of our findings regarding the co-regulation of FB-MO complex assembly and transport by SPE-55 and VHA-18 during *C. elegans* spermatogenesis.

We have thoroughly revised the manuscript in accordance with all reviewers' comments. Below, we provide detailed point-by-point responses: reviewer comments are reproduced in blue italic font, and our responses are presented in black regular font.

We believe these revisions have significantly improved the manuscript. We hope the revised version meets the standards for publication in ***Nature Communications***.

REVIEWER COMMENTS

Reviewer #2 (Remarks to the Author):

The revision has improved the manuscript and I recommend its publication in Nature Communications.

We sincerely thank Reviewer 2.

Reviewer #3 (Remarks to the Author):

I co-reviewed this manuscript with one of the reviewers who provided the listed reports. This is part of the Nature Communications initiative to facilitate training in peer review and to provide appropriate recognition for Early Career Researchers who co-review manuscripts.

We sincerely thank Reviewer 3.

Reviewer #4 (Remarks to the Author):

The authors have addressed a number of our concerns and made significant revisions. There are still a few outstanding issues that we feel ought to be addressed before publication.

We sincerely thank Reviewer 4 for their overall enthusiasm and valuable suggestions regarding our manuscript.

Major:

Line 109 – 110: Regarding Reviewer 1 comment #2. I can find no evidence that the smnk-1 gene name is an approved name for C09B9.4. smnk-1 does not show up on WormBase or the Genome Alliance database or Uniprot. All instances of smnk-1 in the manuscript should be replaced with spe-55, which is the community approved and searchable name for this gene.

Thank you for your helpful suggestion. We have replaced *smnk-1* with *spe-55* throughout the manuscript and corresponding figures. The gene *spe-55* is listed in WormBase.

Line 490 – 492: Revise to "SMNK-1 functions upstream of SPE-18 and its kinase activity is essential for the localization of SPE-18 on the MO 492 surface in spermatocytes." To state that SMNK-1 functions "as an upstream protein kinase of SPE-18" implies direct regulation and phosphorylation that were not shown in this study.

Thank you for your suggestion. We have revised our manuscript.

(Lines 508-510)

Minor:

Line 62 – 68: I understand the authors were trying to improve the accuracy of their statement, but this sentence is confusing as written. We suggest at least simplifying to: "During the meiosis II sorting stage of spermatogenesis in C. elegans, the developing spermatid containing DNA, some mitochondria, and membranous organelles (MOs) is

budded away from the residual body (RB), where unnecessary components, such as the endoplasmic reticulum, ribosomes, tubulin, and actin, are disposed.

Thank you for this helpful suggestion. We have revised the sentence to improve clarity and simplify the description of the meiosis II sorting stage, largely following the reviewer's recommendation (Lines 62–67).

Fix gene labels in Sup Fig S1A.

Thank you for your helpful suggestion. We have fixed corresponding gene labels in Fig S1A.

Title and legend for Sup Fig S2 should be revised. The whole manuscript presents data that FB assembly during spermatogenesis requires SPE-55, so saying it does not affect spermatogenesis is inaccurate. I believe the authors mean to say that it does not affect the number of spermatids produced.

Thank you for pointing this out. We agree that the previous wording was misleading, as our data demonstrate that SPE-55 is required for FB assembly during spermatogenesis. We have therefore revised the title and legend of Sup Fig S2 to state that *spe-55* does not affect the number of spermatids produced.

Line 154: Remove "rescue" language here and elsewhere. In cases of sperm-specific defects hermaphrodites can produce normal numbers of progeny when mated to wildtype males, but this is not a "rescue" experiment. The genetic defect of the hermaphrodite is not rescued.

Thank you for pointing this out. We agree that the term "rescue" is inappropriate in this context, as the genetic defect of the hermaphrodite is not corrected. We have replaced "rescue" with more accurate wording (e.g., "restored to wild-type levels" / "complemented by mating with wild-type males") throughout the manuscript (Lines 161, 445, and 832).

Lines 192 to 198 need to be rephrased. Simply reading them is confusing. The authors first state that there was no difference in hermaphrodite spermatids before ovulation and after ovulation the spermatids activated. This implies that spermatogenesis and sperm activation are unaffected in the hermaphrodite. But then they end the paragraph that the data "provided a cue for us to test the impact of the smnk-1 mutation on spermatogenesis". While we think it is important that the authors

acknowledged our initial concern that the data are insufficient to determine if the NKB-2 signal is reduced, it does not logically transition to the conclusion that they should look at spermatogenesis in more detail or that they should look at male-derived sperm.

Thank you for your suggestion. We have revised our manuscript (Line202-208). We observed some difference in GFP::NKB-2 fluorescence after ovulation（though may be not significant), combined with the fact that the mutant male sperm have severe motility defects after mating with WT hermaphrodites. All these provided a cue for us to test the impact of the *spe-55* mutation on spermiogenesis.

Lines 200-203: The sentence repeats itself, and without quantification the authors can't definitively say they have a "normal number". Shorten to: "There was no obvious difference in the number of spermatids produced between the mutant and WT animals (Supplementary Fig. 2B)."

Thank you for this helpful suggestion. We have revised the sentence as suggested to eliminate repetition and avoid overinterpretation (Lines 210–211).

From our comment in the original review: Please indicate which allele(s) of smnk-1 are used in Figure 2. The allele name is now mentioned in the beginning of the results sub-section, but not in the figure or the figure legend itself. We recommend the explanation given in the rebuttal, "for the smnk-1 allele shown in Figure 2 and other unlabeled instances, it is ibp107, which is a frameshift mutation", should be mentioned in the manuscript to avoid confusion.

Thank you for your suggestion. We have revised our manuscript (Line 134-135).

Figure 6 – stage of spermatocytes being shown should be specified in figure legend (per comment from Reviewer 1)

Thank you for the suggestion. As requested, we have updated the Figure 6 legend to specify the developmental stage of the spermatocytes.

Line 372: Authors cite Reference 23 when it should be Reference 22

Thank you for pointing this out. We have revised the citation in our manuscript as suggested.

Line 377: Per Reviewer 1 "Based on several other Spe mutants, the "large abnormal vacuoles" are undoubtedly swollen MOs. It would be worth noting that similar structures have been previously reported in other Spe mutants." While the authors changed the language to refer to the observed structures as swollen MOs, they failed to note that similar structures have been reported previously or provide any citations to these previous observations that inform their conclusion.

Thank you for this helpful comment. We have clarified that similar swollen MOs have been described previously in other *Spe* mutants and have added the relevant citations to support our conclusion (Line 398).

Line 392: There is no Supplementary Figure 6H

We sincerely apologize for this error. Upon rechecking, we confirmed that Supplementary Figure 6H does not exist. The graph of *him-8*(upper)and *vha-18;him-8*（lower）all belongs to Supplementary Figure 6F. we have added related description to make it clearer in the figure legend of Supplementary Figure 6F(Line 1314-1316).

Line 395 – 397: It is not clear what is meant by "exhibited less of a reduction compared to that of the control group". The language provided in the Response to Reviewers is much clearer. We suggest using this same language in the manuscript: "we also observed variation in the SMNK-1::GFP signal within the germline of vha-18;him-5 mutants, with some individuals displaying a modest reduction, whereas others revealed a significant decrease compared to the control group."

Thank you for the excellent suggestion. We agree that the suggested phrasing is much clearer, and we have updated the manuscript with this exact wording. (Lines 396-399).

Reviewer comments not addressed, even though rebuttal says they were:

Line 124 – reference Figure 1D for statement about brood size. Now lines 135 -138.

Thank you for pointing this out. We have revised description "*spe-55(ibp107)* hermaphrodites exhibited no significant difference compared with the control *him-5* hermaphrodites when mated with *him-5* males, which indicates that the *spe-55* infertility defect is due to defective sperm and not defective oocytes." in the

manuscript. (Lines 141-144) and point the specified brood size in manuscript(Line 136-140, 149-154) and figure legend(Line 996-1004)

Line 50-52. Add Smith (2006) citation here (already in references). This reference is now completely omitted. Should be cited on line 58 of revised manuscript.

We thank the reviewer for pointing out this oversight. We agree that the work of Smith (2011) is highly relevant here. We have incorporated this reference into the revised manuscript. (Line 59)

Line 57-61. Move Hu citation to this sentence. Add two references

1) Winter ES, Schwarz A, Fabig G, Feldman JL, Pires-daSilva A, Müller-Reichert T, Sadler PL, Shakes DC. (2017). Cytoskeletal variations in an asymmetric cell division support diversity in nematode sperm size and sex ratios. Development 144(18):3253-3263. doi: 10.1242/dev.153841.

2) Roberts TM, Pavalko FM, Ward S (1986) Membrane and cytoplasmic proteins are transported in the same complex during spermatogenesis. J Cell Biol 102 (5): 1787–1796.

None of these citations are in the correct place. Should be cited at the end of line 68 of revised manuscript.

Thank you for your helpful suggestion. We have added these references into the revised manuscript.

Difference in scale bars between Figure 1D and 1E make it difficult to tell if males are more severely sterile/sub-fertile than the hermaphrodites. The authors state in the text the average number of hermaphrodite self-progeny for 1D, it would be helpful if they could similarly state the average number of cross-progeny produced by the mutant males.

Thanks for your helpful advice, we have revised the Figure 1D and 1E to make mean value and error bar clearer, Meanwhile, we have stated the average number of hermaphrodite self-progeny for 1D in manuscript (Line 136-140, 149-154) and figure legend (Line 996-1004); We also added the average number of non-dumpy (cross-progeny) and dumpy (self-progeny) into the revised manuscript (Line1008-1016).

Ma et al. ***Nature Communication*** Tracking #: NCOMMS-25-11037B

Response to Reviewer Comments

We sincerely thank the reviewers for their thorough evaluation and constructive comments on our manuscript. These critical and thoughtful suggestions have been invaluable for improving the clarity, accuracy, and completeness of our work. We have carefully addressed each comment in detail and implemented all necessary revisions. Below, we provide our point-by-point response: reviewer comments are reproduced in blue italic font, and our responses are presented in black regular font. We believe that all issues raised by the reviewers have been fully addressed, and the manuscript has been significantly improved. We hope the revised manuscript is now suitable for publication in ***Nature Communications***.

REVIEWER COMMENTS

Reviewer #4 (Remarks to the Author):

We sincerely thank Reviewer 4 for their overall enthusiasm and valuable suggestions regarding our manuscript.

1. The authors have replaced smnk-1 with spe-55 throughout the manuscript and corresponding figures. However, they did not replace smnk-1 in the REAGENT or RESOURCE SOURCE IDENTIFIER. A simple search shows 26 instances of "smnk-1" still in the manuscript in the strain list, oligonucleotides, and recombinant DNA.

We sincerely apologize for this incomplete revision. We have now performed a full-text search of the entire manuscript and completely replaced all remaining instances of "*smnk-1*" with the community-approved gene name "*spe-55*"

2. Lines 508-510 are "MOs' lumen, thereby making the immediate cytoplasmic microenvironment around MOs more alkaline. This result is consistent with a previous study in which a higher pH is favorable for MSP polymerization at the leading edge of". This is not the text the authors claim to have revised which was meant to be "SMNK-1 functions upstream of SPE-18 and its kinase activity is essential for the localization of SPE-18 on the MO surface in spermatocytes."

Thank you for your helpful suggestion. We have now corrected Lines 508–510 exactly as recommended by the reviewer. The relevant text has been revised to: SPE-55 functions upstream of SPE-18 and its kinase activity is essential for the localization of SPE-18 on the MO surface in spermatocytes.

3. Where it was suggested the authors indicate which allele(s) are used in Figure 2 the added sentence "For the spe-55 allele shown in other unlabeled is ibp107 allele." could use to be revised for clarity. [Please also note this is lines 132-133, not 134-135

as stated in the rebuttal.]

We thank the reviewer for clarifying the line numbers and for pointing out the unclear wording. We have revised the sentence for grammatical accuracy and improved clarity. The revised statement reads: The *spe-55* allele used throughout this study, including in all unlabeled panels, is *ibp107*, a frameshift mutation.

4. Previous comment: Line 50-52. Add Smith (2006) citation here (already in references). This reference was then omitted in the revision. Should have been cited on line 58 of revised manuscript.

Author reply: We thank the reviewer for pointing out this oversight. We agree that the work of Smith (2011) is highly relevant here. We have incorporated this reference into the revised manuscript. (Line 59)

Note: Smith (2011) is not the right reference. Smith (2006) that was referenced in the original manuscript is the reference that should be added back.

We sincerely apologize for this citation mistake. We have now corrected the reference at the appropriate position to Smith (2006), exactly as the reviewer originally recommended. The correct and relevant citation has been restored and matches the reference list.

Reviewer #5 (Remarks to the Author):

I co-reviewed this manuscript with one of the reviewers who provided the listed reports. This is part of the Nature Communications initiative to facilitate training in peer review and to provide appropriate recognition for Early Career Researchers who co-review manuscripts.

We sincerely thank Reviewer 5.
